# Genome-wide analysis of the *Catalpa bungei* caffeic acid O-methyltransferase (COMT) gene family: identification and expression profiles in normal, tension, and opposite wood

**DOI:** 10.7717/peerj.6520

**Published:** 2019-03-14

**Authors:** Nan Lu, Wenjun Ma, Donghua Han, Ying Liu, Zhi Wang, Nan Wang, Guijuan Yang, Guanzheng Qu, Qiuxia Wang, Kun Zhao, Junhui Wang

**Affiliations:** 1 State Key Laboratory of Tree Genetics and Breeding, Key Laboratory of Tree Breeding and Cultivation of State Forestry Administration, Research Institute of Forestry, Chinese Academy of Forestry, Beijing, China; 2 College of Landscape Architecture, Nanjing Forestry University, Nanjing, China; 3 College of Forestry, Northwest A&F University, Yangling, China; 4 State Key Laboratory of Tree Genetics and Breeding, Northeast Forestry University, Harbin, China; 5 Nanyang Research Institute of Forestry, Nanyang, China; 6 Luoyang Academy of Agriculture and Forestry, Luoyang, China

**Keywords:** Tension wood, *Catalpa bungei*, Genome-wide, COMTs

## Abstract

Caffeic acid O-methyltransferase (COMT) is an important protein that participates in lignin synthesis and is associated with the ratio of G-/S-type lignin in plants. COMTs are associated with the wood properties of forest trees; however, little known about the COMT family in *Catalpa bungei*, a valuable timber tree species in China . We performed a comprehensive analysis of COMT genes in the *C. bungei* genome by describing the gene structure and phylogenetic relationships of each family member using bioinformatics-based methods. A total of 23 putative COMT genes were identified using the conserved domain sequences and amino acid sequences of COMTs from *Arabidopsis thaliana* and *Populus trichocarpa* as probes. Phylogenetic analysis showed that 23 *CbuCOMT*s can be divided into three groups based on their structural characteristics; five conserved domains were found in the COMT family. Promoter analysis indicated that the* CbuCOMT* promoters included various cis-acting elements related to growth and development. Real-time quantitative polymerase chain reaction (PCR) analysis showed differential expression among *CbuCOMT*s. *CbuCOMT*2, 7, 8, 9, 10, 12, 13, 14, 21, and 23 were mainly expressed in xylem. Only *CbuCOMT*23 was significantly downregulated in tension wood and upregulated in opposite wood compared to normal wood. Our study provides new information about the* CbuCOMT* gene family and will facilitate functional characterisation in further research.

## Introduction

Caffeic acid O-methyltransferase (COMT, EC:2.1.1.68) is a lignin monomer-specific enzyme that catalyses O-methylation of the C5 hydroxyl moiety of suitably hydroxylated phenolic rings of monolignols, leading to preferential formation of syringyl subunits associated with syringyl (S) lignin monomer synthesis. Trees often have their specific S/G ratio (S-type to G-type lignin monomers) in xylem (Cai et al., 2016); this ratio may be related to the mechanical properties of wood due to the different molecular structures of the monomers (Ozparpucu et al., 2018). Higher G-unit content causes lignin to be more condensed due to larger proportions of biphenyl, phenylcoumaran, and other carbon–carbon linked units because the C5 position on their aromatic rings is available for radical coupling reactions. In contrast, S-units are usually linked by ether bonds at their available 4-hydroxy position and eventually yield linear, chemically labile lignin polymers (Ozparpucu et al., 2018). In lignin, a proper S/G ratio is beneficial for application in various wood materials as well as for plant resilience (Li et al., 2016; Wang et al., 2018a; Wang et al., 2018b). Some recent studies have focused on the effects of altering the S/G lignin ratio using genetic engineering modification methods or oversuppression of COMT gene expression in plants such as *Arabidopsis* (Vanholme et al., 2010), wheat (Wang et al., 2018a; Wang et al., 2018b), *Miscanthus sinensis* (Yoo et al., 2018) and switchgrass (DeBruyn et al., 2017; Li et al., 2017).

The COMT gene was first cloned in aspen in 1991 (Li et al., 2015a); since then, several studies have identified COMT family genes and their expressional profiles in plants for their great potential in molecular breeding, especially among important forest timber species (Xu et al., 2009; Myburg et al., 2014; Shi et al., 2010; Carocha et al., 2015). The results have suggested that the COMT gene family comprises multiple members. For example, there are seven COMT genes in *Eucalyptus grandis* (*EgrCOMT*1, *EgrCOMT*2, *EgrCOMT*3, *EgrCOMT*4, *EgrCOMT*5, *EgrCOMT*6, and *EgrCOMT*7; Carocha et al., 2015), 25 in *Populus trichocarpa* (*PtrCOMT*1–25; Shi et al., 2010), and 14 and 42 in *Arabidopsis* and *Brassica napus*, respectively (Li et al., 2016). Within these species, differential COMT gene expression is observed among different tissues. For example, in *Eucalyptus grandis*, *EgrCOMT*1 was dramatically expressed in xylem compared to the other six COMTs, but exhibited low expression in the fruit capsule, flower buds, and shoot tips, whereas *EgrCOMT*2, 3, 4, and 5 were highly expressed in flowers and fruits, but showed low expression in xylem and phloem (Carocha et al., 2015). Specific spatiotemporal expression patterns may be due to their different functions.

*Catalpa bungei* belongs to the Bignoniaceae family and is a valuable timber forest tree native to China. Wood products of *C. bungei* exhibit excellent mechanical properties that make it ideal for construction and the manufacture of upmarket wooden products (Jing et al., 2015; Shi et al., 2017; Zheng et al., 2017). COMT genes are associated with wood mechanical properties (Li, Wu & Southerton, 2011; Liu et al., 2015; Wang et al., 2018a; Wang et al., 2018b); therefore, an understanding of the COMT gene family members and their expression profiles in *C. bungei* would lay a foundation toward future improvement of wood properties; however, this work has not yet been conducted. In this study, we identified possible members of the COMT gene family in *C. bungei* and investigated their evolutionary divergence and conserved domains. Previous studies have demonstrated that tension wood (TW) and opposite wood (OW) developed under tension or compression stress generally possess physical properties that differ from those of normal wood (NW), possibly due to differences in the composition and structure of polymers that comprised of the cell wall (Chen, Chen & Zhang, 2015). Tension wood usually forms on the upper side of the bent stems and was induced by gravistimulation and has been used as a model system for the study of carbon partitioning between lignin and cellulose in trees (Zinkgraf et al., 2018). Compared to NW, TW usually has less lignin, mannose and xylose, but more glucose and cellulose (Guedes et al., 2017). To select *CbuCOMT* genes associated with wood properties, the expression of 23 *CbuCOMT* genes in tension, normal, and opposite wood was detected by quantitative reverse-transcription polymerase chain reaction (qRT-PCR) analysis. The results of this study will provide a foundation for future functional studies of *CbuCOMT* genes.

## Materials and Methods

### *CbuCOMT* gene identification

To identify COMT homologue genes in *C. bungei*, amino acid sequences of the COMT conserved domain SAM_MT_COMT (PS51588) motifs (Li et al., 2016) and 14 *Arabidopsis* COMTs genes (Tair, https://www.arabidopsis.org (Li et al., 2016)) and 25 *Populus trichocarpa* COMTs (http://genome.jgi-psf.org/Poptr1_1/; Shi et al., 2010) were used as queries to perform a BLASTP search against the protein sequences of *C. bungei* data that annotated according to the *C. bungei* genome data(the entire *C. bungei* genome has been sequenced by our research group; the related paper is in preparation) using a cut-off *E* value of 1 × 10^−5^. We then reorganised and merged the highly matched sequences and used the InterProScan (http://www.ebi.ac.uk/Tools/pfa/iprscan5/) and SMART (http://smart.embl-heidelberg.de/) web tools to scan the protein domain for further verification of the selected putative *CbuCOMT* genes. COMT members from the genomes of *Zea mays* PH207 (Phytozome, https://phytozome.jgi.doe.gov/pz/portal.html), *Mimulus guttatus* (Phytozome, https://phytozome.jgi.doe.gov/pz/portal.html), *Solanum lycopersicum* (Phytozome, https://phytozome.jgi.doe.gov/pz/portal.html), *Solanum tuberosum* (Phytozome, https://phytozome.jgi.doe.gov/pz/portal.html), Utricularia gibba (CoGe, https://genomevolution.org/CoGe/), *Sesamum indicum* (Sinbase, http://ocri-genomics.org/Sinbase/), *Salvia miltiorrhiza* (National Data Center of Traditional Chinese Medicine of China, http://www.ndctcm.org/shujukujieshao/2015-04-23/27.html), *Capsicum baccatum* (Pepper Genome, http://peppergenome.snu.ac.kr/), and *Petunia axillaris* (Sol Genomics Network, https://solgenomics.net/organism/Petunia_axillaris/genome) were identified using the same methods.

Pairwise identity and similarity scores of the identified *CbuCOMT*s were calculated using the MatGAT v2.0 software. The theoretical isoelectric point (pI) and molecular weight (MW) of the COMT family genes in *C. bungei* were determined using the Expert Protein Analysis System (ExPASy, http://cn.expasy.org/).

### Plant materials

Leaves, current-growth stem (hereafter, stem), bark, developing xylem (hereafter, xylem), phloem, and flowers were sampled from three 8-year-old *C. bungei* clone “9-1” individuals on April 20, 2018, in a trial field located in Luoyang, China (112.55°N, 34.71°E). All plant samples were immediately frozen in liquid nitrogen and stored at −80 °C for later analysis.

We selected 1-year-old field-grown *C. bungei* clone “9-1” trees (height: 3–3.5 m, diameter at breast height (DBH): 2–2.5 cm) for the induction of TW and OW by bending and fixing the stems using nylon ropes to maintain the breast height (ca. 1.5 m) point of the stem at an angle of ca. 45°. Bending treatment was applied from April 19 to July 20 (the sample collection date) in 2017, during active cambial growth. Stem pieces were isolated at the bending point using lopping shears. TW (upper side) and OW (lower side) were collected from the same stem section using a sharp chisel after removing the bark, phloem, and cambium following the method of Li, Yang & Wu (2013). The breast height points of upstanding trees were selected to represent NW and sampled simultaneously. All samples were collected in the morning, cut to ca. 2 × 1 ×4 mm, and then immediately stored in liquid nitrogen.

### Sequence alignment, phylogenetic analyses and gene structure determination

Multiple sequence alignment was conducted using the DNAMAN software v. 6.0 (Lynnon Biosoft, Quebec, QC, Canada). Phylogenetic trees were constructed using the MEGA7.0 software. The phylogenetic trees were constructed using maximum likelihood (ML) method with the following parameters: and a bootstrap test with 1,000 replications, Poisson model, uniform rates, partial deletion of gaps and nearest neighbor interchange (Kumar, Stecher & Tamura, 2016). Full-length amino acid sequences of these genes were aligned using the ClustalW program under the default settings. We aligned the coding sequences to their corresponding genomic sequences to obtain the exon–intron structures of the COMT genes. A graph of the exon–intron structures was prepared using the online Gene Structure Display Server (GSDS, http://gsds.cbi.pku.edu.ch; Hu et al., 2015). The MEME web tool (http://meme-suite.org/) was used to search for motifs among all *CbuCOMT* genes. The number of motifs was set to eight (Kasirajan & Aruchamy, 2015).

### Analysis of regulatory elements in the promoter regions of *CbuCOMT* genes

The elements in the promoter fragments of the *CbuCOMT* genes (1,500 bp upstream of the translation initiation sites) were identified using the online program PlantCARE (http://bioinformatics.psb.ugent.be/webtools/plantcare/html/).

### RNA isolation and qRT-PCR

Total RNA was extracted using the RNeasy Plant Mini Kit (Tiangen) according to the manufacturer’s instructions. First-strand cDNA synthesis was performed using ca. 2 µg RNA using the PrimeScript II 1st Strand cDNA Synthesis Kit (TaKaRa, Kyoto, Japan) according to the manufacturer’s protocol. Gene-specific primers (Table S1) with melting temperatures of 58–62 °C and amplification lengths of 150–260 bp were designed using the Primer 5.0 software (Applied Biosystems, Life Technologies, New York, NY, USA). qRT-PCR was performed as follows: initial denaturation at 95 °C for 30 s, followed by 40 cycles of 5 s at 95 °C and 30 s at 60 °C, then one cycle of 5 s at 95 °C, 60 s at 60 °C, and a final stage at 95 °C (acquisition mode: continuous; five acquisitions per °C). RT-qPCR was performed using a Roche LightCycler 480 System (Roche, Basel, Switzerland) using the SYBR Premix Ex Taq Kit (TaKaRa) and an internal control (actin) primer pairs (Table S1) were selected (Jing et al., 2015). All reactions were conducted with four technical replicates and three biological replicates. Results obtained for different tissues were standardised to the levels of actin using the 2^−ΔΔ*CT*^ method. The data were statistically analysed by one-way ANOVA using SPSS 19.0 (SPSS Inc., Chicago, IL, USA). For the gene expression differences between OW, NW and TW, we employed a fold change not less than 2 as significant differential expressed genes.

## Results

### Genome-wide identification of the COMT gene family in *C. bungei*

To identify COMT genes in *C. bungei*, we performed a BLASTP search against the *C. bungei* protein database using COMT conserved domain sequences and amino acid sequences from *Arabidopsis* and *Populus trichocarpa*. After removing sequences lacking the functional domain using InterProScan and SMART, a total of 23 genes encoding putative COMT proteins were identified and named as *CbuCOMT*1 to *CbuCOMT*23 (Table 1).

**Table 1:** The *CbuCOMT*genes identified from the *Catalpa bungei*.

Gene name	Gene length (bp)	CDS length (bp)	Amino acids length (aa)	Theoretical Mw (kDa)	Theoretical PI
*CbuCOMT*1	2,117	1,083	360	40.66	5.54
*CbuCOMT*2	1,523	672	223	25.34	6.37
*CbuCOMT*3	3,700	1,107	368	40.26	5.47
*CbuCOMT*4	2,196	1,029	342	37.90	5.31
*CbuCOMT*5	3,797	1,482	493	54.14	6.28
*CbuCOMT*6	2,004	963	320	35.38	5.40
*CbuCOMT*7	1,132	579	192	21.57	7.12
*CbuCOMT*8	2,389	771	256	28.08	5.45
*CbuCOMT*9	5,736	918	305	33.79	6.00
*CbuCOMT*10	3,589	1,065	354	39.20	5.70
*CbuCOMT*11	3,977	1,062	353	39.00	5.47
*CbuCOMT*12	2,009	1,038	345	38.06	5.23
*CbuCOMT*13	1,915	1,038	345	37.70	5.27
*CbuCOMT*14	4,316	750	249	27.24	5.18
*CbuCOMT*15	1,410	1,059	352	38.62	6.27
*CbuCOMT*16	1,649	1,071	356	38.95	6.06
*CbuCOMT*17	1,918	1,038	345	38.17	5.27
*CbuCOMT*18	2,584	1,149	382	42.35	5.83
*CbuCOMT*19	1,790	897	298	33.28	6.20
*CbuCOMT*20	1,858	1,053	350	38.57	5.32
*CbuCOMT*21	2,957	969	322	35.53	5.50
*CbuCOMT*22	5,870	1,095	364	39.87	6.21
*CbuCOMT*23	1,587	978	325	35.71	5.23

### Sequence features and sequence similarities among *CbuCOMT*s

**Figure 1:**
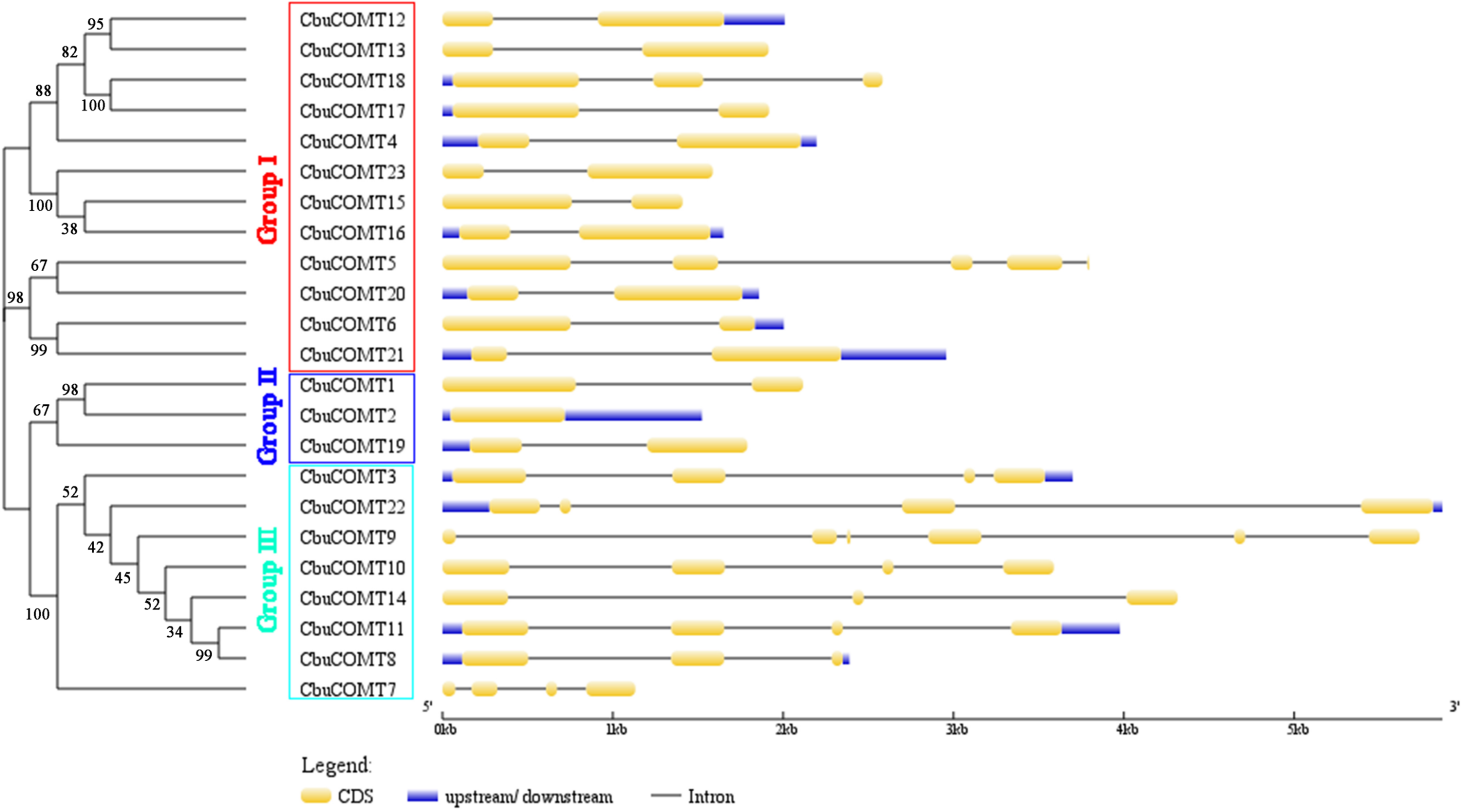
Phylogenetic analysis of CbuCOMT proteins and exon-intron structure of the corresponding genes. Exons and introns are respectively indicated by yellow boxes and black lines connecting two exons and the numbers on the branches were Bootstrap values.

Sequence feature analysis of COMT genes suggested that *CbuCOMT* gene lengths varied from 1,132 bp (*CbuCOMT*7) to 5,870 bp (*CbuCOMT*22) and the lengths of open reading frames (ORFs) ranged from 579 bp (*CbuCOMT*7) to 1,482 bp (*CbuCOMT*22), with deduced amino acid sequence lengths varying from 193aa to 493aa (Table 1). Phylogenetic analysis of the 23 *CbuCOMT*s indicated that the *CbuCOMT* genes could be classified into three groups: group I was composed of 12 CbuCOMT proteins; *CbuCOMT*1, *CbuCOMT*2, and *CbuCOMT*19 belonged to an independent branch, group II; the remaining *CbuCOMT* genes comprised group III (Fig. 1). A matrix of amino acid sequence similarity for the *CbuCOMT* gene family is presented in File S1. A high percentage of amino acid identity and similarity was observed between *CbuCOMT*5 and *CbuCOMT*21 (91.3 and 94.9%, respectively), *CbuCOMT*14 and *CbuCOMT*22 (88.4 and 92.8%, respectively), *CbuCOMT*4 and *CbuCOMT*15 (94.1 and 97.2%, respectively), and between *CbuCOMT*18 and *CbuCOMT*19 (83.6 and 92.1%, respectively). However, most *CbuCOMT* pairs showed low amino acid sequence identity and similarity (<80%).

### Phylogenetic analysis of *CbuCOMT*s

**Figure 2:**
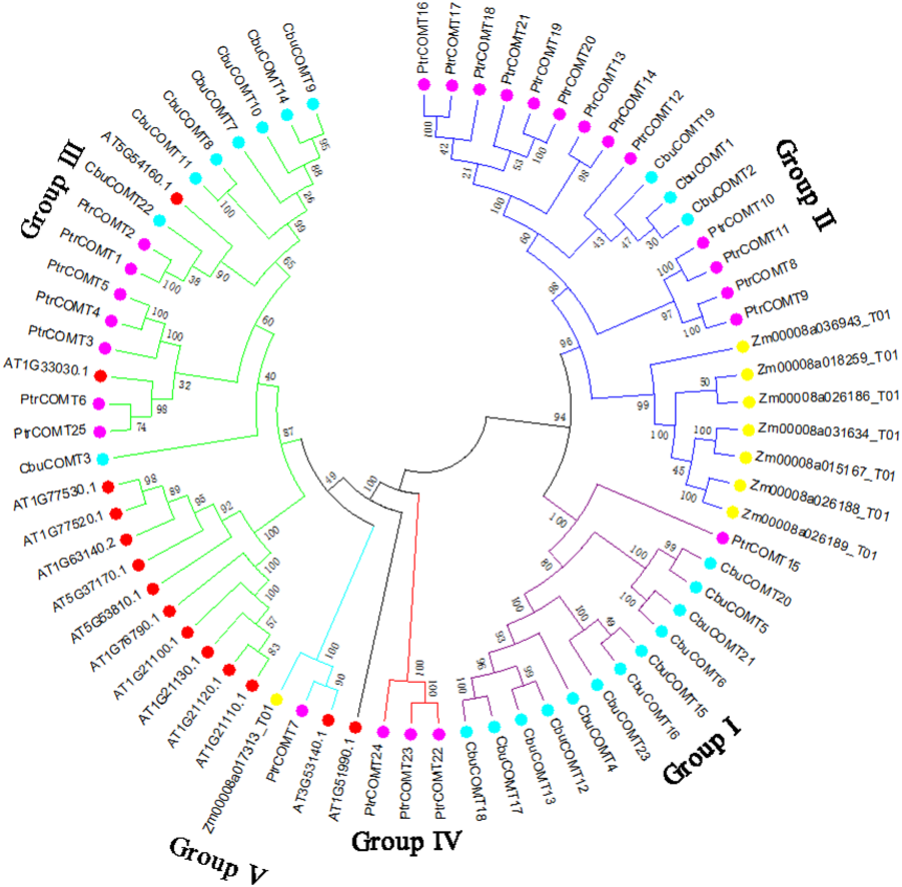
Phylogenetic analysis of CbuCOMT and COMT genes from Arabidopsis thaliana, Populus trichocarpa and Zea mays by MEGA 7.0. The 5 groups were distinguished in different colors and the numbers on the branches were Bootstrap values.

To investigate the evolutionary relationships among COMT proteins, we first generated a phylogenetic tree using full-length protein sequences of the 23 *CbuCOMT*s, 25 *P. trichocarpa* COMTs (*PtCOMT* s), 8 *Z. mays* COMTs (*ZmCOMT*s) and 14 *A. thaliana* COMTs (*AtCOMT* s). As shown in Fig. 2, the COMTs of these three species were distinctly classified into five groups (I, II, III, IV, and V). The *CbuCOMT*s were mainly distributed in groups I and III, whereas only three *CbuCOMT*s belonged to group II (*CbuCOMT*1, *CbuCOMT*2, and *CbuCOMT*19), in accordance with the previous phylogenetic tree (Fig. 1). The *CbuCOMT*s in group III were orthologs of At5G54160.1, whereas *CbuCOMT*s in group I were possible orthologs of *PtrCOMT*15.

**Figure 3:**
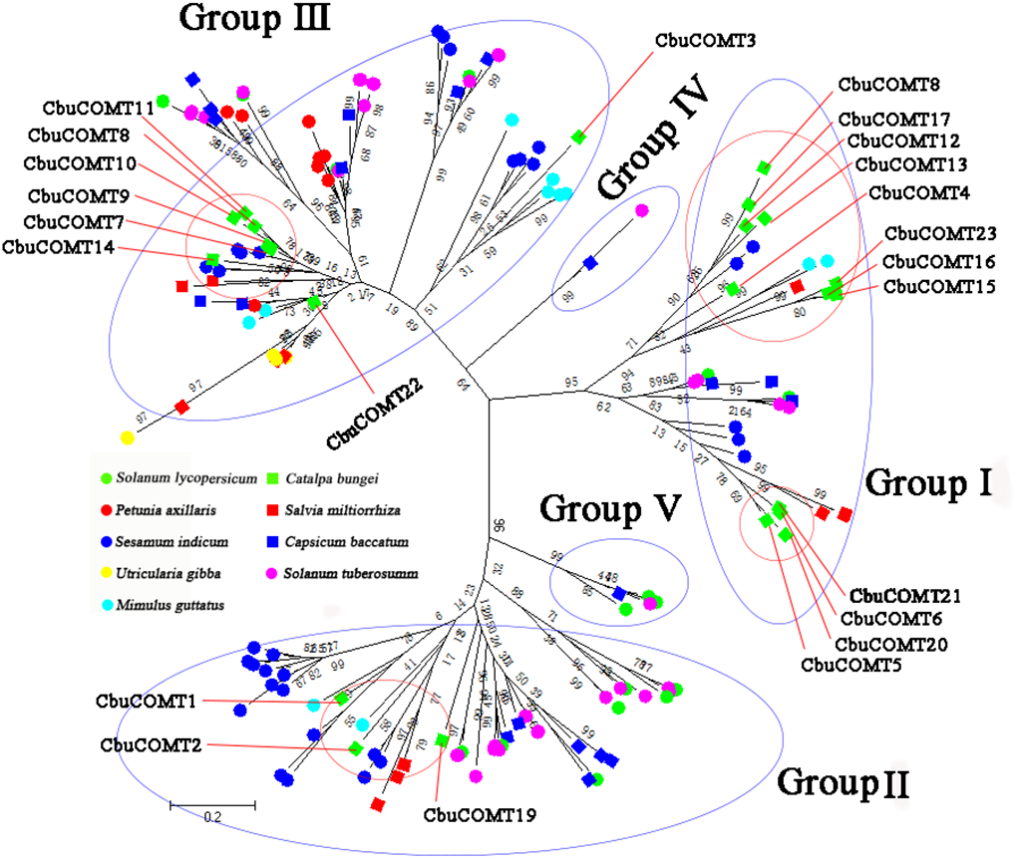
Phylogenetic analysis of CbuCOMTs and other COMT gene family members. The numbers in the brackets indicated the number of genes we used to construct the evolutionary tree and the numbers on the branches were Bootstrap values.

We then constructed another phylogenetic tree using 164 possible COMT proteins from *C. bungei* and eight other Tubiflorae plants (Table S2) including *C. bungei* (23), *Mimulus guttatus* (11), *Solanum lycopersicum* (17), *Solanum tuberosum* (34), *Utricularia gibba* (10), *Sesamum indicum* (32), *Salvia miltiorrhiza* (10), *Capsicum baccatum* (19) and *Petunia axillaris* (8). The results indicated a grouping into five sub-families (Fig. 3) and provided information about the evolution of the *CbuCOMT* gene family. *CbuCOMT* s belonged to groups I, II, and III. The clustering results for *CbuCOMT* proteins in Tubiflorae plants were the same as those for *AtCOMT* and *PtCOMT* proteins, with *CbuCOMT*4, 5, 6, 12, 13, 15, 16, 17, 18, 20, 21 and 23clustered in group I, *CbuCOMT*1, 2, and 19 clustered in group II, and *CbuCOMT*3, 7, 8, 9, 10, 11, 14 and 22 clustered in group III. The phylogenetic tree showed that *CbuCOMT* s were distributed most closely to COMTs from *Sesamum indicum* and *Salvia miltiorrhiza*, suggesting that *C. bungei*, *Sesamum indicum*, and *Salvia miltiorrhiza* have a close genetic relationship.

### Sequence alignment and structural analysis of *CbuCOMT* genes

**Figure 4:**
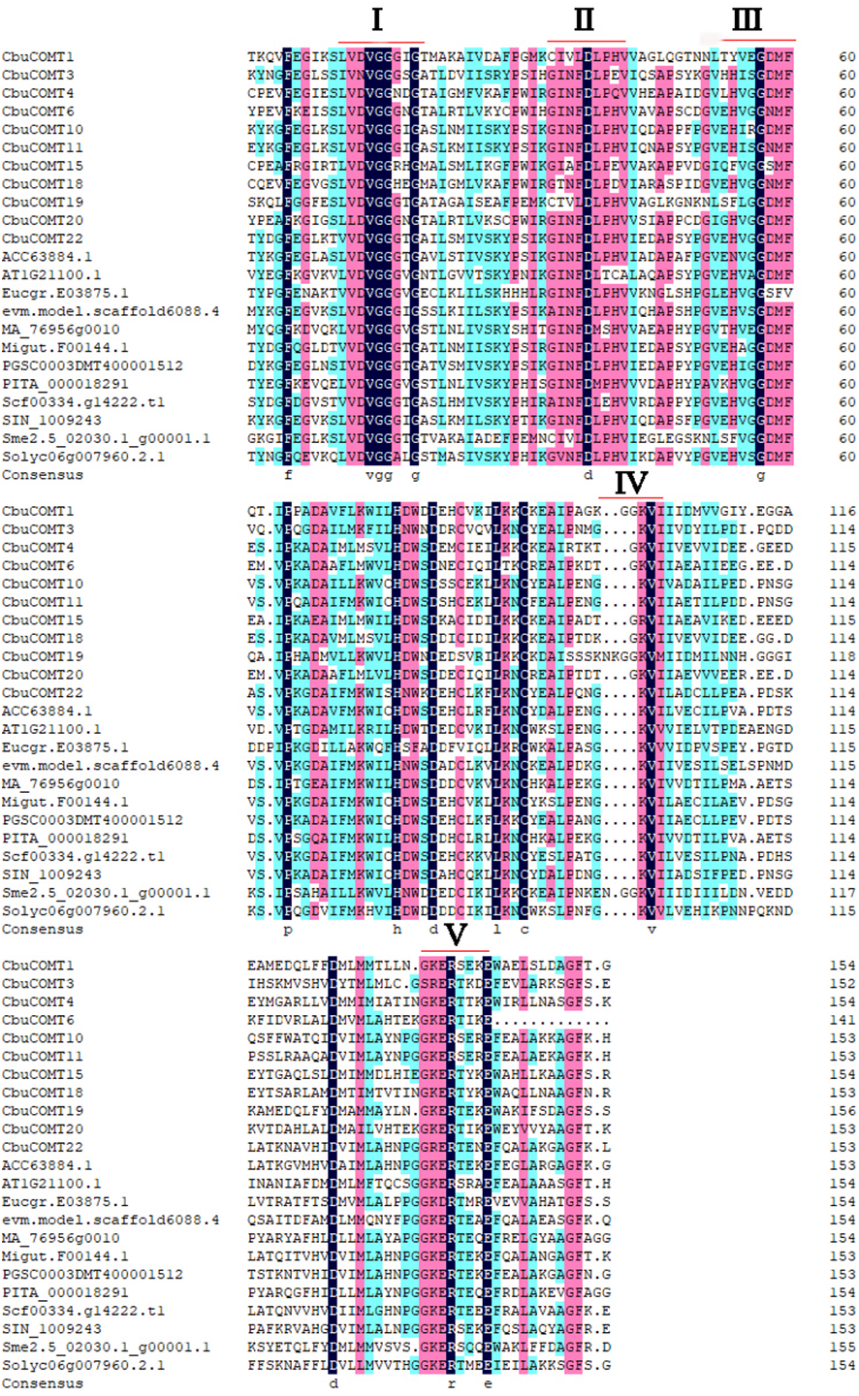
Multiple sequence alignment of the conserved domains of the COMT proteins. Five conserved sequences: I: LVDVGGGxG, II: GINFDLPHV, III: EHVGGDMF, IV: NGKVI and V: GGKERT were marked by black lines

The deduced amino acid sequences of 11 *CbuCOMT*s (*CbuCOMT*1, 3, 4, 6, 10, 11, 15, 18, 19, 20 and 22) were randomly selected (make sure at least one *CbuCOMT* protein form the three groups were included), together with12 other COMTs from *A. thaliana* (AT1G211001.1), *Salvia miltiorrhiza* (EVM.MODEL.SCAFFOLD6088.4), *Utricularia gibba* (SCF00334.G14222.T1), *Pinus taeda* (PITA_000018291), *Sesamum indicum* (SIN_1009243), *Solanum tuberosum* (PGSC0003DMT400001512), *Populus trichocarpa* (ACC63884.1), *Picea abies* (MA_76956G0010), *Eucalyptus grandis* (EUCGR.E03875.1), *Mimulus guttatus* (MIGUT.F00144.1), and *Solanum melongena* (SME2.5_02030.1_G00001.1) were aligned to determine COMT structures (Fig. 3). Through a comparison of COMT amino acid sequences from different plant species, Ibrahim, Bruneau & Bantignies (1998) found that COMT proteins had five conserved sequences: I (LVDVGGGxG), II (GINFDLPHV), III (EHVGGDMF), IV (NGKVI), and V (GGKERT) (Ibrahim, Bruneau & Bantignies, 1998). Very similar results were obtained in our study; however, some amino acid variations were observed in the conserved domain; for example, conserved sequence I in *CbuCOMT*3 was IVNVGGGxG. Conserved sequences IV and V also exhibited amino acid variation (Fig. 4).

To gain further insight into the structural diversity of *CbuCOMT* genes, full-length cDNA sequences were compared with the corresponding genomic DNA sequences to determine the numbers and positions of exons and introns within the genomic DNA (Fig. 1). The number of introns fluctuated markedly from zero to five. *CbuCOMT*2 contained no introns, whereas *CbuCOMT*9 had five, indicating that gene structure may not be conserved among *CbuCOMT* family members (Fig. 1).

### Motifs and cis-regulatory elements in the promoter regions of *CbuCOMT*

**Figure 5:**
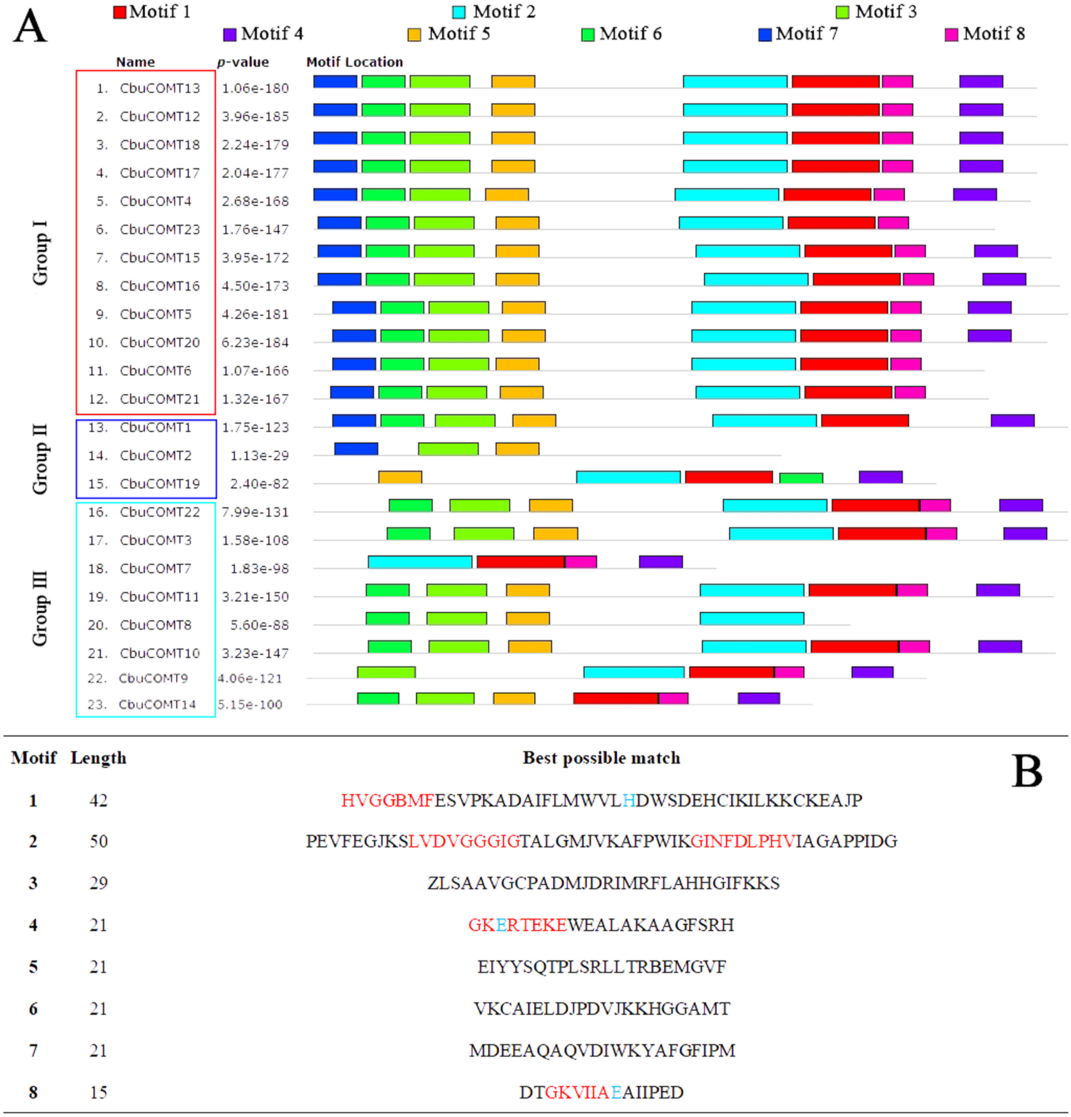
Distribution of motifs in the COMT proteins. (A) The motifs were identified by MEME. Different motifs are indicated by different colored numbers 1–8. (B) The detail motif sequences. Five conserved domain sequences were marked by red color, and catalytic residues were marked by arrows.

After performing a search using the MEME motif search tool, eight consensus motifs were detected among the *CbuCOMT*s (Fig. 5). Most *CbuCOMT*s possessed motifs 1, 2, and 3. Notably, all five conserved domains were totally or partly contained within the five motifs; for example, conserved domain I (LVDVGGGxG), which is an S-adenocyl-L-methionine (SAM) binding domain, was included in motif 2 and found in many COMT genes. In addition, three COMT-specific catalytic residues were found in motifs 1 (histidine, H), 4 (glutamic acid, E), and 8 (glutamic acid, E) (Li et al., 2015b).

**Table 2:** A list of motifs detected in the promoter regions of *CbuCOMT*genes.

Gene ID	Motifs related to growth and development	Motifs related to light response	Motifs related to stress response	Motifs related to hormone response
*CbuCOMT*1	GCN4_motif, Skn-1_motif, circadian	Box 4, Box I, G-Box, GT1-motif, Sp1, Box 2, as-2-box, TCT-motif	ARE, BOX-W1, MBS, TC-rich repeats	ABRE, TCA-element
*CbuCOMT*2	O_2_-site	AT1-motif, ATCT-motif, Box 4, Box 1, CATT-motif, G-Box, GA-motif, GT1-motif, MRE, TCT-motif	ARE, TC-rich repeats	ABRE
*CbuCOMT*3	Skn-1_motif	BOX 4, BOX 1, G-BOX, CATT-motif, GATA-motif, GT1-motif	ARE, TC-rich repeats	ABRE, TATC-box, TGA-box
*CbuCOMT*4	CAT-box, Skn-1_motif, circadian	Box 4, Box 1, ATCC-motif, ATCT-motif, CATT-motif, G-Box, GAG-motif, I-box, chs-CMA2a	ARE, HSE	ABRE, TCA-element
*CbuCOMT*5	Circadian, GCN4_motif, Skn-1_motif	ACE, BOX 4, BOX 1, G-BOX, GA-motif, G1-motif, Sp1, TCT- motif, BOX 2, chs-CMA1a, chs-CMA2a	ARE, BOX-W1, HSE, MBS, TC-rich repeats	ERE
*CbuCOMT*6	CAT-box, GC-motif, GCN4_motif, O_2_-site, Skn-1_motif, circadian	AT1-motif, ATCC-motif, Box 4, Box 1, G-Box, GATA-motif, GT1-motif, Gap-box, I-box, TCT-motif	HSE, MBS, TC-rich repeats	ABRE, ERE, GARE-motif, P-box, TGA-box
*CbuCOMT*7	Circadian, CAT-BOX, GCN4_motif, Skn-1_motif, O2-site, GC-motif	BOX 1, BOX4, G-BOX, I-box, GATA-motif, ACE, AE-box, CATT-motif, GT1-motif, L-box, TGG-motif, as-2-box	HSE, MBS	ABRE, GARE-motif, TCA-element, ERE
*CbuCOMT*8	Skn-1_motif	BOX 1, I-box, G-BOX, BOX4, ACA-motif, GATA-motif, TCT-motif	ARE, TC-rich repeats, BOX-W1, HSE	
*CbuCOMT*9	MSA-like, O_2_-site	TCT- motif, BOX I, G-BOX, chs-CMA1a, AAAC-motif, ATCT-motif, MRE, TCT-motif	ARE, MBS	ABRE, GARE-motif, TGA-box
*CbuCOMT*10	Skn-1_motif	BOX 1, G-BOX, ATCT-motif, I-box, GT1-motif, LAMP-element	ARE, BOX-W1, HSE, LTR, MBS	GARE-motif, TGA-box
*CbuCOMT*11	Skn-1_motif	BOX 4, BOX 1, G-BOX, GATA-motif, TCT- motif, 3-AF1 binding site, ACA-motif, I-box	ARE, BOX-W1, HSE, TC-rich repeats	
*CbuCOMT*12	circadian, Skn-1_motif	Box 4, Box 1, Box 2, GAG-motif, GT1-motif, G-Box, MNF1	ARE, TC-rich repeats, HSE, MBS	ABRE, ERE, P-box
*CbuCOMT*13	CAT-box, GCN4_motif, O2-site, Skn-1_motif, circadian	ACE, Box 4, G-Box, GATA-motif, CATT-motif, I-box, LAMP-element, Sp1, MNF1, TCT-motif	HSE, MBS, TC-rich repeats	ABRE, TATC-box, TCA-element,
*CbuCOMT*14	O_2_-site, Skn-1_motif	Box 4, Box 1, GT1-motif, ATC-motif, GAG-motif, GT1-motif, I-box, Sp1, as-2-box	HSE, BOX-W1, MRE	ERE, TCA-element
*CbuCOMT*15	O_2_-site, Skn-1_motif, circadian	ACE, ATCT-motif, Box 4, Box 1, G-Box, GA-motif, GT1-motif, I-box, Sp1, TCT-motif, chs-CMA2a, BOX 2	HSE, TC-rich repeats	ABRE, TCA-element
*CbuCOMT*16	Circadian, GCN4_motif	Box 4, BOX-W1	LTR	TCA-element, GARE-motif
*CbuCOMT*17	Circadian, Skn-1_motif	ATCT-motif, Box 4, Box 1, BOX-W1, G-Box, GAG-motif, GT1-motif, chs-CMA2a, Gap-box, MNF1	ARE, MBS, HSE, P-Box	TGA-box, ABRE, TCA-element
*CbuCOMT*18	Circadian, Skn-1_motif	ATCT-motif, Box 4, Box 1, GAG-motif, GT1-motif, chs-CMA2a, MRE, Sp1, as-2-box, chs-Unit 1 ml, GATA-motif	ARE, LTR, MBS, TC-rich repeats	TGA-box, ABRE, ERE, TCA-element, P-Box
*CbuCOMT*19	Skn-1_motif, circadian	Box 4, Box 1, CATT-motif, GAG-motif, GT1-motif	MBS, MBSII, TC-rich repeatst	TCA-element
*CbuCOMT*20	GC-motif, GCN4_motif, Skn-1_motif	G-Box, CATT-motif, I-box, GT1-motif, ACE	ARE, BOX-W1, HSE, TC-rich repeats	
*CbuCOMT*21	O_2_-site, Skn-1_motif, circadian	Box 4, Box 1, G-Box, I-box, Sp1, CATT-motif, as-2-box, MRE	ARE, TC-rich repeats	ABRE, TATC-box, TGA-box
*CbuCOMT*22	CAT-box, GCN4_motif, O_2_-site, Skn-1_motif,	ATCT-motif, Box 4, Box 1, CATT-motif, G-Box, LAMP-element, MNF1, TCT-motif	ARE, BOX-W1, HSE, MBS, TC-rich repeats	ERE
*CbuCOMT*23	Skn-1_motif, circadian	Box 4, Box 1, GT1-motif, I-box, MNF1, as-2-box	ARE, BOX-W1	TCA-element, TGA-box

To identify the likely cis-regulatory elements (CREs) of *CbuCOMT*s, the promoter regions of the *CbuCOMT* genes were used to search the PlantCARE database; the results are listed in Table 2. A series of CREs involved in developmental processes such as circadian motifs and the Skn-1 and GCN4 motifs found in the promoters of the *CbuCOMT*14, 20, and 8 genes. Additionally, Box I, Box 4, the ATCT motif and other light response-related motifs were also found among the promoters of *CbuCOMT* genes, indicating that the expression of *CbuCOMT* genes may be regulated by light. Notably, several hormone response motifs were also identified. For example, ABRE (abscisic acid [ABA]-responsive element), GARE-motif, P-box, and TATC-box (gibberellic acid-responsive elements), ERE (ethylene-responsive element), and TCA (salicylic acid-responsive element), and TGA (auxin-responsive element) were found among the promoters. In addition, several CREs related to abiotic stress responses were found in the promoters of *CbuCOMT* genes; for example, HSE was present in the promoters of 14 *CbuCOMT* genes. The anaerobic induction element (ARE), defence and stress-responsive element (TC rich), and MYB binding sites involved in drought inducibility (MBS) were found in 15, 14, and 13 *CbuCOMT* gene promoters, respectively.

### Expression profiles of *CbuCOMT* genes in various tissues

To identify the expression patterns of *CbuCOMT*s, qRT-PCR was performed on the 23 *CbuCOMT*s in six different tissues, the leaves, bark, xylem, phloem, branches and flowers of 8-year-old *C. bungei* plants. To verify the specificity of each primer for qRT-PCR, the primers were checked using BLAST against the genome sequence of *C. bungei*, and qRT-PCR products were sequenced. The qRT-PCR results are shown in File S2. COMT genes exhibited markedly different expressional profiles; for example, *CbuCOMT*10, and 23 were highly expressed in xylem, but low expression was observed among the other five tissues, whereas *CbuCOMT*17 and 18 were more highly expressed in leaves, young stems, and phloem, but showed very little expression in flowers and bark. Among the 23 *CbuCOMT*s, *CbuCOMT*1, 3, 6, 7, 8, 13, 14, 16, 19 showed extremely low expression in all six tissues, possibly due to spatially or temporally specific expression. Expression levels of *CbuCOMT*1 and 20 were much higher in flowers than in other tissues.

### Expression profiles of *CbuCOMT*s in TW, NW, and OW

The relative expression levels of the 23 *CbuCOMT* genes in TW, NW, and OW are shown in Table 3. The qRT-PCR results showed that *CbuCOMT*3, 6, 13, 16, 19, and 22 were not expressed in xylem (TW, NW, and OW), and *CbuCOMT*11 was not expressed in TW. Although the expression levels of most *CbuCOMT* genes changed following the bending treatment, only *CbuCOMT*10 and 23 showed significant differences (—log_2_FC— ≤1). The expression levels of *CbuCOMT*10 and 23 decreased significantly in TW compared to NW (log_2_FC (TW/NW) = −1.22 and −1.27, respectively); however, the expression levels of these two genes showed an opposite trend in OW such that *CbuCOMT*23 increased markedly in xylem under compression stress (log_2_FC = 1.35), whereas *CbuCOMT*10 showed a downward trend in expression (log_2_FC = −0.43).

**Table 3:** *CbuCOMT*genes expression profiles in TW, NW and OW (mean value ± standard deviation).

ID	TW	NW	OW	log_2_FC (TW/NW)	log_2_FC (OW/NW)
*CbuCOMT*1	2.22 ± 0.56	2.37 ± 0.94	2.59 ± 0.27	−0.15	0.22
*CbuCOMT*2	2.69 ± 0.19	3.48 ± 0.28	3.38 ± 0.22	−0.79	−0.10
*CbuCOMT*3	–	–	–	–	–
*CbuCOMT*4	1.06 ± 0.05	1.78 ± 0.13	1.77 ± 0.38	−0.72	−0.01
*CbuCOMT*5	3.22 ± 0.64	3.01 ± 0.47	2.85 ± 0.59	0.21	−0.16
*CbuCOMT*6	–	–	–	–	–
*CbuCOMT*7	0.77 ± 0.23	0.80 ± 0.15	1.36 ± 0.56	−0.03	0.56
*CbuCOMT*8	0.88 ± 0.12	0.84 ± 0.22	0.78 ± 0.11	0.04	−0.08
*CbuCOMT*9	2.20 ± 0.32	3.01 ± 0.26	3.50 ± 0.45	−0.81	0.49
*CbuCOMT*10	3.65 ± 1.01	4.87 ± 1.20	4.44 ± 0.88	−1.22	−0.43
*CbuCOMT*11	–	0.11 ± 0.01	0.10 ± 0.02	–	−0.01
*CbuCOMT*12	2.08 ± 0.25	2.31 ± 0.11	2.58 ± 0.36	−0.23	0.27
*CbuCOMT*13	–	–	–	–	–
*CbuCOMT*14	0.58 ± 0.06	0.44 ± 0.04	1.16 ± 0.18	0.14	0.72
*CbuCOMT*15	0.66 ± 0.08	0.98 ± 0.12	0.59 ± 0.09	−0.32	−0.39
*CbuCOMT*16	–	–	–	–	–
*CbuCOMT*17	0.24 ± 0.02	0.24 ± 0.01	0.54 ± 0.13	0	0.30
*CbuCOMT*18	5.25 ± 1.11	5.21 ± 1.32	4.26 ± 1.54	0.04	−0.95
*CbuCOMT*19	–	–	–	–	–
*CbuCOMT*20	0.41 ± 0.05	0.30 ± 0.02	0.64 ± 0.07	0.11	0.34
*CbuCOMT*21	3.03 ± 0.76	3.24 ± 0.68	4.12 ± 1.09	−0.21	0.88
*CbuCOMT*22	–	–	–	–	–
*CbuCOMT*23	9.96 ± 0.88	11.23 ± 1.25	12.58 ± 1.58	−1.27	1.35

**Notes.**

– means no expression.

## Discussion

COMT family genes are ubiquitous among plant species and have been surveyed at the whole-genome level in several species including *A. thaliana*, *Brassica napus*, *Brachypodium distachyon*, *Oryza sativa*, *Populus trichocarpa ,* and others. In this study, a total of 23 COMT genes were identified based on the *Catalpa bungei* genome and named as *CbuCOMT*1 to *CbuCOMT* 23. Genomic DNA, CDS lengths, and deduced amino acid sequences were variable among these genes (Table 1), which led to changes in the theoretical MW and pI of *CbuCOMT* members. The structure of the phylogenetic tree obtained from the alignment of *AtCOMT* s, *PtCOMT* s, and *CbuCOMT*s indicates that COMT genes can be divided into five major groups, as reported in a previous study (Li et al., 2016). The functions of COMTs have been identified, and they play vital roles in S-type lignin production; however, to date, their roles in *C. bungi* have remained unclear. Therefore, we preliminarily predicted the functions of *CbuCOMT*s based on the homology of other COMTs. According to the phylogenetic tree, AT5G54160.1 belonged to group III and was closely related to *CbuCOMT*3, 7, 8, 9, 10, 11, 14, and 22, indicating that *CbuCOMT*s belonging to group III may have similar functions. AT5G54160.1 encodes a caffeic acid O-methyltransferase that participates in S-lignin synthesis (Moinuddin et al., 2010; Vanholme et al., 2010) and melatonin synthesis by catalysing N-acetylserotonin methylation in *Arabidopsis* ((Byeon et al., 2014)). It has been reported that some COMT genes are involved in lignin biosynthesis and other processes such as glucosinolate metabolism. At1g21100, At1g21110, At1g21120, At1g21130, and At1g76790 mainly participate in glucosinolate metabolism and other processes (Li et al., 2016). These genes clustered together with *CbuCOMT*s in group III, indicating that *CbuCOMT*s in this group may also participate in glucosinolate metabolism. Except for *CbuCOMT*3, *CbuCOMT*s in group III also clustered with *PtrCOMT*2, of which protein abundance and COMT total activity gradually and continuously increased during the lignification of *Populus* early stems, indicating that *PtrCOMT* 2 may participate in the lignification of *Populus* stems (Liu et al., 2015). In our study, *CbuCOMT*7, 9, and 10 from group III were more highly expressed in xylem than in the other five organs, suggesting that they may be involved in the lignification of xylem in *C. bungei*.

*CbuCOMT*s in groups I and II clustered with *PtrCOMT* s. The downregulation of COMT expression in poplar has been explored by several researchers. Jouanin et al. (2000) found a COMT downregulated transgenic poplar line with almost no COMT activity. In this line, the lignin level was reduced by 17% and lignin structure was strongly altered, with a 200% increase in condensed bond content and a nearly complete lack of S-lignin. In *Populus trichocarpa*, 25 COMT genes were identified by Shi et al. (2010); the expression profiles of 25 *PtrCOMT* differed significantly from each other. *PtrCOMT* 2 was highly expressed in leaves, shoots, phloem, and xylem, whereas *PtrCOMT* 19 had only slight or no expression in these organs, indicating that, although these genes belong to the same family, they may have different functions . This finding is also supported by a study of the AT4G35160.1 gene, which was identified as a COMT gene based on functional domain analysis, but was later shown to mainly participate in melatonin synthesis with very little caffeic acid O-methyltransferase activity in *Arabidopsis* (Lee et al., 2014). Notably, *CbuCOMT*23 expression was dramatically higher than that of the other 22 *CbuCOMT*s in NW (xylem), indicating that *CbuCOMT*23 may have important functions in xylem. TW usually contains more cellulose and less hemicellulose and lignin than does NW, whereas OW has greater lignin content. Thus, many researches have conducted microarray analysis of artificially bent trunks to identify genes that participate in the synthesis of lignin, phytohormone, cellulose, and many other components (Chen, Chen & Zhang, 2015). In our study, *CbuCOMT*23 expression decreased under tension stress (TW) and increased in OW, implying that *CbuCOMT*23 gene may be involved in lignin synthesis in xylem, and associated with *C. bungei* wood properties. The expression of *CbuCOMT*10 showed significantly reduce in TW, suggesting *CbuCOMT*10 may be involved in the declined lignification of TW. However, these hypotheses must be verified through further experiments. In addition, the expression of genes were normalized by a reference gene, not absolute quantification, which is just a relative quantification. In later study, we will further quantify the COMT genes using RNA-seq or absolute quantification.

Fewer COMTs were identified in *C. bungei* (23, genome size: 740 M) than in *Sesamum indicum* (34, genome size: 274 M), *P. trichocarpa* (25, genome size: 480 M), and *Solanum tuberosum* (34, genome size: 844 M), but more than in *A. thaliana* (14, genome size: 125 M), *Mimulus guttatus* (11, genome size: 430 M), *Solanum lycopersicum* (17, genome size: 900 M), *Utricularia gibba* (10, genome size: 82 M), *Salvia miltiorrhiza* (10, genome size: 641 M), *Petunia axillaris* (8, genome size: 1.4 Gb), and *Capsicum baccatum* (19, genome size: 3.48 Gb). The number of COMTs did not increase with the enlargement of the genomes, possibly due to the unusual expansion and contraction history of the COMT gene family in these species. The phylogenetic tree indicated that *C. bungei* may have a closer genetic evolutionary relationship with *Sesamum indicum* than with the other plants investigated in this study; this result was similar to those of our studies on other genes (unpublished data, the relevant paper is under preparation now).

In our study, most *CbuCOMT*s had one to three introns, and within the same phylogenetic group, members generally had similar exon–intron structures. For example, *CbuCOMT*s in group II had one intron or none, whereas *CbuCOMT*s in group III had two or more introns, indicating that the evolution of the COMT gene family may be closely related to the diversification of gene structures. Similar results have been obtained in other gene families (Song et al., 2017). It has been reported that genes with lower intron densities are rapidly expressed after induction because introns may affect expression efficiency through at least three possible mechanisms, delaying transcript production by (1) splicing, (2) additional length of the nascent transcript, or (3) increased energetic cost due to increased transcript length (Jeffares, Penkett & Bähler, 2008). In our study, most *CbuCOMT*s clustered in groups I and II had fewer introns than *CbuCOMT*s from group III, suggesting a possible faster response to induction, however, it still needs to be further demonstrated.

The regulation of gene transcription is a complex process involving various proteins bound in a sequence-specific manner to cis-regulatory elements present in the promoter regions. In our study, numerous cis-regulatory elements related to light response were found; among these, GATA-motifs (Argüello-Astorga & Herrera-Estrella, 2018), I-box (Donald & Cashmore, 1990), GT1-motif (Gao et al., 2004), and G-box (Giuliano et al., 1988) are essential for light-mediated transcriptional activity. The results of our study suggest that *CbuCOMT* genes may regulate S-type lignin synthesis by interacting with light-inducible proteins and that 14 *CbuCOMT* gene promoters also contained motifs for circadian cycles. In higher plants, the expression of a large number of genes is under circadian regulation, including genes associated with photosynthesis, starch mobilising enzymes, and some metabolic pathways. Hormones are key regulators of plant growth and development. ABA-, auxin-, ethylene-, GA-, and salicylic acid-responsive elements were found in the promoters of *CbuCOMT* genes, indicating a potential role for hormones in the regulation of *CbuCOMT* genes, in agreement with Kim et al. (2013), who demonstrated that the expression of a COMT gene from kenaf (*Hibiscus cannabinus*) increased after 6 h of treatment with salicylic acid. They also found that the COMT gene could be induced by cold, H_2_O_2_, and salt (Kim et al., 2013), which indicated that the transcription of COMT genes may be induced by both hormones and some abiotic stresses. Li et al. (2016) found that some COMT family genes in *Brassica napus* had higher expression levels under drought treatment than under non-stressed conditions (Li et al., 2016). Similarly, Li et al. (2015a) demonstrated that the expression of a COMT gene in *Ligusticum* could be significantly induced by cold and drought, but not salt (Li et al., 2015a). In our study, motifs related to stress, such as ARE, LTR, and MBS were present in some COMT promoter sequences, indicating that these COMT genes may responsible for sensing environmental stresses. Heat-responsive cis-acting regulatory elements were found in some *CbuCOMT* gene promoters, suggesting their possible roles in heat stress response and MBS-conferred drought response in plants (Asif et al., 2014; Table 2). Other CREs such as Box-W1, TC-rich repeats, ARE, and LTR are also involved in stress response (Zhang et al., 2015). Our results indicate that *CbuCOMT* gene expression may be induced by abiotic stresses; however, this finding must be further studied.

## Conclusion

A relatively complete basic analysis of COMT gene family members in *C. bungei* was performed in this study. We identified 23 genes as putative *CbuCOMT* genes and their expression levels in six different *C. bungei* tissue types were assessed using qRT-PCR. Distinctly different expression profiles among members of the *CbuCOMT* gene family suggest that these genes may play different roles in development. Our results provide a foundation for elucidating the functions of *CbuCOMT* family genes; however, further study of each family member using genetic modification is essential to resolve their specific functions.

##  Supplemental Information

10.7717/peerj.6520/supp-1 Table S1 Quantitative real-time PCR primers for *CbuCOMT*genes

10.7717/peerj.6520/supp-2 Table S2 The information of possible COMT genes in other Tubiflorae plants

10.7717/peerj.6520/supp-3 File S1 Identity and similarity of 23 CbuCOMTs

10.7717/peerj.6520/supp-4 File S2 Expression levels of the members of the CbuCOMT gene family in different organs detected by qRT-PCR Data with the same letter are not significantly different ( *P* = 0.05) according to Duncan’s multiple range test. The error bar refers to standard deviation.

10.7717/peerj.6520/supp-5 File S3 The amino acid sequences of 23 CbuCOMTs

10.7717/peerj.6520/supp-6 File S4 ORF sequences of 23 CbuCOMTs

10.7717/peerj.6520/supp-7 File S5 CDS sequences of 23 CbuCOMTs

10.7717/peerj.6520/supp-8 File S6 The sequences of 23CbuCOMT promoters

## Additional Information and Declarations

### Data Availability

The following information was supplied regarding data availability: The sequences of 23 CbuCOMTs are available at GenBank, accession numbers: MH837487 to MH837509. The sequences can be also found in File S2, S3 and S4.
