# Supplementary material for: Genome-wide analysis of the Catalpa bungei caffeic acid O-methyltransferase (COMT) gene family: identification and expression profiles in normal, tension, and opposite wood
Source: PeerJ. 2019 Mar 14;7:e6520. doi: 10.7717/peerj.6520 (PMC6421059; doi:10.7717/peerj.6520)
Supplement: Table S1 [file peerj-07-6520-s001.docx]

Table S1. Quantitative real-time PCR primers for *CbuCOMT* genes

| Gene name | Froward primer (5’-3’) | | Reverse primer (5’-3’) | |
| --- | --- | --- | --- | --- |
| *CbuCOMT*1 | | ATCACCACCTCTCACCCTTCAAGAT | | GCCACCACCTACATCCACTAATGAC |
| *CbuCOMT*2 | | GAGTCTTCCTCATCATTCGTCACCA | | GCCACCTCCAACATCCACCATTG |
| *CbuCOMT*3 | | CGCCCGCCTAATTCCAATTTCCA | | GACTCGCAAGCAGTCGAAGCAA |
| *CbuCOMT*4 | | CTGCGGCTGATGAGATGACGATG | | GTCGTTACCACCAACATCCACCAA |
| *CbuCOMT*5 | | TTGGTGGATGTTGGTGGTGGTAATG | | CACACGGTGGAGCGATGGAAAC |
| *CbuCOMT*6 | | CAAAGCGGGCTATTCCAACTATCGT | | CCATTACCACCACCAACATCAACCA |
| *CbuCOMT*7 | | CATCTTATTCAGGAGTGGAGCACAC | | GGCAGCGATTCATAGCGATTCTTC |
| *CbuCOMT*8 | | TAGTGGATGTTGGTGGTGGAATTGG | | ATCGGCTTGAGGCACGCTAAC |
| *CbuCOMT*9 | | CCGAGCTATGCACGAACCATCC | | CCAATTCCACCACCAACATCCACTA |
| *CbuCOMT*10 | | GTTGGTGGTGGAATTGGAGCATCA | | ATCGGCTTTAGGCACGCTAACAAA |
| *CbuCOMT*11 | | GTGCCTCAAGCCGATGCCATT | | TTCCCTTTCTGACCTCTCCTTTCCA |
| *CbuCOMT*12 | | AAGGAATGGCTATCGGGCTGCT | | CAGGTTGTCGCTCCAGTCATGTAAT |
| *CbuCOMT*13 | | CACGAGTTCCTTGCTTGCTATGCTA | | TGACCACCAACATCCACCAAACAC |
| *CbuCOMT*14 | | AAGTCAGATGAAGAAGCCTGCGTAT | | GCAGCAAGTTCAGAAGCAGAAGC |
| *CbuCOMT*15 | | GCTCATACGCCGCTTTCTCTTCTT | | ATTGCTGTTGCCGTTGAGAGTCTT |
| *CbuCOMT*16 | | GCAGAGTAATGATAGTCGTCGTCCA | | AGCGGCGTGTGAGCGTAGTA |
| *CbuCOMT*17 | | GAAGGGATGGCTATCGGGATGCT | | CGTCGCTCCAGTCATGCAATACC |
| *CbuCOMT*18 | | GAAGGGATGGCTATCGGGATGCT | | CGTCGCTCCAGTCATGCAATACC |
| *CbuCOMT*19 | | GCAATTAGCAGCAGCAAGAACAAGG | | GCCATCATCGCCATGTCATAGAAGA |
| *CbuCOMT*20 | | TGTTGGATGTTGGTGGTGGTAATGG | | TCACACGGTGGAGCGATGGAA |
| *CbuCOMT*21 | | TGCTACACCCAAACACCACTTTCTC | | CCATTGGTTAGTGCTCGTGTCCTC |
| *CbuCOMT*22 | | GCTTGACAGAATCCTCCGCCTTC | | CGCCATAGAAACTCCATCGTCGTT |
| *CbuCOMT*23 | | CAGTTCGTGGGAGGGAGTATGTTTG | | CTCATCTTCTTCGCCGTCTTCGTT |
| *Actin* | | GATGATGCTCCAAGAGCTGT | | TCCATATCATCCCAGTTGCT |
