# Supplementary material for: Genome-wide analysis of the Catalpa bungei caffeic acid O-methyltransferase (COMT) gene family: identification and expression profiles in normal, tension, and opposite wood
Source: PeerJ. 2019 Mar 14;7:e6520. doi: 10.7717/peerj.6520 (PMC6421059; doi:10.7717/peerj.6520)
Supplement: Table S2 [file peerj-07-6520-s002.docx]

Table S2. The information of possible COMT genes in other Tubiflorae plants

| Species |  | Gene ID |
| --- | --- | --- |
| *Capsicum baccatum* |  | Capang00g002483 |
|  |  | Capang01g005362 |
|  |  | Capang01g005363 |
|  |  | Capang02g001654 |
|  |  | Capang03g001581 |
|  |  | Capang03g001584 |
|  |  | Capang03g001585 |
|  |  | Capang03g001587 |
|  |  | Capang03g001738 |
|  |  | Capang03g001741 |
|  |  | Capang06g000155 |
|  |  | Capang06g000156 |
|  |  | Capang06g000157 |
|  |  | Capang06g001637 |
|  |  | Capang06g002424 |
|  |  | Capang09g000149 |
|  |  | Capang10g000522 |
|  |  | Capang10g001299 |
|  |  | Capang10g001300 |
| *Salvia miltiorrhiza* |  | evm.model.C211107.2 |
|  |  | evm.model.scaffold10998.13 |
|  |  | evm.model.scaffold11905.1 |
|  |  | evm.model.scaffold1343.7 |
|  |  | evm.model.scaffold3607.2 |
|  |  | evm.model.scaffold6013.8 |
|  |  | evm.model.scaffold6013.8.1 |
|  |  | evm.model.scaffold6013.9 |
|  |  | evm.model.scaffold6088.4 |
|  |  | evm.model.scaffold6380.1 |
| *Mimulus guttatus* |  | Migut.E00608.1 |
|  |  | Migut.F00144.1 |
|  |  | Migut.G00684.1 |
|  |  | Migut.G00689.1 |
|  |  | Migut.G00731.1 |
|  |  | Migut.G00736.1 |
|  |  | Migut.H02110.1 |
|  |  | Migut.H02155.1 |
|  |  | Migut.N00314.1 |
|  |  | Migut.N00316.1 |
|  |  | Migut.N03027.1 |
| *Petunia axillaris* |  | Peaxi162Scf00092g00172.1 |
|  |  | Peaxi162Scf00313g00016.1 |
|  |  | Peaxi162Scf00401g00526.1 |
|  |  | Peaxi162Scf00401g00617.1 |
|  |  | Peaxi162Scf00517g00518.1 |
|  |  | Peaxi162Scf00517g00634.1 |
|  |  | Peaxi162Scf00912g00111.1 |
|  |  | Peaxi162Scf01698g00013.1 |
| *Solanum tuberosum* |  | PGSC0003DMT400000736 |
|  |  | PGSC0003DMT400002220 |
|  |  | PGSC0003DMT400003239 |
|  |  | PGSC0003DMT400020336 |
|  |  | PGSC0003DMT400020988 |
|  |  | PGSC0003DMT400022141 |
|  |  | PGSC0003DMT400022142 |
|  |  | PGSC0003DMT400022145 |
|  |  | PGSC0003DMT400027353 |
|  |  | PGSC0003DMT400029318 |
|  |  | PGSC0003DMT400031366 |
|  |  | PGSC0003DMT400031369 |
|  |  | PGSC0003DMT400031370 |
|  |  | PGSC0003DMT400034726 |
|  |  | PGSC0003DMT400038092 |
|  |  | PGSC0003DMT400045238 |
|  |  | PGSC0003DMT400045239 |
|  |  | PGSC0003DMT400047988 |
|  |  | PGSC0003DMT400047989 |
|  |  | PGSC0003DMT400051773 |
|  |  | PGSC0003DMT400051781 |
|  |  | PGSC0003DMT400051785 |
|  |  | PGSC0003DMT400056250 |
|  |  | PGSC0003DMT400065993 |
|  |  | PGSC0003DMT400065994 |
|  |  | PGSC0003DMT400065996 |
|  |  | PGSC0003DMT400067578 |
|  |  | PGSC0003DMT400070159 |
|  |  | PGSC0003DMT400070160 |
|  |  | PGSC0003DMT400075158 |
|  |  | PGSC0003DMT400081288 |
|  |  | PGSC0003DMT400083245 |
|  |  | PGSC0003DMT400083262 |
|  |  | PGSC0003DMT400095923 |
| *Sesamum indicum* |  | SIN 1001447 |
|  |  | SIN 1001450 |
|  |  | SIN 1002216 |
|  |  | SIN 1002291 |
|  |  | SIN 1002292 |
|  |  | SIN 1002293 |
|  |  | SIN 1005145 |
|  |  | SIN 1007064 |
|  |  | SIN 1009239 |
|  |  | SIN 1009240 |
|  |  | SIN 1009241 |
|  |  | SIN 1009243 |
|  |  | SIN 1013206 |
|  |  | SIN 1013207 |
|  |  | SIN 1013208 |
|  |  | SIN 1016796 |
|  |  | SIN 1016797 |
|  |  | SIN 1016804 |
|  |  | SIN 1017054 |
|  |  | SIN 1019144 |
|  |  | SIN 1019145 |
|  |  | SIN 1019146 |
|  |  | SIN 1019147 |
|  |  | SIN 1019148 |
|  |  | SIN 1019154 |
|  |  | SIN 1019158 |
|  |  | SIN 1019164 |
|  |  | SIN 1019727 |
|  |  | SIN 1019728 |
|  |  | SIN 1023742 |
|  |  | SIN 1025795 |
|  |  | SIN 1025797 |
| *Utricularia gibba* |  | Scf00334.g14220.t1 |
|  |  | Scf00334.g14222.t1 |
|  |  | Scf00334.g15683.t1 |
|  |  | Scf00334.g15684.t1 |
|  |  | Scf00334.g15685.t1 |
|  |  | Scf00700.g20944.t1 |
|  |  | Scf00700.g21947.t1 |
|  |  | unitig 748.g7272.t1 |
|  |  | unitig 748.g7274.t1 |
|  |  | unitig 749.g14189.t1 |
| *Solanum lycopersicum* |  | Solyc01g068550.2.1 |
|  |  | Solyc01g111900.2.1 |
|  |  | Solyc02g077510.2.1 |
|  |  | Solyc02g077520.2.1 |
|  |  | Solyc02g077530.1.1 |
|  |  | Solyc03g097700.2.1 |
|  |  | Solyc06g007960.2.1 |
|  |  | Solyc06g064500.2.1 |
|  |  | Solyc06g064510.2.1 |
|  |  | Solyc06g083450.2.1 |
|  |  | Solyc10g005060.2.1 |
|  |  | Solyc10g008120.2.1 |
|  |  | Solyc10g079540.1.1 |
|  |  | Solyc10g085830.1.1 |
|  |  | Solyc12g009110.1.1 |
|  |  | Solyc12g041960.1.1 |
|  |  | Solyc01g068550.2.1 |
