# Supplementary material for: Genome-wide analysis of the Catalpa bungei caffeic acid O-methyltransferase (COMT) gene family: identification and expression profiles in normal, tension, and opposite wood
Source: PeerJ. 2019 Mar 14;7:e6520. doi: 10.7717/peerj.6520 (PMC6421059; doi:10.7717/peerj.6520)
Supplement: File S3 [file peerj-07-6520-s005.docx]

>CbuCOMT16

MVLDKEAQARADVWKYAFGSINCRVMIVVVQLQIPDIMKKHGGAISLSDLSAAVGVPADNLYRIMRFSIHHGMFKKTEAPQRKVSDEVVYYAHTPLSLLLTIDNVGPFILLQGAGPHGNFGGLTVAALKSGNRPDFKNLNGNGNGNGNGNGNGNGNWDDPFYTTKEFTDAMACHARVATSAIIENCPEAFRGIRTLVDVGGRHGMALSMLIKGFPWIKGIAFDLPEVVAKARPVDGIQFVGGSMFETIPKADAIMLMWILHDWSDETCIDILKKCKEAVPADTGKVIIAEAVINEDGEEDEYTGAHLSLDMIMMDQLIEGKERTYKEWAHLIKAAGFSRHNVKNMKTLVSLIEAHP

>CbuCOMT17

MDEEARAKVEIWQYVLGFITTKVVKWTIDLKLPDAVESHGGPITLSQLSAAVGCPTAALHRIMRFLTHKGIFKKQINLSKSPDPESIYYCQTPLSRLLARDKLALYLLIQAGPSEELDMDFSAEYMKAGRGSGSDNLSLSEDTIWDAKVDATHDKLFREFLACHAKIVTTSLIDYCQEVFEGVGSLVDVGGHEGMAIGMLVKAFPWIRGTNFDLPDVIARASPIDGVEHVGGNMFESIPKADAVMLMSVLHDWSDDICLDILKKCKEAIPVDTGKVIIVEVVIDEEGRDEYTGTRLAMDLAIMTATIKGKERTNKEWAQLLNAAGFSRHIIKHMKAIESVIEAYP

>CbuCOMT18

MDEEARAKVEIWQYALGFITMKVVKWAIDLKLPDAVESHGGPITLSQLSAAVGCPTAALHRIMRFLTHKGIFKKQINLSKSPDPESIYYCQTPLSRLLARDKLAPYVLLQAGPPRELDMDFSAEDLKAGRGSGLDNLSLSEDKIWDAKADATHHKLFLEFLACHAKIVTTSLIDYCQEVFEGVGSLVDVGGHEGMAIGMLVKAFPWIRGTNFDLPDVIARASPIDGVEHVGGNMFESIPKADAVMLMSVLHDWSDDICIDILKKCKEAIPTDKGKVIIVEVVIDEEGGDEYTSARLAMDMTIMTVTINGKERTYKEWAQLLNAAGFNRHAIKHMKTIDSVIEAYPEEECHMPVTALAKNKHKVLIRANIPYILLIVKLEGKK

>CbuCOMT19

SQAKSQYVSRLMRLLVHSNFFIEVNISDNNPKEGYWLTPASRLLLKDEPLSIIPFLQVIADPIMIEPWHYLSKWLVNDHHQTPFEMAHGRTFWEQAERVPRLNHLFNEAMASDAGLVNLVVLRNSKQLFGGFESLVDVGGGTGATAGAISEAFPEMKCTVLDLPHVVAGLKGNKNLSFLGGDMFQAIPHADMVLLKWVLHDWNDEDSVRILKKCKDAISSSKNKGGKVMIIDMILNNHGGGIKAMEDQLFYDMAMMAYLNGKERTEKEWAKIFSDAGFSSYKIALGLGVRSLIELYP

>CbuCOMT23

MVLDEEAQARADVWKYAFGNVTSRVIIVVVQLEIPDIMKKHGGAISLSDLSAAVGVPDDKLYRIMRFLIHHGMFKKIEPPRSKVLDDVVYYAHTPLSLLLTMDNVGPFILLQGAGPHSNYEGLTVDALKIRNCPDFKTLNGESNWDNPLYATKVFTDGMACHARVATSAIIENCPEAFRGIRTLVDVGGRHGIALSMLIKGFPWIKGIAFDLPEVVAKAPSVDGIQFVGGSMFDTFPKADAVMLMWTLHNWGDEACIDILKKCKEAVPADTGKVIIAEVVINEDGEEDEYTGARLSLDMVMTGLLDRRQREDIQRMGTSSEGSGL

>CbuCOMT1

MALADGELSTEQLLEAQAHVWNHIFNFINSMSLKCAIELGIPNIIHKHGKPVTLSELVNALPICKSKSQYIYRLMRVLLNSNFFIKVNISNEDEDEERYWLTPSSHLLLKDASLTVAPFVLLVLDPVLTKPWHYLSEWLADDHHLSPFKMTHGMMFWEYAQHEPRLNNLFNEAMCSDTRLVTRVLKNYKTKQVFEGIKSLVDVGGGIGTMAKAIVDAFPGMKCIVLDLPHVVAGLQGTNNLTYVEGDMFQTIPPADAVFLKWILHDWDDEHCVKILKKCKEAIPAGKGGKVIIIDMVVGIYEGGAEAMEDQLFFDMLMMTLLNGKERSEKEWAELSLDAGFTGYKITPVFGVRSLIELYP

>*CbuCOMT*2

MALLNRVEYCTKDLFDAQGHVWNHIFNFINSMSLKCALQLCIPMKLSQLVNALPINKAKSNIVFCLMRVLIHSKFFTKIKISDDDNQNEGYWHTPASLFLLRDDPISIAPLALAMLDPAMIDPWHHVSEWFQNESSSSFVTKHGMSFREYGKIEEKMNRLFNEAMAGDERFFTSVAINECKQVFEVLKSMVDVGGGTGIVAKAIADALISWLEMYRSRSSTCC

>CbuCOMT4

MDEEARAQIDVWKYAYGFDAMRVVKCAIELGIPDVFGSRGSPMTLSELSSAVGCPENSLYRIMRFLTHNGIFKKKIISQDPPLFHYSQTPLSRLLTRDNMGLFVLVQAGPSGKQFGLTAEDLRAGKGSGLKPAADEMTMWSSGVVDEAYEKLFRDHMASHGKLGASKVINNCPEVFEGIESLVDVGGNDGTAIGMFVKAFPWIRGINFDLPQVVHEAPAIDGVLHVGGDMFESIPKADAIMLMSVLHDWSDEMCIEILKKCKEAIRTKTGKVIIVEVVIDEEGEEDEYMGARLLVDMMIMIATINGKERTTKEWIRLLNASGFSKYTIKHMRAIESIIEAYP

>CbuCOMT12

MDEEAQARVQIWNYALGFNSMRAVKCAIELGLPDVLENHGGPMTLSQLSATVGCPIPALRRLLRFLTHNGIFKKELNLSKSQDPESSYYSQTALSRLLTRDKMAPFVLLQADPPKVQYIGLTANDLKAGKGSGLDNLACSEDMMWNYELDPAYDKLFHDFLTYHAKIATTALIDYCQEVFEGIGCLVDVGGHEGMAIGLLVKAFPWIRGINFDLPDVIAGASAIDGVEHVGGNMFQSVPKADAVMLMWILHDWSDNLCIDILKKCKEAVPADTGKVIIVEAVIDEEGGDEYTSARLAMDITMMTVTTKGKERTYKEWAHLLNAAGFSKHTIKHMKAVESVIEAYP

>CbuCOMT13

MDEEAQARVEIWKYALGFNSMRAVKCAIELGLPDVLENHGGPMTLSQLSATVGCPIPALRRLLRFLTHNGIFKKELKLSKSQDPESSYYSQTALSRLLIRDKMATFVILQADPPAVQCIGLTAKDLKAGKGSGLDNRFPSEDIIWNVEVDAAFDKLLHEFLACYAKIATAALIDNCPAVFEGIGCLVDVGGHEGTAMGMLVKAFPWIRGINFDLPDVIAGASTIDGVEHVGGNMFECVPKADAVMLMRILHDWSDNLCIDILKKCKEAIPADTGKVIIVEAVIDEEGGDEYTSARLAMDITMMTVTINGKERTCKEWAQLLNAAGFSRHTIKHMKAVESVIEAYP

>CbuCOMT15

MVLDEEAQARADVWKYAFGSINTKVMIVVVQLQIPDIMKKHGGAISLSDLSAAVGVPADNLYRIMRFSIHHGMFKKTEPPQRKVSDDVVYYAHTPLSLLLTIDNVGPFILLQGAGPHGNFGGLTVAALKIGNRPDFKTLNGNSNGNGNGNGNWDDPFYATKVYTDAMACHARVATSAIIKNCPEAFRGIRTLVDVGGRHGMALSMLIKGFPWIKGIAFDLPEVVAKAPPVDGIQFVGGSMFEAIPKAEAIMLMWILHDWSDKACIDILKKCKEAIPADTGRVIIAEAVIKEDEEEDEYTGAQLSLDMIMMDLHIEGKERTYKEWAHLLKAAGFSRHNVKNMKTLVSVIEAYP

>CbuCOMT20

MDLTKSLKEVDEEAQAQVDIWQYIFGFAPMAVVKCAIELQIPDVLESHGGAMTLPELSAALGCSPSVLSRIMRYLIHRGIFKQKPTSQESQICYIQTPLSRLLLKNSMAAFILMESNPVMLAPWHNLKTRALTNGASAFKAANGADFWDYGSENPGYSKLFNDGMACHAKLAISNIVNHYPEAFKGIGSLLDVGGGNGTALRTLVKSCPWIRGINFDLPHVVSIAPPCDGIGHVGGDMFEMVPKADAAFLMLVLHDWSDDECIQILRNCREAIPTDTGKVIIAEVVVEEREEDKVTDAHLALDMAILVHTEKGKERTIKEWEYVVYAAGFTKYTIKHIEGEIISVIEAYS

>CbuCOMT21

MDIKTLKEVDEEVHAQVDIWQYIFGFVPMAVVKCAIELQIPDVLESHGGAMTLPELSTALGCSPSILSRIMRYLTHRGIFKHKLTSQGSQICYTQTPLSRLLMKNGANTMAALVLLESSPVMLAPWHNLRTRALTNGDSAFEAAHGGDVWDYATENPAHSKLINDAMACHAKLAIPTIVNRYPEVFKEISSLVDVGGGNGTALRTLVKYCPWIHGINFDLPHVVAVAPSCDGVEHVGGNMFEMVPKADAAFLMWVLHDWSDNECIQILTKCHEAIPKDTGKVIIAEAIIEEGEEDKFIDVRLALDMVMLAHTEKGKERTVKE

>CbuCOMT22

MSSSTKNLGAPTMASSDEESCLFALQLASASVLPMVLKSAIELDLLELIKKAGPGAFVSPAELAAQLPTTNQEARVMLDRILRLLASYDILNCSLKTLPDGSVERRYGLAPVCKFLTKNDDGVSMAPLLLMNHDKVLMESWYHLKDAVLDGGIPFNKAYGMSAFEYHGTDPRFNKVFNNGMSNHSTIIMKKILETYDGFEGLKTVVDVGGGTGAILSMIVSKYPSIKGINFDLPHVIEDAPSYPGVEHVGGDMFASVPKGDAIFMKWISHNWKDEHCLKFLKNCYEALPQNGKVILADCLLPEAPDSKLATKNAVHIDVIMLAHNPGGRERTENEFQALAKGAGFKLFKKVCCAYNTWIMELCK

>CbuCOMT14

MDNKSDEEACVFAFQLAAGSVLPMALYTAIELDLLELIKKAGPEASASASELAAQHPTTNPDAADMIDSILRLLASHSVLICSLKQLADGGVERRYSLAPVCKFLTRNEDGVSVGPLCLLLQDNITWVRVEHVSGDMFVSMTKADAIFMKWVCHGWRDSHCEKLLKNCYEALPENGKVIIAEVIVPDNPNGGQSSSWAAQGDMTMLAYTSGGGKERSEREFEALAKIVGFKQLIKVCSAYSNWIMEFHK

>CbuCOMT6

MEVIKPIKEVEEEGQAQVDIWEYIFAFIPMAVVKCAIELQIPDVLESHGGAMTLPELSAALGCSPSILSRIMRYLTHRGIFKQKLTSQICYTQTPLSRLLMKNGANTMAALVLLESSPVMLAPWHNLRTRALTNGDSAFEAAHGGDVWDFATENPAHSKLINDAMACHAKRAIPTIVNRYPEVFKEISSLVDVGGGNGTALRTLVKYCPWIHGINFDLPHVVAVAPSCDGVEHVGGNMFEMVPKADAAFLMWVLHDWSDNECIQILTKCREAIPKDTGKVIIAEAIIEEGEEDKFIDVRLALDMVMLAHTEKGKERTIKE

>CbuCOMT7

MIFFFGRYHLKDAILEGGTPFNRAHEYKGFEGLKSLVDVGGGIGTSLNMIISKYPSIKGINFDLPHVIQDAPSYSGVEHTSGDMFVSVPKADAIFMKWICHDWGDSHCEKLLKNRYESLPENGKVIIGEAIRSEDPNSSLQSALSDVIMLAFNPYGKQRSEREFEALAKKAGFKHLIKVCRASHIWIIEFHK

>CbuCOMT8

MDNQSDEEACLFALQLATGSVLPMVLKTAIELDLLELIKKAGPEASASASELVAQLPTNNPDAANMIDRILRLLAAHSVLVCSLKPLPDGGVERRYSLAPVCKFLTRNEDGVSVGPTCLMIQDKVLMEPWYHLKDAIIEGGIPFNRAYGMNAFEYPAKDPRFNRVFNQAMYEQSTIFMKKILEEYKGFEGLKSLVDVGGGIGASLKMIISKYPSIKGINFDLPHVIQNAPSYPGVEHISGNMFVSVPQADAIFMKN

>CbuCOMT9

MVGSAVESWLQVDVVPFLGIHPPTTVLPTTNPDAADMIDRILRLLASHSVLICSLKQLPDGGVERRYSLAPACVNRVLMEPGYHLNDAILEGGISFDRVYGMNAFEYLAKDPRFNRVFNRAMHEPSTIIMTKILEKYKGFEGLKSLVDVGGGIGSSLNMIISEYPSIKGINFDLPHVIQVAPPHPGVEHNSGDMFISVPKADAIFMKWVCHGWRDSHCEKLLKNCYEALPENGKVIIAEVIVPDNPNTGQSSSWAAQGDMIMLAYTSGGKERSEREFEALAEKAGFKHLIKVCSAYSNWLMEFHK

>CbuCOMT10

MDNYKPDEEACLFAFQLVSGSALPMVLQTAIELDLLELIKKSGPEASASASELAAQLPTSNPDAAHMIDRILRLLAVHSVLICSLKKLPDGGVERRYSLAPVCKFLTRNEDGVSVSPLCLLIQDRVLVEPRYLLKDAILEGGISFDRAYGMNAFEYLAKDPRFNRVFNRAMHEPSTIIMAKILEKYKGFEGLKSLVDVGGGIGASLNMIISKYPSIKGINFDLPHVIQDAPPFPGVEHIRGDMFVSVPKADAILLKWVCHDWSDSSCEKLLKNCYEALPENGKVIVADAILPEDPNSGQSFFWATQIDVIMLAYNPGGKERSEREFEALAKKAGFKHLIKVCSAYADWVMEFHK

>CbuCOMT11

MDNQSDEEACLFALQLATGSVLPMVLKTAIELDLLELIKKAGPEASASASELVAQLPTNNPDAANMIDRILRLLAAHSVLVCSLKPLPDGGVERRYSLAPVCKFLTRNEDGVSVGPTCLMIQDKVLMEPWYHLKDAILEGGIPFNRAYGMNAFEYPAKDPRFNRVFNQAMYEQSTIFMKKILEEYKGFEGLKSLVDVGGGIGASLKMIISKYPSIKGINFDLPHVIQNAPSYPGVEHISGNMFVSVPQADAIFMKWICHDWSDSHCEKLLKNCFEALPENGKVIIAETILPDDPNSGPSSLRAAQADVIMLAYNPGGKERSEREFEALAEKAGFKHLIKVCSAFNIWIMEFHK

>CbuCOMT3

MENAINSNTNSANLEEDEAFVQAVAAIVSFALPVALNTAMELDLFNIIRKAGEGAAVLPSDIAARLIPISSLPEAAAGGIDCLLRLLASHSLLTCCTSELANGATETRYGLAPAGKFFVRDGNGASFAAHHEFLRCQAGLVEGCNKLKDAVLGGGNPFERAYGTSIYEYMKSKPDYSRTFHDFMTSFSVMIMKRVCEKYNGFEGLSSIVNVGGGSGATLDVIISRYPSIHGINFDLPEVIQSAPSYKGVHHISGDMFVQVPQGDAILMKFILHNWNDDRCVQVLKNCYEALPNMGKVIIVDYILPDIPQDDIHSKMVSHVDYTMLMLCGSRERTKDEFEVLARKSGFSEFKVVCNAHCVWVMEFIKYG

>CbuCOMT5

MDVTKSLKEVDEEVQAQVDIWQYIFGLAPMAVVKCAVELQIPDVLESHGGAMTLPELSAALGCTPSVLSRIMRYLIHRGIFKQKTTSQESQICYIQTSLSRLLMKNSMGAFLLMESNPVMLAPWHNLRACALAKGASAFKAANGADLWDYGSENPGHSKLFNDAMACHAKLAISNIVNRYPEAFKGIRSLVDVGGGNGTALRTLVKSCPWIRGINFDLPHVVSIAPPCDGIEHVGGDMFEMVPKADAAFLMWVLHDWSDDECIQILRNCREAIPKDTGKVIIAEAMIEEREEDKVTDARLALDMVILVHTEKGKERTIKEWEYVVYAAGFTKCTIKHIEVVKCAIKLQIPDVLESHGGAMTLPKLSAATLGRSSSVLSRIMRLKMGRIYGIMDPKMLAIASHSMMQWLAMLNWLFQGSLIVILRCMFKGIGSLVDVGGGNGTVLHTLVKSCPWIRGINFDLQHVVSAAPSCDGIEDMFEMVPTADAVFLMGIG
