# Supplementary material for: Genome-wide analysis of the Catalpa bungei caffeic acid O-methyltransferase (COMT) gene family: identification and expression profiles in normal, tension, and opposite wood
Source: PeerJ. 2019 Mar 14;7:e6520. doi: 10.7717/peerj.6520 (PMC6421059; doi:10.7717/peerj.6520)
Supplement: File S4 [file peerj-07-6520-s006.docx]

>CbuCOMT23

ATGGTGCTAGATGAAGAAGCACAAGCTCGTGCAGATGTCTGGAAATATGCTTTCGGAAATGTCACCTCCAGAGTAATAATAGTCGTCGTCCAGCTTGAAATACCCGATATAATGAAAAAACACGGCGGTGCTATCTCACTCTCCGACCTCTCCGCCGCCGTGGGAGTCCCCGACGATAAACTCTACCGCATAATGAGATTCTTAATCCATCATGGCATGTTTAAGAAAATTGAGCCCCCACGGAGCAAAGTCTTAGACGACGTCGTATACTATGCTCACACGCCACTTTCTCTTCTTTTAACAATGGACAACGTCGGACCCTTCATACTGCTGCAGGGGGCCGGTCCCCATTCAAACTATGAGGGCTTGACTGTGGATGCTTTGAAAATCAGAAATTGCCCCGATTTTAAGACTCTCAACGGCGAGAGCAATTGGGACAATCCACTCTATGCGACAAAGGTGTTTACGGACGGTATGGCGTGCCATGCTAGGGTGGCAACATCGGCGATTATTGAAAACTGTCCGGAAGCTTTCCGAGGAATTAGGACGTTGGTGGATGTTGGGGGCCGCCATGGGATAGCCCTTAGTATGTTGATAAAGGGATTTCCATGGATTAAGGGGATTGCTTTTGATCTTCCTGAGGTTGTGGCTAAGGCTCCTTCTGTTGATGGGATTCAGTTCGTGGGAGGGAGTATGTTTGATACTTTCCCAAAAGCAGATGCAGTTATGCTCATGTGGACATTGCATAATTGGGGCGACGAAGCGTGCATAGACATTCTCAAGAAATGCAAAGAAGCTGTTCCGGCAGACACCGGAAAAGTGATCATCGCAGAAGTTGTCATAAACGAAGACGGCGAAGAAGATGAGTACACAGGTGCCCGTTTGTCGCTGGATATGGTAATGACGGGCCTACTTGATCGAAGGCAAAGAGAGGACATACAAAGAATGGGCACATCTTCTGAAGGCAGCGGGCTTTAG

>CbuCOMT18

ATGGACGAAGAAGCTCGAGCTAAAGTAGAAATTTGGCAATACGCTTTAGGTTTCATTACAATGAAAGTGGTGAAATGGGCCATAGATCTTAAATTGCCCGACGCCGTCGAAAGTCACGGCGGCCCCATCACTCTCTCTCAACTATCCGCCGCCGTGGGCTGCCCCACCGCCGCTCTCCACCGCATCATGCGCTTCTTGACTCACAAAGGAATCTTCAAAAAGCAGATTAACTTAAGCAAAAGCCCCGATCCTGAATCAATTTACTATTGCCAAACGCCCCTTTCCCGCCTCCTCGCAAGAGATAAATTGGCCCCTTATGTACTTCTCCAGGCTGGTCCTCCCAGGGAGCTGGACATGGATTTCAGTGCAGAAGATTTGAAAGCTGGAAGAGGGTCTGGTTTGGATAATCTTTCTCTTTCTGAGGATAAAATCTGGGATGCAAAAGCTGATGCAACTCATCATAAGCTGTTTCTTGAGTTTTTAGCTTGTCATGCTAAAATTGTGACCACGTCGCTTATCGATTATTGTCAGGAGGTTTTTGAAGGAGTTGGGTCTTTGGTGGATGTTGGTGGTCATGAAGGGATGGCTATCGGGATGCTGGTTAAGGCTTTTCCTTGGATTCGGGGGACTAATTTTGATCTCCCAGATGTGATCGCTAGGGCTTCCCCCATTGATGGGGTGGAGCATGTTGGAGGAAACATGTTTGAAAGTATTCCAAAAGCTGATGCAGTTATGCTCATGTCGGTATTGCATGACTGGAGCGACGACATATGCATAGACATCCTCAAGAAATGCAAAGAAGCTATTCCAACAGACAAAGGGAAAGTGATAATTGTTGAAGTTGTAATAGACGAAGAAGGAGGGGATGAGTATACGAGTGCTCGTTTGGCAATGGATATGACAATCATGACCGTAACAATAAATGGGAAGGAGAGAACTTATAAGGAATGGGCACAGCTCCTAAATGCCGCAGGATTCAACAGACACGCTATCAAACACATGAAAACTATTGACTCTGTCATCGAAGCTTATCCGGAGGAAGAATGCCACATGCCGGTGACGGCTCTGGCGAAAAACAAACATAAAGTGTTGATCAGAGCAAATATACCATACATTTTATTAATAGTTAAATTAGAGGGCAAAAAGTGA

>CbuCOMT17

ATGGACGAAGAAGCTCGAGCTAAAGTAGAAATTTGGCAATACGTTTTAGGTTTCATTACAACGAAAGTGGTGAAATGGACCATAGATCTTAAATTGCCCGACGCCGTCGAAAGTCACGGCGGCCCCATCACTCTCTCTCAACTATCCGCCGCCGTGGGCTGCCCCACCGCCGCTCTCCACCGCATCATGCGCTTCTTGACTCACAAAGGAATCTTCAAAAAGCAGATTAACTTAAGCAAAAGCCCCGATCCTGAATCAATTTACTATTGCCAAACGCCCCTTTCCCGCCTCCTCGCAAGAGATAAATTGGCCCTTTATTTACTTATCCAGGCGGGTCCTTCAGAGGAGCTGGACATGGATTTCAGTGCAGAATATATGAAAGCTGGAAGAGGGTCTGGTTCGGATAATCTTTCTCTTTCTGAGGATACAATCTGGGATGCAAAAGTTGATGCAACTCATGATAAGCTGTTTCGTGAGTTTTTAGCTTGTCATGCTAAAATTGTGACCACGTCGCTTATCGATTATTGTCAGGAGGTTTTTGAAGGAGTTGGGTCTTTGGTGGATGTTGGTGGTCATGAAGGGATGGCTATCGGGATGCTGGTTAAGGCTTTTCCTTGGATTCGGGGGACTAATTTTGATCTCCCAGATGTGATCGCTAGGGCTTCCCCCATTGATGGGGTGGAGCATGTTGGAGGAAACATGTTTGAAAGTATTCCAAAAGCTGATGCAGTTATGCTCATGTCGGTATTGCATGACTGGAGCGACGACATATGCTTAGACATCCTCAAGAAATGCAAAGAAGCTATTCCAGTAGACACAGGGAAAGTGATAATTGTTGAAGTTGTAATAGACGAAGAAGGACGCGATGAGTATACGGGTACTCGTTTGGCAATGGATCTGGCAATCATGACCGCAACAATAAAAGGGAAGGAGAGAACTAATAAGGAATGGGCACAACTTCTAAATGCCGCAGGCTTTAGCAGACACATTATCAAACACATGAAAGCTATTGAATCTGTCATTGAAGCCTATCCATAG

>CbuCOMT12

ATGGACGAAGAAGCTCAAGCTCGAGTGCAGATATGGAACTACGCTTTGGGTTTTAATTCAATGAGAGCGGTGAAGTGCGCCATCGAACTAGGATTACCCGACGTCCTCGAAAATCACGGCGGCCCCATGACACTCTCTCAACTCTCCGCCACCGTCGGCTGCCCCATTCCCGCTCTCCGCCGCCTCCTCCGTTTCTTAACTCACAACGGGATCTTCAAAAAGGAGCTTAACTTAAGCAAAAGCCAAGATCCAGAATCGTCTTACTATTCCCAAACTGCCCTTTCCCGCCTCCTCACAAGAGATAAAATGGCCCCTTTTGTACTTCTCCAAGCGGATCCTCCAAAGGTGCAATATATTGGCTTAACTGCAAATGATCTGAAAGCTGGAAAGGGATCTGGTTTGGATAATCTTGCCTGTTCTGAGGATATGATGTGGAATTATGAACTTGATCCAGCTTATGATAAGCTGTTTCATGACTTTTTAACTTACCATGCTAAGATTGCGACGACGGCGCTTATCGATTATTGTCAGGAGGTGTTTGAAGGAATTGGGTGTTTGGTGGATGTTGGTGGTCATGAAGGAATGGCTATCGGGCTGCTGGTTAAGGCTTTTCCTTGGATTCGAGGGATTAATTTTGATCTCCCGGATGTGATCGCCGGGGCTTCCGCCATTGATGGGGTGGAGCATGTTGGAGGAAACATGTTTCAAAGTGTTCCAAAAGCTGATGCAGTTATGCTCATGTGGATATTACATGACTGGAGCGACAACCTGTGCATAGACATCCTCAAGAAATGCAAAGAAGCTGTTCCAGCAGACACAGGGAAAGTGATCATTGTTGAAGCTGTAATAGATGAAGAAGGAGGCGATGAGTATACGAGTGCTCGTTTGGCAATGGATATAACAATGATGACAGTAACAACAAAAGGGAAGGAGAGAACTTATAAGGAATGGGCACATCTCCTAAATGCCGCAGGCTTCAGCAAACACACTATCAAACACATGAAAGCTGTTGAATCTGTCATTGAAGCCTATCCATAG

>CbuCOMT13

ATGGACGAAGAAGCTCAAGCTCGAGTGGAGATATGGAAATACGCATTGGGCTTCAATTCAATGAGAGCGGTGAAATGCGCCATTGAACTAGGACTACCTGACGTCCTCGAAAATCACGGCGGCCCCATGACACTCTCTCAACTCTCCGCCACCGTCGGCTGCCCCATTCCCGCTCTCCGCCGCCTCCTCCGTTTCTTAACTCACAACGGGATCTTCAAAAAGGAGCTTAAATTAAGCAAAAGCCAAGATCCAGAATCATCTTACTATTCCCAAACTGCCCTTTCCCGCCTCCTCATCAGAGATAAAATGGCCACTTTTGTAATTCTGCAAGCGGATCCTCCAGCAGTGCAGTGTATTGGTTTAACTGCAAAAGATCTGAAAGCTGGAAAGGGATCTGGTTTGGATAATAGATTTCCTTCTGAGGATATAATCTGGAATGTGGAAGTTGATGCAGCTTTTGATAAGCTGCTTCACGAGTTCCTTGCTTGCTATGCTAAGATTGCAACGGCGGCGCTTATCGATAACTGTCCGGCGGTCTTCGAAGGAATTGGGTGTTTGGTGGATGTTGGTGGTCATGAGGGGACGGCCATGGGGATGTTGGTGAAGGCTTTTCCTTGGATTCGAGGGATTAATTTTGATCTCCCAGATGTGATCGCCGGGGCTTCCACCATTGATGGAGTAGAGCATGTTGGAGGAAACATGTTTGAATGTGTCCCAAAAGCTGATGCAGTTATGCTCATGCGTATATTACATGACTGGAGCGACAACCTATGCATAGACATCCTCAAGAAATGCAAAGAAGCTATTCCAGCAGACACAGGGAAAGTGATCATTGTTGAAGCTGTAATAGATGAAGAAGGAGGCGATGAGTATACGAGTGCTCGTTTGGCAATGGATATAACAATGATGACCGTAACAATAAATGGGAAGGAAAGAACTTGTAAGGAATGGGCACAGCTCCTAAATGCCGCAGGCTTCAGCAGACACACTATCAAACACATGAAAGCTGTTGAATCTGTCATTGAAGCCTATCCATAG

>CbuCOMT15

ATGGTGCTAGACGAAGAAGCCCAAGCTCGTGCAGATGTCTGGAAATACGCTTTCGGATCTATCAACACCAAAGTAATGATAGTCGTCGTCCAGCTCCAAATACCCGATATAATGAAAAAACACGGTGGCGCTATCTCACTCTCCGACCTCTCCGCCGCCGTGGGTGTCCCTGCCGATAACCTCTACCGCATAATGAGATTCTCAATCCATCACGGCATGTTCAAGAAAACAGAGCCCCCACAGAGAAAAGTCTCAGACGACGTCGTATACTACGCTCATACGCCGCTTTCTCTTCTTTTAACAATTGACAACGTCGGACCCTTCATTCTGCTGCAGGGGGCCGGTCCCCATGGAAACTTTGGGGGCTTAACTGTGGCTGCTTTGAAAATCGGAAATCGCCCCGATTTTAAGACTCTCAACGGCAACAGCAATGGAAACGGCAACGGAAACGGCAATTGGGACGATCCATTCTATGCGACAAAGGTATATACGGACGCTATGGCGTGCCACGCTAGGGTGGCAACATCGGCGATTATTAAGAACTGTCCAGAAGCTTTCCGAGGAATTAGGACGTTGGTGGATGTCGGCGGCCGCCATGGGATGGCCCTTAGTATGTTGATAAAGGGATTTCCATGGATTAAGGGGATTGCTTTTGATCTTCCTGAGGTTGTGGCTAAGGCTCCTCCTGTTGATGGGATTCAGTTCGTTGGAGGGAGTATGTTTGAAGCTATCCCAAAAGCTGAAGCAATTATGCTCATGTGGATATTGCACGATTGGAGCGATAAAGCTTGCATAGACATCCTCAAGAAATGCAAAGAAGCCATTCCGGCAGACACCGGAAGAGTGATCATCGCAGAAGCAGTCATAAAAGAAGACGAAGAAGAAGATGAGTACACAGGTGCCCAATTGTCGCTGGATATGATAATGATGGACCTACATATCGAAGGCAAAGAGAGGACATACAAAGAATGGGCGCATCTTCTCAAGGCAGCTGGCTTTAGCAGACACAACGTTAAAAATATGAAAACTCTTGTCTCTGTAATTGAGGCCTATCCCTAA

>CbuCOMT20

ATGGACTTAACTAAATCACTCAAAGAAGTGGATGAAGAAGCGCAGGCACAAGTAGATATATGGCAGTATATATTTGGCTTCGCTCCAATGGCAGTAGTAAAATGTGCCATTGAACTCCAAATCCCCGATGTCTTAGAAAGCCACGGCGGAGCCATGACATTACCGGAGCTATCTGCCGCTCTTGGCTGCTCCCCTTCTGTGCTCAGCCGCATAATGAGGTACTTAATCCACCGCGGCATCTTTAAGCAGAAGCCCACAAGCCAAGAATCACAAATTTGCTACATCCAAACACCCCTTTCTCGTCTCCTTTTGAAAAATAGCATGGCTGCTTTTATTCTAATGGAAAGCAACCCTGTGATGCTTGCTCCATGGCACAATCTGAAAACACGCGCATTAACCAATGGGGCTTCAGCGTTTAAGGCTGCAAATGGGGCGGATTTCTGGGATTATGGATCCGAAAATCCTGGCTATAGCAAACTATTCAATGATGGAATGGCTTGCCATGCGAAGTTGGCTATTTCAAATATTGTTAATCATTATCCTGAGGCGTTTAAGGGCATAGGATCTTTGTTGGATGTTGGTGGTGGTAATGGGACGGCTCTGCGTACATTGGTGAAGTCTTGTCCATGGATTCGTGGGATTAACTTCGACCTCCCACATGTTGTTTCCATCGCTCCACCGTGTGATGGTATTGGGCATGTTGGTGGGGACATGTTTGAGATGGTGCCCAAGGCTGATGCTGCTTTTCTCATGTTGGTACTACATGATTGGAGTGATGATGAATGTATCCAGATATTGAGAAATTGCCGAGAAGCCATTCCCACGGATACAGGAAAAGTGATCATTGCAGAGGTAGTGGTCGAAGAAAGAGAAGAGGATAAGGTCACTGATGCTCATTTGGCATTGGATATGGCAATTCTGGTTCACACAGAGAAAGGAAAAGAGAGGACTATTAAAGAATGGGAATATGTGGTTTATGCAGCTGGCTTCACTAAGTATACTATAAAACATATTGAAGGTGAAATAATATCTGTTATTGAGGCATATTCATAA

>CbuCOMT21

ATGGACATAAAAACACTCAAAGAAGTAGATGAAGAAGTGCACGCCCAAGTAGATATATGGCAATATATATTTGGCTTCGTTCCAATGGCAGTAGTAAAATGCGCCATCGAACTCCAAATCCCCGACGTCTTAGAAAGCCACGGCGGAGCCATGACGCTACCGGAGCTATCCACCGCTCTTGGCTGCTCCCCTTCCATCCTCAGTCGCATAATGAGGTACTTAACCCACCGCGGTATCTTCAAGCACAAGCTTACAAGCCAAGGCTCCCAAATTTGCTACACCCAAACACCACTTTCTCGTCTCCTCATGAAAAATGGAGCCAATACCATGGCTGCTCTTGTTTTGCTCGAAAGTAGCCCTGTGATGCTTGCTCCATGGCACAATCTGAGGACACGAGCACTAACCAATGGGGATTCAGCATTTGAGGCTGCACATGGGGGAGATGTATGGGATTATGCTACTGAAAATCCTGCCCATAGTAAGCTAATTAACGATGCAATGGCTTGCCATGCTAAGCTGGCTATTCCAACTATCGTTAATCGTTATCCTGAGGTGTTTAAGGAGATTAGCTCATTGGTTGATGTTGGTGGTGGTAATGGGACAGCTCTTCGTACGTTGGTGAAGTATTGTCCATGGATTCATGGGATTAACTTCGATCTCCCACATGTGGTTGCCGTCGCCCCGTCGTGTGATGGTGTTGAGCATGTTGGTGGAAATATGTTTGAAATGGTTCCTAAGGCTGATGCTGCTTTTCTTATGTGGGTGTTGCATGATTGGAGTGACAATGAATGTATTCAGATATTGACAAAATGTCATGAAGCTATTCCCAAGGACACTGGAAAAGTGATCATCGCAGAGGCAATTATTGAAGAAGGAGAAGAGGACAAGTTTATTGATGTTCGTCTGGCTTTGGACATGGTTATGCTGGCTCACACAGAGAAAGGAAAAGAAAGAACTGTTAAAGAATGA

>CbuCOMT2

ATGGCCTTGCTGAATAGAGTAGAGTACTGCACGAAAGATCTTTTCGATGCTCAGGGTCACGTTTGGAACCACATTTTTAACTTCATAAATTCCATGTCTCTAAAATGTGCACTTCAATTATGCATACCCATGAAACTTTCTCAATTAGTCAATGCCCTCCCAATCAACAAAGCAAAATCCAACATTGTCTTTTGTCTAATGCGCGTGTTAATTCATTCCAAGTTCTTCACCAAGATCAAGATCTCTGATGATGATAACCAGAACGAGGGCTATTGGCACACACCGGCTTCACTTTTCCTGTTGAGAGACGACCCCATAAGCATCGCGCCTCTTGCCCTTGCCATGCTCGACCCGGCAATGATAGATCCATGGCATCATGTGAGTGAATGGTTTCAGAATGAGTCTTCCTCATCATTCGTCACCAAACATGGGATGAGTTTTCGGGAATACGGTAAGATTGAAGAAAAGATGAATCGATTATTTAATGAGGCGATGGCTGGGGATGAACGGTTTTTCACTAGTGTAGCCATTAATGAATGTAAACAAGTGTTTGAGGTGTTGAAATCAATGGTGGATGTTGGAGGTGGCACTGGAATAGTGGCCAAGGCTATTGCTGATGCCTTAATTTCCTGGCTTGAAATGTACCGTTCTCGATCTTCCACATGTTGTTGA

>CbuCOMT1

ATGGCATTGGCCGATGGAGAGCTATCCACAGAGCAACTTCTTGAAGCTCAAGCTCATGTATGGAACCACATATTCAACTTCATAAACTCCATGTCTTTAAAATGTGCAATTGAACTAGGCATACCAAACATCATCCACAAACACGGAAAACCAGTCACACTTTCTGAATTAGTCAATGCCCTCCCCATTTGCAAATCAAAATCTCAATATATCTATCGTTTAATGCGGGTCTTACTCAACTCCAACTTCTTCATCAAAGTCAACATATCTAACGAAGATGAAGATGAAGAGCGTTATTGGCTGACGCCATCCTCTCATCTCCTCTTGAAGGACGCATCCTTGACTGTGGCACCCTTCGTGCTACTCGTACTGGATCCAGTTTTGACAAAGCCATGGCACTATCTGAGCGAATGGCTTGCAGACGATCACCACCTCTCACCCTTCAAGATGACACACGGAATGATGTTTTGGGAGTACGCACAGCACGAGCCACGGCTAAATAACTTGTTCAATGAAGCTATGTGTAGCGACACGAGGCTAGTGACTCGTGTACTCAAAAACTACAAAACTAAACAAGTGTTTGAAGGGATCAAGTCATTAGTGGATGTAGGTGGTGGCATTGGGACAATGGCTAAGGCTATTGTGGATGCATTCCCGGGCATGAAATGTATTGTCCTCGATCTCCCACATGTTGTCGCTGGCTTGCAAGGGACTAATAACTTGACCTATGTTGAGGGAGACATGTTTCAAACTATTCCTCCTGCTGATGCTGTTTTCCTCAAGTGGATATTGCATGATTGGGACGACGAACACTGTGTCAAAATATTGAAGAAATGCAAAGAAGCTATACCTGCAGGAAAGGGAGGAAAGGTGATAATAATCGATATGGTTGTGGGCATTTATGAAGGAGGGGCTGAGGCAATGGAAGATCAACTATTCTTTGATATGTTGATGATGACTCTTCTAAATGGAAAAGAAAGAAGTGAGAAAGAATGGGCTGAGCTATCCTTAGACGCTGGCTTCACTGGCTACAAGATTACTCCTGTATTTGGTGTGAGGTCTCTCATTGAGCTTTATCCATGA

>CbuCOMT22

ATGAGCTCATCAACCAAGAATCTTGGAGCTCCAACAATGGCTTCTTCAGATGAAGAATCTTGCCTATTCGCTTTGCAATTAGCCAGTGCTTCCGTACTTCCAATGGTTCTCAAATCCGCCATTGAGCTCGATTTGCTTGAGCTCATCAAGAAAGCCGGCCCCGGTGCCTTTGTTTCGCCGGCGGAACTCGCCGCCCAGCTTCCCACCACCAATCAGGAGGCGCGTGTAATGCTTGACAGAATCCTCCGCCTTCTCGCGAGCTATGATATTCTGAACTGCAGCCTGAAAACGCTGCCGGATGGCAGCGTTGAGCGGCGGTATGGGTTGGCGCCGGTTTGTAAGTTCTTGACGAAGAACGACGATGGAGTTTCTATGGCGCCTTTGTTGCTCATGAACCATGATAAAGTCCTTATGGAGAGCTGGTACCATCTAAAAGATGCAGTTCTTGATGGTGGAATTCCCTTCAACAAAGCCTATGGAATGAGTGCCTTTGAGTACCATGGGACAGACCCAAGATTTAACAAGGTGTTTAACAATGGAATGTCTAATCATTCAACCATAATTATGAAGAAAATTCTTGAAACATATGATGGTTTTGAGGGCCTGAAAACTGTGGTGGATGTTGGTGGAGGAACAGGAGCCATACTCAGTATGATTGTCTCCAAGTATCCTTCCATTAAGGGCATTAACTTTGACTTGCCTCATGTTATTGAAGATGCTCCATCTTATCCAGGTGTGGAACATGTCGGTGGAGACATGTTTGCGAGTGTGCCCAAAGGGGATGCCATTTTCATGAAGTGGATTTCCCATAATTGGAAAGATGAACATTGCCTCAAATTCTTGAAAAATTGCTATGAAGCCCTTCCACAAAATGGGAAAGTGATTCTGGCTGACTGTCTTCTCCCAGAGGCCCCAGACAGCAAACTTGCCACTAAGAATGCTGTCCATATTGATGTGATCATGTTGGCTCATAATCCAGGTGGTAGGGAAAGGACAGAAAATGAATTTCAGGCACTGGCTAAGGGGGCTGGCTTTAAGCTGTTCAAGAAGGTTTGTTGTGCTTACAACACTTGGATTATGGAACTGTGTAAGTGA

>CbuCOMT3

ATGGAAAACGCCATTAACAGTAACACCAACTCTGCAAATCTTGAAGAAGACGAAGCATTTGTCCAGGCTGTAGCTGCTATTGTATCTTTTGCTCTCCCTGTGGCTTTGAACACTGCCATGGAACTCGATCTCTTTAATATCATCAGAAAAGCCGGCGAGGGGGCTGCCGTTTTGCCTTCTGATATCGCCGCCCGCCTAATTCCAATTTCCAGCCTGCCGGAAGCAGCTGCCGGCGGGATCGATTGCTTGCTTCGACTGCTTGCGAGTCACTCTCTACTCACTTGCTGCACAAGTGAACTTGCTAATGGCGCCACTGAAACCCGATACGGCCTTGCGCCGGCGGGGAAGTTCTTTGTTCGAGACGGGAATGGAGCTTCGTTTGCTGCTCACCATGAATTCTTGCGTTGTCAAGCTGGATTAGTGGAGGGCTGTAACAAGTTGAAAGATGCAGTTCTTGGAGGTGGGAATCCATTTGAAAGGGCTTACGGCACGTCTATATATGAGTACATGAAATCAAAACCCGATTATAGTAGAACATTTCACGATTTCATGACAAGTTTTAGTGTTATGATCATGAAACGTGTTTGTGAAAAATACAATGGTTTTGAGGGGTTAAGTTCAATCGTAAACGTTGGGGGTGGTAGTGGTGCCACCCTTGATGTCATTATTTCAAGGTATCCTTCCATTCATGGGATCAACTTTGATTTGCCAGAGGTAATACAATCTGCCCCATCTTATAAGGGAGTACATCATATTAGTGGAGATATGTTTGTCCAAGTGCCACAAGGAGATGCCATCTTGATGAAGTTTATATTGCACAACTGGAATGATGATCGATGTGTACAAGTCCTGAAAAACTGCTACGAGGCATTGCCAAACATGGGGAAAGTGATTATAGTGGACTATATTCTTCCAGACATTCCTCAAGATGATATTCATTCAAAAATGGTTTCACATGTCGATTATACGATGTTAATGTTGTGTGGATCAAGGGAAAGGACAAAGGATGAATTTGAGGTATTGGCTAGGAAGTCAGGGTTTTCTGAATTTAAGGTTGTTTGTAATGCCCATTGTGTTTGGGTGATGGAATTTATTAAATATGGATAA

>CbuCOMT19

AGCCAAGCCAAATCCCAATATGTCTCTCGCTTAATGCGCCTATTAGTTCATTCAAACTTCTTCATCGAAGTCAACATCTCTGATAACAATCCAAAAGAGGGCTACTGGCTCACACCAGCCTCTCGTCTCCTCCTTAAAGATGAGCCCTTGAGCATCATACCTTTCTTACAAGTCATAGCGGACCCAATTATGATCGAGCCATGGCATTATCTGAGCAAATGGCTTGTCAACGACCACCACCAGACGCCGTTTGAAATGGCTCATGGCCGGACATTTTGGGAGCAGGCGGAACGGGTCCCAAGGCTTAACCACTTGTTCAATGAAGCCATGGCTAGTGATGCAGGGCTCGTGAATCTTGTAGTACTTAGAAATTCCAAACAGTTGTTTGGAGGGTTTGAGTCTTTGGTGGATGTCGGTGGTGGCACCGGAGCAACGGCTGGGGCAATCTCCGAAGCCTTCCCGGAGATGAAATGTACGGTGCTTGATCTTCCACATGTTGTTGCTGGACTGAAAGGGAATAAGAATCTGAGCTTTCTTGGAGGTGACATGTTTCAGGCTATCCCACATGCAGACATGGTTTTGCTCAAGTGGGTATTGCATGATTGGAACGATGAAGATAGTGTTAGAATACTGAAGAAATGCAAAGATGCAATTAGCAGCAGCAAGAACAAGGGTGGAAAGGTGATGATCATCGATATGATTTTGAACAATCATGGAGGAGGAATTAAAGCGATGGAAGATCAACTCTTCTATGACATGGCGATGATGGCTTATCTCAACGGGAAAGAGAGAACTGAGAAAGAATGGGCGAAGATCTTTTCTGATGCCGGATTTAGCAGCTACAAGATTGCTCTTGGATTAGGTGTTAGGTCTCTTATTGAGTTGTATCCTTGA

>CbuCOMT4

ATGGACGAAGAAGCACGAGCTCAAATAGATGTATGGAAATATGCTTACGGTTTTGACGCCATGAGAGTGGTGAAATGCGCCATAGAGCTAGGAATACCCGATGTCTTCGGAAGTCGTGGTAGCCCCATGACACTTTCTGAGCTATCCTCGGCTGTGGGTTGCCCTGAAAACTCTCTCTACCGCATCATGCGTTTCTTAACTCACAATGGCATTTTTAAAAAGAAAATAATTAGCCAAGATCCTCCATTGTTTCACTATTCTCAAACTCCACTTTCTCGTCTTCTCACAAGAGATAATATGGGCCTGTTTGTGCTCGTACAGGCCGGTCCGTCGGGAAAACAGTTTGGATTAACTGCAGAAGATTTGAGAGCTGGAAAAGGTTCTGGTCTGAAGCCTGCGGCTGATGAGATGACGATGTGGAGCTCAGGAGTAGTAGACGAAGCTTATGAAAAGCTGTTTAGGGATCATATGGCGAGCCATGGTAAGTTGGGGGCGTCGAAAGTTATCAATAACTGTCCTGAGGTTTTTGAAGGGATTGAGTCTTTGGTGGATGTTGGTGGTAACGACGGGACGGCCATCGGTATGTTTGTGAAGGCTTTTCCTTGGATTCGAGGGATTAATTTTGATCTTCCTCAAGTGGTACATGAGGCTCCAGCCATTGACGGGGTTCTGCATGTTGGTGGAGACATGTTTGAAAGCATTCCAAAAGCTGATGCAATTATGCTCATGTCAGTATTGCACGATTGGAGCGATGAAATGTGTATAGAAATCCTCAAGAAATGTAAAGAAGCTATACGGACAAAGACAGGGAAAGTGATCATTGTTGAAGTTGTGATTGATGAGGAAGGAGAAGAAGATGAGTACATGGGTGCACGTTTGCTGGTGGACATGATGATCATGATCGCAACTATCAATGGAAAGGAAAGGACTACTAAAGAATGGATAAGACTTCTCAATGCAAGTGGCTTTAGCAAATACACCATTAAGCATATGAGAGCTATCGAATCAATCATCGAGGCCTATCCTTAA

>CbuCOMT6

ATGGAAGTGATCAAACCAATCAAAGAAGTAGAAGAAGAAGGGCAGGCACAAGTAGATATATGGGAGTATATATTTGCTTTCATTCCAATGGCAGTAGTAAAATGCGCCATTGAACTCCAAATCCCCGACGTCTTAGAAAGCCACGGCGGAGCCATGACACTACCGGAGCTATCCGCCGCTCTTGGCTGCTCCCCTTCTATCCTCAGTCGCATAATGAGGTACTTAACCCACCGCGGTATCTTCAAGCAGAAGCTCACAAGCCAAATTTGCTACACCCAAACACCCCTTTCTCGTCTCCTCATGAAAAATGGAGCCAATACCATGGCTGCTCTTGTTTTGCTCGAAAGTAGCCCTGTGATGCTTGCTCCATGGCACAATCTGAGGACACGAGCACTAACCAATGGGGATTCAGCATTTGAGGCTGCACATGGGGGAGATGTATGGGATTTTGCTACTGAAAATCCTGCCCACAGTAAGCTAATTAATGATGCAATGGCTTGCCATGCAAAGCGGGCTATTCCAACTATCGTTAATCGTTATCCTGAGGTGTTTAAGGAGATTAGCTCATTGGTTGATGTTGGTGGTGGTAATGGGACAGCTCTTCGTACGTTGGTGAAGTATTGTCCATGGATTCATGGGATTAACTTCGATCTCCCACATGTGGTTGCCGTCGCCCCGTCGTGTGATGGTGTTGAGCATGTTGGTGGAAATATGTTTGAAATGGTTCCTAAGGCTGATGCTGCTTTTCTTATGTGGGTGTTACATGATTGGAGTGACAATGAATGTATTCAGATATTGACAAAATGTCGTGAAGCTATTCCCAAGGACACCGGAAAAGTGATCATCGCAGAGGCAATTATTGAAGAGGGAGAAGAGGACAAGTTTATTGATGTTCGTCTGGCTTTGGACATGGTTATGCTGGCTCACACGGAGAAAGGAAAAGAAAGGACTATTAAAGAATGA

>CbuCOMT5

ATGGACGTAACTAAATCACTAAAAGAAGTAGATGAAGAAGTGCAGGCACAAGTAGATATATGGCAATATATATTTGGCTTAGCTCCAATGGCAGTAGTAAAATGCGCCGTTGAACTCCAAATCCCTGATGTCTTAGAAAGTCATGGCGGAGCCATGACATTGCCGGAGCTATCCGCCGCTCTTGGTTGCACCCCTTCTGTGCTCAGCCGCATAATGAGGTACCTAATCCACCGTGGAATCTTTAAGCAGAAGACTACAAGCCAAGAATCACAAATTTGCTACATCCAAACGTCCCTTTCACGTCTCCTCATGAAAAATAGCATGGGTGCTTTTCTTCTAATGGAAAGCAACCCTGTGATGCTTGCTCCATGGCACAATCTCAGAGCATGCGCATTAGCCAAAGGGGCTTCAGCGTTTAAGGCTGCAAATGGAGCAGATTTATGGGATTATGGATCCGAAAATCCTGGCCATAGCAAGCTATTCAATGATGCAATGGCTTGCCATGCAAAGTTGGCTATTTCAAATATCGTTAATCGTTATCCTGAGGCGTTTAAGGGCATAAGATCTTTGGTGGATGTTGGTGGTGGTAATGGGACAGCTCTGCGTACATTGGTGAAGTCTTGTCCATGGATTCGTGGGATTAACTTCGACCTCCCACATGTTGTTTCCATCGCTCCACCGTGTGATGGTATTGAGCATGTTGGTGGGGACATGTTTGAGATGGTGCCCAAGGCTGATGCTGCTTTTCTCATGTGGGTACTGCATGATTGGAGTGATGATGAGTGCATCCAAATATTGAGAAATTGCCGAGAAGCCATTCCCAAGGACACAGGAAAAGTGATCATTGCAGAGGCAATGATCGAAGAAAGAGAAGAGGATAAGGTCACCGATGCTCGTTTGGCATTGGATATGGTGATTTTGGTTCACACAGAGAAAGGAAAAGAGAGGACTATTAAAGAATGGGAATATGTGGTTTATGCAGCTGGCTTCACTAAGTGTACTATAAAACATATTGAAGTAGTAAAATGCGCCATTAAACTCCAAATCCCCGATGTCTTAGAAAGCCATGGCGGAGCCATGACACTGCCGAAGCTATCTGCTGCCACTCTTGGCCGCTCCTCTTCTGTGCTCAGCCGCATAATGAGGCTGAAAATGGGGCGGATTTATGGGATTATGGATCCTAAAATGCTGGCCATAGCAAGCCATTCAATGATGCAATGGCTTGCCATGCTAAACTGGCTATTTCAAGGATCTTTAATTGTTATCCTGAGATGCATGTTTAAGGGGATAGGATCTTTGGTAGATGTTGGTGGTGGTAATGGGACGGTTCTTCATACGTTGGTGAAGTCTTGTCCTTGGATTCGTGGGATTAACTTTGACCTCCAACATGTTGTTTCTGCCGCCCCATCGTGTGATGGTATTGAGGACATGTTTGAGATGGTGCCTACGGCTGATGCTGTTTTTCTCATGGGGATTGGGTAG

>CbuCOMT9

ATGGTGGGCTCAGCAGTTGAGAGTTGGCTGCAAGTTGACGTCGTTCCATTTTTGGGAATACACCCTCCGACTACTGTTCTTCCAACAACTAACCCGGATGCAGCCGATATGATAGATAGAATTCTCCGGCTGCTGGCGTCGCATTCCGTTCTCATTTGCAGCCTGAAACAGCTGCCGGACGGCGGCGTTGAGCGGCGCTACTCTCTTGCTCCGGCATGTGTAAATAGGGTCTTGATGGAACCTGGGTATCATCTAAATGATGCAATTCTTGAGGGAGGAATCTCATTCGATAGAGTATATGGTATGAATGCATTCGAATACTTGGCTAAAGATCCGAGATTCAACCGGGTTTTTAACCGAGCTATGCACGAACCATCCACCATAATTATGACAAAAATTCTTGAAAAATACAAAGGATTCGAGGGCCTGAAATCCCTAGTGGATGTTGGTGGTGGAATTGGATCATCACTTAACATGATCATTTCCGAGTATCCATCAATCAAGGGCATTAATTTTGATTTGCCCCATGTTATTCAAGTTGCTCCTCCTCATCCAGGAGTGGAGCACAATAGTGGTGACATGTTTATTAGCGTGCCTAAAGCCGATGCCATTTTTATGAAGTGGGTTTGCCACGGTTGGAGAGATTCACATTGTGAAAAGCTGTTGAAGAATTGCTACGAAGCGCTGCCCGAAAATGGAAAAGTGATTATTGCTGAAGTTATTGTGCCCGACAACCCAAATACCGGGCAGAGTTCTTCATGGGCAGCCCAAGGTGACATGATTATGTTAGCTTATACTTCAGGTGGAAAGGAGAGGTCAGAAAGGGAATTTGAGGCCTTAGCTGAAAAAGCTGGATTCAAACACCTTATCAAAGTTTGCAGTGCTTATAGTAATTGGTTAATGGAATTTCATAAATGA

>CbuCOMT10

ATGGATAATTATAAGCCCGACGAAGAAGCCTGCTTATTCGCCTTCCAGCTAGTATCTGGTTCGGCGCTTCCAATGGTACTGCAAACCGCCATAGAACTCGATCTTCTGGAACTTATCAAAAAATCAGGGCCAGAAGCTTCAGCTTCTGCTTCTGAACTTGCGGCTCAGCTTCCAACAAGTAACCCTGATGCAGCCCATATGATAGATAGAATTCTCCGGCTGCTGGCGGTGCATTCCGTTCTCATTTGCAGCCTGAAAAAGCTGCCGGACGGCGGCGTTGAGCGGCGCTACTCCCTTGCACCGGTGTGTAAGTTCTTGACTAGAAACGAGGATGGAGTTTCTGTGAGCCCTCTTTGTCTCTTGATTCAAGATAGGGTGTTGGTGGAACCTCGGTATCTTCTAAAGGATGCAATTCTTGAGGGAGGAATCTCTTTCGATAGAGCATATGGTATGAATGCATTCGAATACTTGGCTAAGGATCCAAGATTCAACCGGGTTTTTAACCGAGCCATGCATGAACCATCCACCATAATTATGGCGAAAATTCTTGAAAAATACAAAGGATTTGAGGGTCTGAAATCCCTAGTTGATGTTGGTGGTGGAATTGGAGCATCACTTAACATGATCATTTCCAAGTATCCATCAATCAAGGGCATTAATTTTGACTTGCCCCATGTTATTCAAGATGCTCCACCTTTTCCAGGAGTGGAGCACATTAGAGGTGACATGTTTGTTAGCGTGCCTAAAGCCGATGCCATCCTTTTGAAGTGGGTTTGCCACGATTGGAGCGATTCAAGTTGCGAAAAGCTGTTGAAGAATTGCTACGAAGCGCTGCCCGAAAATGGAAAAGTCATTGTTGCCGATGCTATTCTGCCCGAGGACCCAAATAGCGGGCAGAGTTTTTTTTGGGCAACCCAAATTGACGTGATTATGTTAGCTTATAATCCAGGTGGAAAAGAGCGGTCAGAGAGGGAATTTGAGGCCTTGGCCAAAAAAGCCGGATTCAAACACCTTATCAAAGTTTGCAGTGCTTATGCTGATTGGGTTATGGAATTTCATAAATGA

>CbuCOMT7

ATGATCTTCTTCTTTGGAAGGTATCATCTAAAGGATGCAATTCTTGAGGGAGGAACCCCATTCAATAGAGCACATGAATACAAAGGATTTGAGGGTCTGAAATCCCTAGTGGATGTTGGTGGTGGAATTGGAACATCACTTAACATGATCATTTCCAAGTATCCATCAATCAAGGGCATTAATTTTGACTTGCCCCATGTTATTCAAGATGCTCCATCTTATTCAGGAGTGGAGCACACTAGTGGCGATATGTTTGTTAGCGTGCCCAAGGCCGATGCCATTTTTATGAAGTGGATATGCCACGATTGGGGCGATTCACATTGCGAAAAGCTGTTGAAGAATCGCTATGAATCGCTGCCTGAAAATGGAAAAGTGATTATTGGTGAAGCTATTCGATCTGAGGACCCAAACAGTTCTTTACAGTCAGCTCTGTCTGATGTGATTATGTTAGCTTTTAATCCATATGGAAAACAGCGATCAGAAAGGGAATTTGAGGCCTTAGCTAAAAAAGCTGGATTCAAACACCTTATCAAAGTCTGCAGGGCTTCTCATATTTGGATTATAGAATTTCATAAATGA

>CbuCOMT8

ATGGATAATCAGTCCGATGAAGAAGCCTGCTTATTCGCCTTGCAGCTAGCAACCGGTTCAGTGCTTCCAATGGTTCTAAAAACCGCCATAGAACTCGATCTCCTGGAACTTATCAAAAAAGCTGGGCCAGAAGCTTCAGCTTCTGCTTCTGAACTTGTGGCTCAGCTTCCAACAAACAACCCTGATGCAGCCAATATGATAGATAGAATTCTCAGGCTGCTGGCGGCACATTCCGTTCTTGTTTGCAGCCTGAAACCGCTGCCGGACGGCGGCGTTGAGCGGCGCTACTCCCTTGCGCCGGTGTGTAAGTTCTTGACTAGAAATGAGGATGGAGTTTCTGTGGGCCCTACTTGTCTCATGATCCAAGATAAGGTGTTGATGGAACCTTGGTATCATCTAAAGGATGCAATTATTGAGGGAGGAATTCCATTCAATAGAGCATATGGTATGAATGCATTCGAATACCCGGCTAAGGATCCGAGATTTAACCGGGTTTTTAACCAAGCCATGTATGAACAATCCACCATATTTATGAAGAAAATTCTTGAAGAATACAAAGGATTTGAGGGTCTGAAATCCCTAGTGGATGTTGGTGGTGGAATTGGAGCATCACTTAAGATGATCATCTCCAAGTATCCATCAATTAAGGGCATTAATTTTGATTTGCCCCATGTTATTCAAAATGCTCCATCTTATCCAGGAGTGGAGCACATTAGTGGTAACATGTTTGTTAGCGTGCCTCAAGCCGATGCCATTTTTATGAAGAACTAA

>CbuCOMT11

ATGGATAATCAGTCCGATGAAGAAGCCTGCTTATTCGCCTTGCAGCTAGCAACCGGTTCAGTGCTTCCAATGGTTCTAAAAACCGCCATAGAACTCGATCTCCTGGAACTTATCAAAAAAGCTGGGCCAGAAGCTTCAGCTTCTGCTTCTGAACTTGTGGCTCAGCTTCCAACAAACAACCCTGATGCAGCCAATATGATAGATAGAATTCTCAGGCTGCTGGCGGCACATTCCGTTCTTGTTTGCAGCCTGAAACCGCTGCCGGACGGCGGCGTTGAGCGGCGCTACTCCCTTGCGCCGGTGTGTAAGTTCTTGACTAGAAATGAGGATGGAGTTTCTGTGGGCCCTACTTGTCTCATGATCCAAGATAAGGTGTTGATGGAACCTTGGTATCATCTAAAGGATGCAATTCTTGAGGGAGGAATTCCATTCAATAGAGCATATGGCATGAATGCATTCGAATACCCGGCTAAGGATCCGAGATTTAACCGGGTTTTTAACCAAGCCATGTATGAACAATCCACCATATTTATGAAGAAAATTCTTGAAGAATACAAAGGATTTGAGGGTCTGAAATCCCTAGTGGATGTTGGTGGTGGAATTGGAGCATCACTTAAGATGATCATCTCCAAGTATCCATCAATTAAGGGCATTAATTTTGATTTGCCCCATGTTATTCAAAATGCTCCATCTTATCCAGGAGTGGAGCACATTAGTGGTAACATGTTTGTTAGCGTGCCTCAAGCCGATGCCATTTTTATGAAGTGGATTTGCCATGATTGGAGCGATTCACATTGCGAAAAGCTCTTGAAAAACTGCTTCGAAGCGCTGCCCGAAAATGGAAAAGTGATTATTGCTGAGACTATTCTGCCCGACGACCCAAATAGCGGGCCGAGTTCTTTACGGGCAGCCCAAGCTGATGTGATTATGTTGGCTTATAATCCAGGTGGAAAGGAGAGGTCAGAAAGGGAATTTGAGGCCTTAGCTGAAAAAGCTGGATTCAAACACCTTATCAAAGTTTGCAGTGCTTTTAATATTTGGATTATGGAATTTCATAAATAA

>CbuCOMT16

ATGGTGCTAGACAAAGAAGCCCAAGCTCGTGCAGATGTCTGGAAATACGCTTTCGGATCTATCAACTGCAGAGTAATGATAGTCGTCGTCCAGCTCCAAATACCCGATATAATGAAAAAACACGGCGGTGCTATCTCACTCTCCGACCTCTCCGCCGCCGTGGGTGTCCCTGCCGATAACCTCTACCGCATAATGAGATTCTCAATCCATCACGGCATGTTCAAGAAAACAGAGGCCCCACAGAGAAAAGTCTCAGACGAAGTCGTATACTACGCTCACACGCCGCTTTCTCTTCTTTTAACAATTGACAACGTCGGACCCTTCATTCTGCTGCAGGGGGCCGGTCCCCATGGAAACTTCGGGGGATTAACTGTGGCTGCTTTGAAAAGCGGAAATCGGCCCGATTTTAAGAATCTCAACGGCAACGGCAATGGAAACGGCAACGGCAACGGAAACGGCAACGGCAATTGGGACGATCCATTCTATACGACAAAGGAGTTTACGGACGCTATGGCGTGCCATGCTAGGGTGGCAACATCGGCGATTATTGAGAACTGTCCGGAAGCTTTCCGAGGAATTAGGACGTTGGTGGATGTAGGCGGCCGCCATGGGATGGCCCTTAGTATGTTGATAAAGGGATTTCCATGGATTAAGGGGATTGCTTTTGATCTTCCTGAGGTTGTGGCTAAGGCTCGTCCTGTTGATGGGATTCAGTTCGTTGGAGGGAGTATGTTTGAAACTATCCCAAAAGCTGATGCAATTATGCTCATGTGGATATTGCACGATTGGAGCGACGAAACGTGCATAGACATCCTCAAGAAATGCAAAGAAGCCGTTCCGGCAGACACCGGAAAAGTGATCATCGCAGAAGCTGTCATCAACGAAGATGGCGAAGAAGATGAGTACACAGGTGCCCATTTGTCGCTGGATATGATAATGATGGACCAACTGATCGAAGGCAAAGAGAGGACATACAAAGAATGGGCACATCTTATCAAGGCAGCTGGCTTTAGCAGACACAACGTTAAAAATATGAAAACTCTTGTCTCTCTAATTGAGGCCCATCCCTAA

>CbuCOMT14

ATGGATAATAAGTCAGATGAAGAAGCCTGCGTATTCGCCTTCCAGCTAGCAGCCGGTTCAGTGCTTCCAATGGCACTATACACCGCCATAGAACTCGATCTTCTGGAACTGATCAAAAAAGCTGGGCCAGAAGCTTCAGCTTCTGCTTCTGAACTTGCTGCCCAGCATCCAACAACTAACCCTGATGCAGCCGATATGATAGATAGTATTCTCCGGCTGCTGGCGTCGCATTCCGTTCTCATTTGCAGCCTGAAACAGCTGGCGGACGGCGGCGTTGAGCGGCGCTACTCTCTTGCTCCGGTGTGTAAGTTCTTGACTAGAAATGAGGATGGAGTGTCCGTGGGCCCTCTTTGTCTCTTGCTTCAGGATAATATAACCTGGGTACGAGTGGAGCACGTTAGTGGTGACATGTTTGTTAGCATGACTAAAGCCGATGCCATTTTTATGAAGTGGGTTTGCCACGGTTGGAGAGATTCACATTGTGAAAAGCTGTTGAAGAATTGCTACGAAGCGCTGCCCGAAAATGGAAAAGTGATTATCGCTGAAGTGATTGTGCCCGACAACCCAAATGGCGGGCAGAGTTCTTCATGGGCAGCCCAAGGTGACATGACTATGTTAGCTTATACTTCAGGTGGTGGAAAGGAGAGGTCAGAGAGGGAATTTGAGGCCTTAGCTAAAATAGTTGGATTCAAACAGCTTATCAAAGTTTGCAGTGCTTATAGTAATTGGATTATGGAATTTCATAAATGA
