# Supplementary material for: Genome-wide analysis of the Catalpa bungei caffeic acid O-methyltransferase (COMT) gene family: identification and expression profiles in normal, tension, and opposite wood
Source: PeerJ. 2019 Mar 14;7:e6520. doi: 10.7717/peerj.6520 (PMC6421059; doi:10.7717/peerj.6520)
Supplement: File S5 [file peerj-07-6520-s007.docx]

>CbuCOMT16

ACAGGGAATCATTAAACACCCTTACTACATAACCACACATACACCGCAAAGATTACTTGCGCATAATAAGTTAAATAAGAGCATAAGAGACATCATTGTTAGGGATGGGCCTCAATTAGAGAGACAAGAGTTTTCATATTTTTAACGTTGTGTCTGCTAAAGCCAGCTGCCTTGATAAGATGTGCCCATTCTTTGTATGTCCTCTCTTTGCCTTCGATCAGTTGGTCCATCATTATCATATCCAGCGACAAATGGGCACCTGTGTACTCATCTTCTTCGCCATCTTCGTTGATGACAGCTTCTGCGATGATCACTTTTCCGGTGTCTGCCGGAACGGCTTCTTTGCATTTCTTGAGGATGTCTATGCACGTTTCGTCGCTCCAATCGTGCAATATCCACTGTCATAAAATAGTATGAGAAAACACTTTTACTAATGAAAAATTTGTGATGAAAAGGGTTTCCTCGTAAATAAACGATATTTGTGATGAAACAGAACTCTAGCAAATTTAGATATGGGCATGATACCTTGTTGTGAATTTGTGATAGATATTATGTCAATTTAAAATATTAGTTTTTCTTTTCTTTTCCTTTTTTTTTTATTTGAAGGCTAAAATATTAATTTTTCCCACTAAGGCATATGTTGTGACAAAATAAAAATTATTGTCGTCTAAAACATATTATTATCACTTAATAGATGTTATTAATGATAATTTTTTATCACTTATTAGTTATATTTTGGTAAAGTAACTCAAAAATAAATTACATAGTACTTAATTTATTAATATTGGTCGTTAACGCGTACCATGAGCATAATTGCATCAGCTTTTGGGATAGTTTCAAACATACTCCCTCCAACGAACTGAATCCCATCAACAGGACGAGCCTTAGCCACAACCTCAGGAAGATCAAAAGCAATCCCCTTAATCCATGGAAATCCCTTTATCAACATACTAAGGGCCATCCCATGGCGGCCGCCTACATCCACCAACGTCCTAATTCCTCGGAAAGCTTCCGGACAGTTCTCAATAATCGCCGATGTTGCCACCCTAGCATGGCACGCCATAGCGTCCGTAAACTCCTTTGTCGTATAGAATGGATCGTCCCAATTGCCGTTGCCGTTTCCGTTGCCGTTGCCGTTTCCATTGCCGTTGCCGTTGAGATTCTTAAAATCGGGCCGATTTCCGCTTTTCAAAGCAGCCACAGTTAATCCCCCGAAGTTTCCATGGGGACCGGCCCCCTGCAGCAGAATGAAGGGTCCGACGTTGTCAATTGTTAAAAGAAGAGAAAGCGGCGTGTGAGCGTAGTATACGACTTCGTCTGAGACTTTTCTCTGTGGGGCCTCTGTTTTCTTGAACATGCCGTGATGGATTGAGAATCTCATTATGCGGTAGAGGTTATCGGCAGGGACACCCACGGCGGCGGAGAGGTCGGAGAGTGAGATAGCACCGCCGTGTTTTTTCATTATATCGGGTATTTGGAGCTGGACGACGACTATCATTACTCTGCAGTTGATAGATCCGAAAGCGTATTTCCAGACATCTGCACGAGCTTGGGCTTCTTTGTCTAGCACCATTTTTTTTTTTTAGCAACTTTATTGAGCTTATCTGTAAATTGGGTTACTCTATAAGTTTTGAGAAAGAAGAGCATGC

>CbuCOMT17

GGGAACCATCACTCTCCTATTTCCCCCAACTCTCCCTCGAGAAGTAAAGAAACTAAACTAATGGACGAAGAAGCTCGAGCTAAAGTAGAAATTTGGCAATACGTTTTAGGTTTCATTACAACGAAAGTGGTGAAATGGACCATAGATCTTAAATTGCCCGACGCCGTCGAAAGTCACGGCGGCCCCATCACTCTCTCTCAACTATCCGCCGCCGTGGGCTGCCCCACCGCCGCTCTCCACCGCATCATGCGCTTCTTGACTCACAAAGGAATCTTCAAAAAGCAGATTAACTTAAGCAAAAGCCCCGATCCTGAATCAATTTACTATTGCCAAACGCCCCTTTCCCGCCTCCTCGCAAGAGATAAATTGGCCCTTTATTTACTTATCCAGGCGGGTCCTTCAGAGGAGCTGGACATGGATTTCAGTGCAGAATATATGAAAGCTGGAAGAGGGTCTGGTTCGGATAATCTTTCTCTTTCTGAGGATACAATCTGGGATGCAAAAGTTGATGCAACTCATGATAAGCTGTTTCGTGAGTTTTTAGCTTGTCATGCTAAAATTGTGACCACGTCGCTTATCGATTATTGTCAGGAGGTTTTTGAAGGAGTTGGGTCTTTGGTGGATGTTGGTGGTCATGAAGGGATGGCTATCGGGATGCTGGTTAAGGCTTTTCCTTGGATTCGGGGGACTAATTTTGATCTCCCAGATGTGATCGCTAGGGCTTCCCCCATTGATGGGGTGGAGCATGTTGGAGGAAACATGTTTGAAAGTATTCCAAAAGCTGATGCAGTTATGCTCATGGTATGTGTTTTTGTTCAGATGGGATCTCCTCCTGTTGCACTTTTTTGGTCCACCAATAATACGGTGCCACATGTATTGTGGAACATTTTTCTTTTAAATTTTTACAAACGACATTTTCAATGTCTACTATTCCACAATACTGCATGTGGCGGTTTAATATTAGTGGAACAGAAAAGTGGAATTGAAAATGCCATCCGATTTTTGTGTGTGCATATGTTCATTTCATAGTAAAAGTTTACAACGAATTAAGTCATCATTACTGGGTGTCGTGTATGGGAATGATGATCATGTGATTCTTTATAAATTTTCGTTCAAGGAACATATCATGATACCAAATTTTATCCCATATAGGAGGAATCAAAAATCTTGAGAAATAGTTCCTAAACTGCTATGTCTTACTCATTAAGGTGCTTTTTATAAGGATATAAAAAAAAAAAAAATGTATAGAAAATACCTTGACTCAATCTATTGGATATTTCATATATAACGACAGTTTTAGTATTATGTATGTGAGAATGACGATCATGTGCCTCTTTTCAAAGCCCCTTGTCTTATAAGGTTTTTCACTTTTTTGACGTGTTGAGACATGTTTTTTAAAATAAGAACGAAATTCCCCTCCACAAGGGGAATGTCAACGGAACATTACTTCAGGAGTTGACTAACAAGCCATGTCTAATGATACATTTAAAGATAATTAAACCAAACAACGATCACATACAAAAATGTGCTTCTTTTTTTTTTTTGAAGGTGCTATTTGTTTTTCTTTTAAAAGATGCATTTTTTTCATCACATATCATTTCTTGAATTCTTTTCATGGCAGTCGGTATTGCATGACTGGAGCGACGACATATGCTTAGACATCCTCAAGAAATGCAAAGAAGCTATTCCAGTAGACACAGGGAAAGTGATAATTGTTGAAGTTGTAATAGACGAAGAAGGACGCGATGAGTATACGGGTACTCGTTTGGCAATGGATCTGGCAATCATGACCGCAACAATAAAAGGGAAGGAGAGAACTAATAAGGAATGGGCACAACTTCTAAATGCCGCAGGCTTTAGCAGACACATTATCAAACACATGAAAGCTATTGAATCTGTCATTGAAGCCTATCCATAG

>CbuCOMT18

GGGAACCATCACTCTGCTATTTCCCCCAACTCTCCCTCGAGAAGTAAAGAAACTAAACTAATGGACGAAGAAGCTCGAGCTAAAGTAGAAATTTGGCAATACGCTTTAGGTTTCATTACAATGAAAGTGGTGAAATGGGCCATAGATCTTAAATTGCCCGACGCCGTCGAAAGTCACGGCGGCCCCATCACTCTCTCTCAACTATCCGCCGCCGTGGGCTGCCCCACCGCCGCTCTCCACCGCATCATGCGCTTCTTGACTCACAAAGGAATCTTCAAAAAGCAGATTAACTTAAGCAAAAGCCCCGATCCTGAATCAATTTACTATTGCCAAACGCCCCTTTCCCGCCTCCTCGCAAGAGATAAATTGGCCCCTTATGTACTTCTCCAGGCTGGTCCTCCCAGGGAGCTGGACATGGATTTCAGTGCAGAAGATTTGAAAGCTGGAAGAGGGTCTGGTTTGGATAATCTTTCTCTTTCTGAGGATAAAATCTGGGATGCAAAAGCTGATGCAACTCATCATAAGCTGTTTCTTGAGTTTTTAGCTTGTCATGCTAAAATTGTGACCACGTCGCTTATCGATTATTGTCAGGAGGTTTTTGAAGGAGTTGGGTCTTTGGTGGATGTTGGTGGTCATGAAGGGATGGCTATCGGGATGCTGGTTAAGGCTTTTCCTTGGATTCGGGGGACTAATTTTGATCTCCCAGATGTGATCGCTAGGGCTTCCCCCATTGATGGGGTGGAGCATGTTGGAGGAAACATGTTTGAAAGTATTCCAAAAGCTGATGCAGTTATGCTCATGGTATGTGTTTTTGTTCAGATGTGTTTTTGAGAAATAGTTCCTAAACTGCTGTCTCACTCATTAAGGTGCTTTTTCATAAGGATATAACAAAATTAGAAAGAAAAAAAAATTATGTATAGAAAATACCTTGACTCAATCTTGGATATATATTTCATATATGACGACAGTTTTGGTATCATATATGTGAGAATGACAATCATGTGCCTCTTTTCAAAGTTCCTTACTTTTTTGATGTGTTGAGACATCTTTTTTAAAATAAGAACGAAATTTCCCTCCCGGCAAGGGAATGTCAACAGAACAATACTTAAAGAGCTGACTAGCAAACCAAACAACAATCCCATACAAAAATGTGCTATTTTTTTTCTTTTTCTTTTTCTTTCTTTTAAATGATGCATATTTTTCATCACATATCATTTCTTGAATTCTTTTCATGGCAGTCGGTATTGCATGACTGGAGCGACGACATATGCATAGACATCCTCAAGAAATGCAAAGAAGCTATTCCAACAGACAAAGGGAAAGTGATAATTGTTGAAGTTGTAATAGACGAAGAAGGAGGGGATGAGTATACGAGTGCTCGTTTGGCAATGGATATGACAATCATGACCGTAACAATAAATGGGAAGGAGAGAACTTATAAGGAATGGGCACAGCTCCTAAATGCCGCAGGATTCAACAGACACGCTATCAAACACATGAAAACTATTGACTCTGTCATCGAAGCTTATCCGTANNNNNNNNNNNNNNNNNNNNNNNNNNNNNNNNNNNNNNNNNNNNNNNNNNNNNNNNNNNNNNNNNNNNNNNNNNNNNNNNNNNNNNNNNNNNNNNNNNNNNNNNNNNNNNNNNNNNNNNNNNNNNNNNNNNNNNNNNNNNNNNNNNNNNNNNNNNNNNNNNNNNNNNNNNNNNNNNNNNNNNNNNNNNNNNNNNNNNNNNNNNNNNNNNNNNNNNNNNNNNNNNNNNNNNNNNNNNNNNNNNNNNNNNNNNNNNNNNNNNNNNNNNNNNNNNNNNNNNNNNNNNNNNNNNNNNNNNNNNNNNNNNNNNNNNNNNNNNNNNNNNNNNNNNNNNNNNNNNNNNNNNNNNNNNNNNNNNNNNNNNNNNNNNNNNNNNNNNNNNNNNNNNNNNNNNNNNNNNNNNNNNNNNNNNNNNNNNNNNNNNNNNNNNNNNNNNNNNNNNNNNNNNNNNNNNNNNNNNNNNNNNNNNNNNNNNNNNNNNNNNNNNNNNNNNNNNNNNNNNNNNNNNNNNNNNNNNNNNNNNNNNNNNNNNNNNNNNNNNNNNNNNNNNNNNNNNNNNNNNNNNNNNNNNNNNNNNNNNNNNNNNNNNNNNNNNNNNNNNNNNNNNNNNNNNNNNNNNNNNNNNNNNNNNNNNNNNNNNNNNNNNNNNNNNNNNNNNNNNNNNNNNNNNNNNNNNNNNNNNNNNNNNNNNNNNNNNNNNNNNNNNNNNNNNNNNNNNNNNNNNNNNNNNNNNNNNNNNNNNNNNNNNNNNNNNNNNNNNNNNNNNNNNNNNNNNNNNNNNNNNNNNNNNNNNNNNNNNNNNNNNNNNNNNNNNNNNNNNNNNNNNNNNNNNNNNNNNNTTAATATCATTGAAATTAAATATTTTTTAATTTCCTCCAATGCCTAGTCATCTATGGGCTTTTAAACAAAGATGCCACGTAGACTTCCATGTAAACAGGGAGGAAGAATGCCACATGCCGGTGACGGCTCTGGCGAAAAACAAACATAAAGTGTTGATCAGAGCAAATATACCATACATTTTATTAATAGTTAAATTAGAGGGCAAAAAGTGA

>CbuCOMT19

AATTCATCAGAATCATTATCAAGCATCAATTACATCTTTTTATTAACTGTGACAACAACAATGGCTGCAATACCAGGTGTTCTAGGCACAAGCAAGATGCTTTCCACAATTTATTACAACAATCCCATGTACATATGGATCATGTGAATCCTTGAACTAATCAAGGATACAACTCAATAAGAGACCTAACACCTAATCCAAGAGCAATCTTGTAGCTGCTAAATCCGGCATCAGAAAAGATCTTCGCCCATTCTTTCTCAGTTCTCTCTTTCCCGTTGAGATAAGCCATCATCGCCATGTCATAGAAGAGTTGATCTTCCATCGCTTTAATTCCTCCTCCATGATTGTTCAAAATCATATCGATGATCATCACCTTTCCACCCTTGTTCTTGCTGCTGCTAATTGCATCTTTGCATTTCTTCAGTATTCTAACACTATCTTCATCGTTCCAATCATGCAATACCCACTGCGTGGCACCACACGAAGGTTGTTTAGAGTATATGTGGTTAGGGGATATTATGCTATTATAGCTAGATGTGTGTATATTCTTGCTAATATTTTGAGGGGGCGTTTGATGGAGCGAGCGTCTAATCTATGTAAAGATAGACCCATCGAGATAAGATTGGTTTGCCAATTTCAATTTTATGTTTGATAGGGAAGATTGAAGGTGAGATTAGTAATGACTGTGATGTTTGATGGAATGGAGGAGTGGTTACACATTTGCCCTTCTCCTCCAATTTCGTTTTATCCCAAAACAAAAAAGTAATTGGCCTTGTAATTTCTATGGAGCGATTATTAATCATTGGGCAAGGTCCAAGACTATAATTACTTATAATCCAATCTACAGTGCGGGCTATGTCTTGATTTCGCATTGGCCCGAATACCCGAATCCATTCAATCAGACACGCCCTCATAAGAGAATTGTAGTTAACTCGATAGATAACACCTAATTAATAGAAAATTCCCTATAAAAAATTCAATGGTTGACTAGATCTCATTCATTCCCAGAAAATTAACTCAATTGCCCTAACTTCTACTTATTTGTCAAGATATCCTCGTATATAAATCCTGCAAATATAGACCCATATTTTTTGTTATTTTTCGGCCTGGAATCCTATTTAGCTGAAATTAATTGCACTACTCCCTAATAATTTTTTGTTCCTCAATGGAAATTACGACAAATCACAAACTTCTGTTCATACCTTGAGCAAAACCATGTCTGCATGTGGGATAGCCTGAAACATGTCACCTCCAAGAAAGCTCAGATTCTTATTCCCTTTCAGTCCAGCAACAACATGTGGAAGATCAAGCACCGTACATTTCATCTCCGGGAAGGCTTCGGAGATTGCCCCAGCCGTTGCTCCGGTGCCACCACCGACATCCACCAAAGACTCAAACCCTCCAAACAACTGTTTGGAATTTCTAAGTACTACAAGATTCACGAGCCCTGCATCACTAGCCATGGCTTCATTGAACAAGTGGTTAAGCCTTGGGACCCGTTCCGCCTGCTCCCAAAATGTCCGGCCATGAGCCATTTCAAACGGCGTCTGGTGGTGGTCGTTGACAAGCCATTTGCTCAGATAATGCCATGGCTCGATCATAATTGGGTCCGCTATGACTTGTAAGAAAGGTATGATGCTCAAGGGCTCATCTTTAAGGAGGAGACGAGAGGCTGGTGTGAGCCAGTAGCCCTCTTTTGGATTGTTATCAGAGATGTTGACTTCGATGAAGAAGTTTGAATGAACTAATAGGCGCATTAAGCGAGAGACATATTGGGATTTGGCTTGGCT

>CbuCOMT20

ACGAGCAATATCATAGTCCTCACAACTTTTACATAGATAAATAACATTTAACATACAACAAAAGGAAATATGATTGCTTATTGTAAAAGACGTTTTTTACAACAACTCTAAAAGCCACACAATTGGCATTCAATTAAGTTTCATACTTATGAATATGCCTCAATAACAGATATTATTTCACCTTCAATATGTTTTATAGTATACTTAGTGAAGCCAGCTGCATAAACCACATATTCCCATTCTTTAATAGTCCTCTCTTTTCCTTTCTCTGTGTGAACCAGAATTGCCATATCCAATGCCAAATGAGCATCAGTGACCTTATCCTCTTCTCTTTCTTCGACCACTACCTCTGCAATGATCACTTTTCCTGTATCCGTGGGAATGGCTTCTCGGCAATTTCTCAATATCTGGATACATTCATCATCACTCCAATCATGTAGTACCAACTGTATTATAAATTGAGCATATGTTAGTGAAAGTACCCAAAATGTCCATTTAAAGTTAGGTCTAAAATCTAACGAGACAAGATTCGTTAAATTGGACGCAAAAATGGAGCCAAATCGATTTGACCCGTCTCTTATTATAATGTATATGGATGTTCTATTTCAAAATGTATTTCATCTCATACTCCATTAGTTTCTTGGATACATTACAAACATAGTAAATATTTTCCTCGTAAAAGTATTTGATGATAGATGCTCTAAATTTATCATGTAACGCTAAATTAAATACATAAAATAAACATATTTAGGTACACAATTTTAGAATATCTACTATTTCTCTTAGCTGTCCCAAGATGCATTATCACTTATTTGAGCTCAAAGGGTTTTCCGGCTAAGCAAACACGGTGATTCTGAAAATAATTTTTTAATTTCAAGTTCTAGGTGATCACCTAGTTAGATATATCTACTCGATCCAATCTCCAAATCAAATGGTCAATTTCAAATCTAAATCGCACATGAGGATGATCATATGAATTAAAATTAACCGGAGTAAACACGCACATACCATGAGAAAAGCAGCATCAGCCTTGGGCACCATCTCAAACATGTCCCCACCAACATGCCCAATACCATCACACGGTGGAGCGATGGAAACAACATGTGGGAGGTCGAAGTTAATCCCACGAATCCATGGACAAGACTTCACCAATGTACGCAGAGCCGTCCCATTACCACCACCAACATCCAACAAAGATCCTATGCCCTTAAACGCCTCAGGATAATGATTAACAATATTTGAAATAGCCAACTTCGCATGGCAAGCCATTCCATCATTGAATAGTTTGCTATAGCCAGGATTTTCGGATCCATAATCCCAGAAATCCGCCCCATTTGCAGCCTTAAACGCTGAAGCCCCATTGGTTAATGCGCGTGTTTTCAGATTGTGCCATGGAGCAAGCATCACAGGGTTGCTTTCCATTAGAATAAAAGCAGCCATGCTATTTTTCAAAAGGAGACGAGAAAGGGGTGTTTGGATGTAGCAAATTTGTGATTCTTGGCTTGTGGGCTTCTGCTTAAAGATGCCGCGGTGGATTAAGTACCTCATTATGCGGCTGAGCACAGAAGGGGAGCAGCCAAGAGCGGCAGATAGCTCCGGTAATGTCATGGCTCCGCCGTGGCTTTCTAAGACATCGGGGATTTGGAGTTCAATGGCACATTTTACTACTGCCATTGGAGCGAAGCCAAATATATACTGCCATATATCTACTTGTGCCTGCGCTTCTTCATCCACTTCTTTGAGTGATTTAGTTAAGTCCATCTTTATTTCTCATTTTTGATGTACAAGAAACTAGGCAATTCCACACAATTATATAGTTGCTCCTTTGGTTTCAAACTAAATTGGAGTTGGGCTTTTG

>CbuCOMT21

ATTCAACATAGATCACTGCTTATCGTAGAAGATGCATTACAAGATTCTAATTAAAATCATAAATCAAGTTTCACAATACTCATGGATAGACCTCAATAATATATATAATTTCACCTTCAATACGTTTCACGGTAAACTTGGTAAAACCAGCTGCATAAACCACATATTCTCATTCTTTAACAGTTCTTTCTTTTCCTTTCTCTGTGTGAGCCAGCATAACCATGTCCAAAGCCAGACGAACATCAATAAACTTGTCCTCTTCTCCTTCTTCAATAATTGCCTCTGCGATGATCACTTTTCCAGTGTCCTTGGGAATAGCTTCATGACATTTTGTCAATATCTGAATACATTCATTGTCACTCCAATCATGCAACACCCACTGCATAATTAGTAGGCAATAGTGTTACATAGCGATAATGACATGAGACAAGTATTACTACCAAAATGACCATTTAAAATTAAGTCAAATGTCAATAAGACATCATCTAGGAAATTAGGTTCTCCGACTGCTTTACATCTCAACTCTTTCTCTATCCTAATGATCCTAGTAATTTTTTTTTTTTTTTTTTGAAGTCTTGGTTCACTCATATCAACTAGATAGATAGATCAATCTAACCCAGAGACCTACCCAATCAAAGACCCAATTCAAGGATCATTTCATAACTAAAATCACCTAAGATGAACAAATGAATTAAAAGTATGATTGAAAATTAAATAAAACTATCTGATATCATGTAGAAATAAATTTGGATCGAAAATTGAATTGTGAATGATCTCCAAGGCACGCTGATACGCTATCTTTTAAGAGATGCTTCTTCCTTCCACCAAAGTCACAAGGGATGCAGAAAATTTCTTTCAGGATACAGTGGAGGTTGTTTGATTCAAAAGTGACTTATTTTGATACAAATCAATAAAACATTTTATGAATTTTCGATGATGCTAACATACAATAAAAATTAAAAATAAAGCTAAATGTTTTGTAATGTTCTATTTATAATAGAACTCGTGGCTATTCTGTTAAACTCTTCATGAATTAGTCACGTCCTTTAAAAACAAATATATTTCTTCATGTAAACGATAGGAATTTTAATTTCTCTTTCAAGGGCGTTGCCCCCGAGCCCCCACGAGGGTGCGCTGCCCCCTCGACCCCTGAGACCTGACAGGGCAGGTCAATCCCTGTTTTGAAGCGGTAAACAATAATCTATGCGGCAAAATATTTACGACAACAATAAATTTATGAAAAATTTCTAACATATCAGACTTGAGATCAATTGTTAAAAGTTCATTTAAGCTCGACTCATTAATATAACAAATCAAGTTTGAAAAAAAAAAAAATTAACTCAAGAAGATTTTGAACTCACCTCTAGATCGATTAGCTAGATTAATTAAAAATTATCAACATCTATGTGTGAGATTAAGCTAATTATGCCAAGTTGATTACTAATTAAAACATTATAAAAAGCCAATGTAATTAAAAGCTGGATAAAAACTTAAATAAACCTAAATTACCTGTTCCACTGAATTCAAATGAATTACGGTTCATAATTAAAAGTTAACTAAAGACAACACATGCATTCATACCATAAGAAAAGCAGCATCAGCCTTAGGAACCATTTCAAACATATTTCCACCAACATGCTCAACACCATCACACGACGGGGCGACGGCAACCACATGTGGGAGATCGAAGTTAATCCCATGAATCCATGGACAATACTTCACCAACGTACGAAGAGCTGTCCCATTACCACCACCAACATCAACCAATGAGCTAATCTCCTTAAACACCTCAGGATAACGATTAACGATAGTTGGAATAGCCAGCTTAGCATGGCAAGCCATTGCATCGTTAATTAGCTTACTATGGGCAGGATTTTCAGTAGCATAATCCCATACATCTCCCCCATGTGCAGCCTCAAATGCTGAATCCCCATTGGTTAGTGCTCGTGTCCTCAGATTGTGCCATGGAGCAAGCATCACAGGGCTACTTTCGAGCAAAACAAGAGCAGCCATGGTATTGGCTCCATTTTTCATGAGGAGACGAGAAAGTGGTGTTTGGGTGTAGCAAATTTGGGAGCCTTGGCTTGTAAGCTTGTGCTTGAAGATACCGCGGTGGGTTAAGTACCTCATTATGCGACTGAGGATGGAAGGGGAGCAGCCAAGAGCGGTGGATAGCTCCGGTAGCGTCATGGCTCCGCCGTGGCTTTCTAAGACGTCGGGGATTTGGAGTTCGATGGCGCATTTTACTACTGCCATTGGAACGAAGCCAAATATATATTGCCATATATCTACTTGGGCGTGCACTTCTTCATCTACTTCTTTGAGTGTTTTTATGTCCATCTTTCTTTGTTGTTTTTGATTTAAAAGAAATTAACTCCACCACAATTATATAGCTGCTCAAATAGGTAAGAACCCTTTTAAATTTGAAGGAAATATCTAAGCAATTCAAGATTTACGATTGAAATTATTAAGATATCATAAATCTGGACAAGCCCACAACATGTACCACAAAAATGTATACATTTGAAACCTAGCACGAAGATAAAAAGTTCACAGAACTATATATGAGCAATCTAATCAATTGTTATGTTGTCCTTACTCTGTCTGTGTATATATGTAGTTAATTTTCCACCACCCATATTTGAACGTAAAAAATATTTAAGGAAAACAAAGAATTAAGACGATCCAACATGGTGTAAGCTCATTCAAATTTCAGGCAGAATTAGCTATCTTCTAAATCTTGGAACACGAAGAATGTCTTTTATAAAGACAATTAATTATAATGCGAATTAAGAATTTGGCAATTTGCTAAGTCACACTGTCGAGATTAAAGATTAAAGTTTTATAATACTTAACATTTTGGAGAAAGGGACGAAGAAGAGATTGGCTTATGTGGGGTTTCAGGTGAAGGAGAAGAAAGAGGAAGAAGACGGGAAGAATACTGCGGTGGCCGCCGC

>CbuCOMT22

ATAACAAATAATTTTTTATCACTATTATTTTCTATTAACATCAGTTTTCAACCATAATAATTGCTCAAATTTTTCGTCGTGTAAATATAAAATAAACATATATTAATAAAATATAATTTACACCAAAAAGGTAACAAACTTTATTGCAATTACTAACAAGAGGGAAACAAACTTACAATAGAAGAAATTGCAATTTCTTCTCTTTCTGTTCTATGCATCTTTCCTCAGAGGAGGAAAAGCATAATCACACACTCACAATCAAAACCCACTCAAATTCATTTATAAAGTTCCATAATCCATGAGTTGTAAGCACAGCAAACTTTCTTGAACTGCTTAAAGCCAGCCCCCTTAGCCAGTGCCTGAAATTCCTTTTCTGTCCTTTCCTTACCACCTGGATTATGAGCCAACATGATCACATCAACATGGACAACATTCTGGGTGGCCAGTCCAGTGTCTGGGGCCTCTGGGAGAAGACACTCAGCCAGAATCACTTTCCCATTTTGTGGAAGGGCTTCATAGCAATTTTTCAAGAATTTGAGGCAATGTTCATCACTCCAATCATGGCAAATCCACTGCAAGATTATGATGGAAAATCATTATTAGATCACTAATGACAATGATTATTGGAAAAGAATTATAAGTAGGATTTTTTATTTTTTAAAATCATAAGTTTAAGTTATTTTTTTTACCTTCATGAAAATGGCATCCCCTTTGGGCACACTCGCAAACATGTCTCCACCGACATGTTCCACACCTGCATAATAAATTCTGCATTAATTAGATGGTAATTTGACCAAATCTATTTAAAATATTCTATAAAGTTCATAAAGGGTTTTTGCAAGATTTTTATTTTTATTTTTTTAAAGAAAAGTGGAAAGTTGCAAAAAAACGATAGAAAATATTATAGTGAAAGTTGAAAGTGTAAAGATGAAAGATAAGAGAAATAAATAAATAAATAAACTGCATGAGTTGAGTGAGCCTTGTTTTAACTTTAATTTTTCTATACTTTTCTTAAGAGAATCAAGAAAATAATTATACTACTTTCAAACATTCTAAAACAATAAATATTCAACTAAAAATTGTAAAAACACTTTTCTCAGGGGGTGTTTGATAGGAGAAATTGGATTGGATTGGAATATATTATATACCTTAATGTGNGATAAATATTCAACTAAAAATTGTAAAAACACTTTTCTTATGGGGTGTTTGATAGGAGAGATTGGATTGGATTGGAGTATATTATATACCTTAATGTGTTGTTTGATAGGAGAGATTAAGGTATATTCAATATACCTTAATTGATGGGATGATATTAGAGTATATTGGATTGGGTGGTCAAAATACCATATTACCCCTATCGGAATAACAAAAAAAAAAAGCACACGGCCGTGTATGTTGTGTCACACGGCCGTAATGTATTGCAGTACTTCGAGTTGCTTCGAGTTACACGGCCGTGTGTATATACAAACACGGCCGTGTGGATTTCTTATTCATTAAAATACACGGCCATGTATGTTGTGTCACACGGCCGTGATGTATTGCAATACTTCGAGTTGCTTCGAGTTACACGGCCGTGTGTATATACAAACACGGCCGTGTGGATTTCTTATTCATTAAAATACACGGCCATGTGTATTAATAACACGGCCGTGTATTAATAAATTGTTCTGAATTGAACATTACACGGCCGTGTGCGTATAGATTACACGGTCGTGTCATTCATGAACTGAGCAATATAAAATAAGGGCATTTTGGTCATTCATCCATTTTTGGCACTTAACATTCCATTTGTTTGTGTATCAAACAAGAGATTAAAGAATAATAATAGTTCTAATATACCTTAATGTGGTGTTTGATAGGAGAGATTAAGGAATGTAATATACCTTAATGTGGGGTAGAGATATTAAGGAATATTAGTAATATTCCTTAATCTTTGATATCAAACACCCCCTTAAGGTATTGAATACCCCTATAAGATGTTTTTGGAGCTTATAATATATTAATAATGACAATTTTATAATTAATATTGTTAATGTAATTAAATAAGCATGTCATCAAGTGATTGATTTTACTAACTTGTATGATTTGGATACTTACCCTTTTTGCCCGTAATTATTTTTTATAATGGGGCCTTACTACAAGATTAATTTTAGGGGCATATTAGCAGATTTACTGATATTTTATTTTGAAAACTCATAAACTCTCTTTTCAAAAAAATAATTGAGCACTTTATGACATTTTATAAGCTATCGATCTAAAAATATCTTACAAGATAATTTTAGAGATCAATCTTATAAATTCACCCAAACATCCTCTTAGTTAATACACCTTCATATAGCTTAAAGACAACACTTTTCATTACTTCCAATGAAGGCTTTTAAATTAGGGATAATTCCCTCAACATCCCCAAACATTTGTTATAATTACCAAAACACTCCATAAAATTTGAATAATTATAATTACAATTACACCACTTCAGCTGACGTGTAATTACAATTATACCCCTTGTCTAGGGGCCATACTTAAAGGTGTCATTATAATTATCTATCAACAGAATTGATAGTAGGAAGTATAATGATAATTATCCAAATCTCATAAAGTATTTCAGTAATTATGTTAAACCTGAGGAGGTACTGACGTAATTATCCCTTCAAATTATTAAAGAATGAAGTATAAAACACAGGAAAAAAGAACAAACCTGGATAAGATGGAGCATCTTCAATAACATGAGGCAAGTCAAAGTTAATGCCCTTAATGGAAGGATACTTGGAGACAATCATACTGAGTATGGCTCCTGTTCCTCCACCAACATCCACCACAGTTTTCAGGCCCTCAAAACCATCATATGTTTCAAGAATTTTCTTCATAATTATGGTTGAATGATTAGACATTCCATTGTTAAACACCTTGTTAAATCTTGGGTCTGTCCCATGGTACTCAAAGGCACTCATTCCATAGGCTTTGTTGAAGGGAATTCCACCATCAAGAACTGCATCTTTTAGATGGTACCTAAGATCCAAGAGATGGTTTATGCAACTTAATTTGCGTGGGAAAGAAAAATTATGCACTTTAAATATAAAAGTATTGGCAAATACGGATGGGATCCCCTGTTCCACTTTCCCGTTCCACTTTTATATTCTATCAATAATGCAGCGCTAAGTTTATATTGGAACAGTAGAAATCGAAAACGACGTTTGCAAAAATTCAAACGGAAACTGTTCCACTACACAAGTGACATTGTATTATTAGTGGAATAGGAGATCCTGTTCACGGTTGATTAATCCTTTGTAGTTTATTCAAATGGCAAGATAGATATCTCATGATCATAGAGATCATGAGTTCGATTTTCACTGGTGTGTGAAGTTGTTGTTTTACCTTTTGATAGACGGTTAAAGATTTTTGTATTGTAAACTGATGAGTGTTTTCCTTGTTATTGTATTAATCTTTAACTGTTATTGATGTATTGATGTACTGTCATATTCACTTCAAAACTAAATAAATACAAGTATCCATTTTTTAAAAACTCCTACGTTTGAATCGTTTACAAAAAGGCTCCGTCTTTTAAAGTTATAGAACATCCCATTAAAGTTTATTTTCATTTTACAGATTTCATTAGTTAGTAGTTGCTATTACATAAACTTTTAACAAGTACACATCACAGGATGTTAGCCTAAACTCATTTAAAACATTTTATAAGCTCATATATTTAATAAGCTATTATACCTTATTCTAAGGGTAAGTTATTAATAAATTTAGATAAAATAAAATTTTAAAACTTATAATATATTAGTAATAATAATTTTATGATTAAAATGATTAAAAATTAAAGAGGCATATTAAGAACTCCCTTAAAATAAAATAAAAGTCCTTCAACTTACTTCAATTTGTACATATGACTTATATGGAAACTTTCTAGTGATCAACCTTATAAATTCAAAAAACGTGGTACCCACCACTAGAAATTGAACCAATTGATAAAGATAGCTTTTGTCTGAATATTTTGGCAACCACACACATAAAAGAATATTTTGTGCGTGGTAGGTGAAATAATATGTGAACCCTACAACTAAATGATCTATTTTCTATTAATTCAATTACTTAATTATAGCTTACTTGGTTAAAAAACTAAAGTAGACTAGCTACCAAACCTAAATTATTAGACTTGAACTAGAATTTTAGAGATTCGTTAAGCTATCTGTCCAAATGATAAAAGGTGGTTTAACAGATCAACAATCTAAATTCGAATCTCTCATATAAAACACCCCCACTCTTATTTTTCCATCGTTATAATCTAAAAAATTAGAATTTTCCAAGCATGATGAAGTGAGAATCGATAAGGCTTAGGACATTTATTAGGTAACTAAAATTAGAAAAAGAGAAAATGATTTTGTTGACTTACTTGGTACTCTATTTAATGTATCCCAACTTTTCAAAGTTGATCAAATAAATTAGGTGCGATAGGCGTTCTTTAATGTAAGGATTTACACTTTAACCTTGTTTGAAGTTAACATAATTATCAAAATATTAGAATATTTTCTAAAGTCGAAAATTATTGCACCTCCTATCTTAGTTCAATCAATGAAAAATTTAACGAAAATTGTGTAATATTACAATTATACCCCTTAACGGAAATTGTGAAACATTACAATTATACCCCTTGTTAGATGTCATAGTCAATGTTGTAGTATGGGGTGTTGGAAAAATCTTAGATTATTTTTAGTTAATAATCATAAATTGCATTTGATTATTTTGTCGTTACAAAATCTTAGAGTTAGGGTAAAGCAAAAGTTTACTTATATTTTCTTAATGATATCAGCATTTTCCATACAAAATTTTAACGAAGTTAAAAAAAATAAAATACAAAACTAGGAATAGGCAATTTGGTAAACATTATATTTTTGAATGAAAGATATTGATAATTTTAAAAAATAAAAATAAAAAAATTGAGGTGTAATTAACCCTAATTGTTTTTCCTCACATAAGTACAATATAATATATTTTTTCATTTTTTTTATTTTGGTTTTTTGACGGAACATTCTCATTTTACTATTTACTACTTTTCCATTAATTGTCAAACACTTCCTACTTCTGGAATAAACTATTTTTTCTAAAATTTTTAAAATTACTCACTTTTTTCAAAATAAAGTCCCAACAATAACATACACCTCTAGTAAAAATAAAATAGAAAATACTACAATCGTCCACTTTATTTTTTTTTAAAAGTGGATGATTGTAACAATATAATCAAATATTTTATTTTTATACTACAAAGTTTTCTAAGTCGAACATTAATTAAATTAAATTTCAAAGCCCATAATATAATTAGTTTCAATAATAAAAAAAAGAAATATTAAATTATGTAATTATTATAAGAAAAAATTAAGCTTAATTACCAGCTCTCCATAAGGACTTTATCATGGTTCATGAGCAACAAAGGCGCCATAGAAACTCCATCGTCGTTCTTCGTCAAGAACTTACAAACCGGCGCCAACCCATACCGCCGCTCAACGCTGCCATCCGGCAGCGTTTTCAGGCTGCAGTTCAGAATATCATAGCTCGCGAGAAGGCGGAGGATTCTGTCAAGCATTACACGCGCCTCCTGATTGGTGGTGGGAAGCTGGGCGGCGAGTTCCGCCGGCGAAACAAAGGCACCGGGGCCGGCTTTCTTGATGAGCTCAAGCAAATCGAGCTCAATGGCGGATTTGAGAACCATTGGAAGTACGGAAGCACTGGCTAATTGCAAAGCGAATAGGCAAGATTCTTCATCTGAAGAAGCCATTGTTGGAGCTCCAAGATTCTTGGTTGATGAGCTCATTTTTAAGTTTGATCCTTATTGGGTTTTTTGGTTTGTAAGTGTTGGGAGTGTGGGG

>CbuCOMT23

CTAAAGCCCGCTGCCTTCAGAAGATGTGCCCATTCTTTGTATGTCCTCTCTTTGCCTTCGATCAAGTAGGCCCGTCATTACCATATCCAGCGACAAACGGGCACCTGTGTACTCATCTTCTTCGCCGTCTTCGTTTATGACAACTTCTGCGATGATCACTTTTCCGGTGTCTGCCGGAACAGCTTCTTTGCATTTCTTGAGAATGTCTATGCACGCTTCGTCGCCCCAATTATGCAATGTCCACTGTCATAAAATAGTATGAGAAAACGCTTTTATTAATAATTAATGGAGAATTTGTGATGGAAAGGGTTTCTTCGTAAAAAGATTATATTTGTGATGGAATTTTGAGCAAATTCAGATATATAACTTATTAAAATATATTATGTACAATATCATAATATATATAAAAAAAATTAAAGTATTATTTAATATATGTAAATATATGAATTTGTTCATAGAAGATGTTGTATGTATAGTAATATTTTAAATATTATTTATTAGATATGAACAATGGATATAATTGAATATGTAAGCGGATATATCCTTTACAAAATATATTCAGAGCGATTTAAATATGCGGTTACAATATTCGTACCCGAATACTCCTAATTGAATACAGATAATTTTCACTTTAAATAAATATGAACAATACTTCGTATATACAAGTACGAACATGAATAGTAATAGAATTTTCAAGTATCCATTTACAGTAACAATTGTCCCCAACACATATCAATAGACATTCATAAACACACAACACAAGTTAGGTCAATCAAACGACTCTAAATCATTAAAGTAAACATACCAATAAATCTAAAGTAACCACTCTAAACCATCATCATTAACGCGTACCATGAGCATAACTGCATCTGCTTTTGGGAAAGTATCAAACATACTCCCTCCCACGAACTGAATCCCATCAACAGAAGGAGCCTTAGCCACAACCTCAGGAAGATCAAAAGCAATCCCCTTAATCCATGGAAATCCCTTTATCAACATACTAAGGGCTATCCCATGGCGGCCCCCAACATCCACCAACGTCCTAATTCCTCGGAAAGCTTCCGGACAGTTTTCAATAATCGCCGATGTTGCCACCCTAGCATGGCACGCCATACCGTCCGTAAACACCTTTGTCGCATAGAGTGGATTGTCCCAATTGCTCTCGCCGTTGAGAGTCTTAAAATCGGGGCAATTTCTGATTTTCAAAGCATCCACAGTCAAGCCCTCATAGTTTGAATGGGGACCGGCCCCCTGCAGCAGTATGAAGGGTCCGACGTTGTCCATTGTTAAAAGAAGAGAAAGTGGCGTGTGAGCATAGTATACGACGTCGTCTAAGACTTTGCTCCGTGGGGGCTCAATTTTCTTAAACATGCCATGATGGATTAAGAATCTCATTATGCGGTAGAGTTTATCGTCGGGGACTCCCACGGCGGCGGAGAGGTCGGAGAGTGAGATAGCACCGCCGTGTTTTTTCATTATATCGGGTATTTCAAGCTGGACGACGACTATTATTACTCTGGAGGTGACATTTCCGAAAGCATATTTCCAGACATCTGCACGAGCTTGTGCTTCTTCATCTAGCACCAT

>CbuCOMT3

TATATACACATATATGGATAATTCACCAGAGAACCATCTTAAACTAACATAGAAAAACCATGGAAAACGCCATTAACAGTAACACCAACTCTGCAAATCTTGAAGAAGACGAAGCATTTGTCCAGGCTGTAGCTGCTATTGTATCTTTTGCTCTCCCTGTGGCTTTGAACACTGCCATGGAACTCGATCTCTTTAATATCATCAGAAAAGCCGGCGAGGGGGCTGCCGTTTTGCCTTCTGATATCGCCGCCCGCCTAATTCCAATTTCCAGCCTGCCGGAAGCAGCTGCCGGCGGGATCGATTGCTTGCTTCGACTGCTTGCGAGTCACTCTCTACTCACTTGCTGCACAAGTGAACTTGCTAATGGCGCCACTGAAACCCGATACGGCCTTGCGCCGGCGGGGAAGTTCTTTGTTCGAGACGGGAATGGAGCTTCGTTTGCTGCTCACCATGAATTCTTGCGTTGTCAAGCTGGATTAGTGGAGGGCTGGTATGTGCTATTTTGCTACTTGTATACAATATGCTTGATTTCGTCTTCTTATGATTCAGTTCGGATCTTCTATTTTAAAATTAGTTCATCTTTATTTTAATATTCTTGATAATATATTTGACGTGAATCGTTATGATAGAATAGGAGACGAAGTATAATTTAAAATAGAAAATTTAAAATAATTTTGATTAGTGGGATAATGATGACAGATTCATTTTTTTGCAATAATTAGAATGCAAATTGAACGTATGTTGAAAAGACTCTTATGGTCATGTTCGAAGAGTCGTTATTTGTTACCTTGATTGTGACTCCGCTATCTTTAATTTATATGATAATAGAAGTAAAATCTCAGTTAACCCAACTTCATTAGGTGAAAACTCTTTACCTGATCAGGTGCTTCAAATCATTTGGATCCTATTCCAATTCATATCGTACGGGTAGTGATGGATTTACACTTGATCAAGTGGGATCAACTTATTGACCGCACTTAACCCTACAAAATTTTGTCCTTTTATGTATAATAGTATAATTTGAATTTTATTTTTATTAAACGTTTATAAATTTAAATTTAATTTTTACAAGCTAGCGATAATCCTAAATAAGCTACTTATGCCAAATGTATTACATTTACAACTCTGTGTATATCTGTGATTTTAATTCTAAGAAAATACTTAATATAATTAACATCTGATCAGTAAAATATGAAATGAAATATGTATTGCAATAGAAGATCCTAACCGGCTCTCAATTGGTTAATTTCAATAGTACCCCAAAAAAAAAAAAGAATTATATTTTTAGAACGAAAGTGTCGATTAATTACATGCTTTGATTGTTTTTTCTTTTTCTTTTTTGTAATGCAGTAACAAGTTGAAAGATGCAGTTCTTGGAGGTGGGAATCCATTTGAAAGGGCTTACGGCACGTCTATATATGAGTACATGAAATCAAAACCCGATTATAGTAGAACATTTCACGATTTCATGACAAGTTTTAGTGTTATGATCATGAAACGTGTTTGTGAAAAATACAATGGTTTTGAGGGGTTAAGTTCAATCGTAAACGTTGGGGGTGGTAGTGGTGCCACCCTTGATGTCATTATTTCAAGGTATCCTTCCATTCATGGGATCAACTTTGATTTGCCAGAGGTAATACAATCTGCCCCATCTTATAAGGGTATGTTTCTCTTTCTCTGTTTTTTCATCAACAAGGTCAAATTAGTCATTTCAACATATGTTATATTGAACAGTAAAATTGTGTTGTACATTTTTGTTATGTCTATGAATTGTTTTAAGACTTTGTTACGTTAGGTTGAGTTAAATTAGATATCACTTACAAGCTAAAGTTATTGATGAATCGACCTGGCTTCTTTTTCGATTTCATTGGTGTATATATGTGTGTAGGAGCTTGTGCTTCTTGAAAGTGACTAGTCGTGGAATATAGTGAAAATGAAAACTTTTTGAAAAGATGAAGTGATAATATTTATAAAAGTAACTGCTATATTATTATTAATAAAAAAAAAAAAACTTGTATATTAGAACTGAGTTTTTAACTACTTTCTTTTTAGGTTTTAAAAATAATTATGACAAGATTATCAAATATATCATATAAATAATGAGTAATTGAAAAATTAGAAGTTGGAAAAAACACCTTAATTAGGGTTGTTACAATTCCTACCCTATACTCCGCTTTTTAAGAGGTCATGTTAAAATTGTGTTGTACATTTTTGTTATGTCTATGAGTTGTTTTAAGACTTTGTTACGTTAGGTTGAGTTAAATTAGATATCACTTACAAGCTAAAGTTATTGATGAATCGACCTGGCTTCTTTTTCGATTTCATTGGTGTATATATGTGTGTAGGAGCTTGTGCTTCTTGAAAGTGACTAGTCGTGGAATATAGTGAAAATGAAAACTTTTTGAAAAGATGAAGTGATAATATTTATAAAAGTAACTGCTATATTATTATTAATAAAAAAAAAAAAACTTGTATATTAGAACTGAGTTTTTAACTACTTTCTTTTTAGGTTTTAAAAATAATTATGACAAGATTATCAAATATATCATATAAATATTGAGTAATTGAAAAATTAGAAGTTGGAAAAAACACCTTAATTAGGGTTGTTACAATTCCTACCCTATACTCCGCTTTTTAAGAGGTCATGTATAGAATTAGGTAGGGTTTACAATAATTTAAAAAAAATTATTTATTAATTTTTAGCAGAATTTTAGCTATTTTATATTGTGTGGTAGCGCTATTCTCTAAAGTTAAATAGAAAAACTAAATAAATAAATAAATAAATAAAATCTGAAAGCAACCACCACCCATGACCCAACTTCTGCAGCTTTTGGTAATTTTGCGCTTTCAAATACTTTTAATTTTTACTATAACTTTTTACTTTTTCAAAAAAATAAAAGCTCATACAGATGCTTTAAATAGAAGCAAATAGCAAAAACACGCCTTACGTGCTTTTTTTTCTTAAGTGAAAAGTGCAAAAAGCTCCACTTCTTGCAAACAATCCTATGATATTGAATGTGACTTCAAAACCTATAATGATTTGCCTAAGTTTTAAATGCAGGAGTACATCATATTAGTGGAGATATGTTTGTCCAAGTGCCACAAGGAGATGCCATCTTGATGAAGGTACATTTTTTCTTCCCTTAAAAATTCAAATTACACCTCACACAAAATATAATATATAGTTGATCTATATTAATTTGATAAAAGTGAAAAATATTCAACGTGACAACGTAGTTTATATTGCACAACTGGAATGATGATCGATGTGTACAAGTCCTGAAAAACTGCTACGAGGCATTGCCAAACATGGGGAAAGTGATTATAGTGGACTATATTCTTCCAGACATTCCTCAAGATGATATTCATTCAAAAATGGTTTCACATGTCGATTATACGATGTTAATGTTGTGTGGATCAAGGGAAAGGACAAAGGATGAATTTGAGGTATTGGCTAGGAAGTCAGGGTTTTCTGAATTTAAGGTTGTTTGTAATGCCCATTGTGTTTGGGTGATGGAATTTATTAAATATGGATAAGAGTATTTTGGGGAAAATGATGGTATTGCTACATAATTTACACTTCATGTAATAATAAGGACCCTTTAGTTGGGTTTTCATTTTAAATTTCAGTTTTTATTAATTCTAATATACGATAAAGTAGCCGTTGTCCATAGGGGACTGTACGGTTCAATTTCCACTA

>CbuCOMT1

ATGGCATTGGCCGATGGAGAGCTATCCACAGAGCAACTTCTTGAAGCTCAAGCTCATGTATGGAACCACATATTCAACTTCATAAACTCCATGTCTTTAAAATGTGCAATTGAACTAGGCATACCAAACATCATCCACAAACACGGAAAACCAGTCACACTTTCTGAATTAGTCAATGCCCTCCCCATTTGCAAATCAAAATCTCAATATATCTATCGTTTAATGCGGGTCTTACTCAACTCCAACTTCTTCATCAAAGTCAACATATCTAACGAAGATGAAGATGAAGAGCGTTATTGGCTGACGCCATCCTCTCATCTCCTCTTGAAGGACGCATCCTTGACTGTGGCACCCTTCGTGCTACTCGTACTGGATCCAGTTTTGACAAAGCCATGGCACTATCTGAGCGAATGGCTTGCAGACGATCACCACCTCTCACCCTTCAAGATGACACACGGAATGATGTTTTGGGAGTACGCACAGCACGAGCCACGGCTAAATAACTTGTTCAATGAAGCTATGTGTAGCGACACGAGGCTAGTGACTCGTGTACTCAAAAACTACAAAACTAAACAAGTGTTTGAAGGGATCAAGTCATTAGTGGATGTAGGTGGTGGCATTGGGACAATGGCTAAGGCTATTGTGGATGCATTCCCGGGCATGAAATGTATTGTCCTCGATCTCCCACATGTTGTCGCTGGCTTGCAAGGGACTAATAACTTGACCTATGTTGAGGGAGACATGTTTCAAACTATTCCTCCTGCTGATGCTGTTTTCCTCAAGGTATGACTCTCCATCATTTTTCTCTAATTTCCTTACAAAATGATTCTAAATCATCTATTACGTTACATATTTTACTTTCTATTTCATTACATAGATACATTTCAATAAGTATATTGCATCTACAACTTTGTGAGTAATTTATCAGAAAACAGTCAATATATTTGGCTATGTATCATGCGATAGAATAGCAACATATATTGAAAGATAATCTTAGTTGAACTGTTGCAAAATATGAGCTATGTCCCATTAATCGTTTTTCAGTATAATATGTAGTTTTGAGTAAGTAATAGAAGAAAAGGGAAAAAGATTATTGCTCTTCAACATTGATTTCTTATGTCATTAACACTACAAGAAAGAATCTTCTTTACTAACGGAAAAATCATCACGTCTACAATAAAATTTGTCATTAACATGATTTTGTGTGGATTTTGTTAATTATGTCATATATCCGTAAGTATATATATACCTTGTCACTGATTAATTCTACGATGATATTAAAGTCCGTCACAATATTAACTTTTGTGATAGAATTTTTATCACAGACCAAAAAATTATTATAAAAATTTATCGAAAATTCATTAACGAATGTCATAATTCATATATATTTTGCTCATTTTTTTTAAAATTCATTAATATTCTATCATAAATTTATCACATTTATAATTTATTGATAAGGACTCCTATCCATCACAAGCTTTATTTTAGTGAATGTTTCTTATACCAATTTTCATTGTTTTAACTGATGAATTGCAAAAATTACCAATATGTTATTCATGATTAGCACATACTCTTATATTAAAAAAAAAATCAACTAATTGTACCTATGTGTTTTCAATTTTCTTGCATAAACTACCCTTGTAGAAGGGTATCAAATGCAATAAAATTGAAAATACGATAGTACAATCAACTGATTTATTTAATATAGGGCAATATGTGTCAATATGAACACAGAGGTGTTTTCTGTAATTTACCCGTATTTCAACTTCAAGAACAGCAATTAAAAACAATACTCATGCCATATACAAAATAAACAGTGGATATTGCATGATTGGGACGACGAACACTGTGTCAAAATATTGAAGAAATGCAAAGAAGCTATACCTGCAGGAAAGGGAGGAAAGGTGATAATAATCGATATGGTTGTGGGCATTTATGAAGGAGGGGCTGAGGCAATGGAAGATCAACTATTCTTTGATATGTTGATGATGACTCTTCTAAATGGAAAAGAAAGAAGTGAGAAAGAATGGGCTGAGCTATCCTTAGACGCTGGCTTCACTGGCTACAAGATTACTCCTGTATTTGGTGTGAGGTCTCTCATTGAGCTTTATCCATGA

>CbuCOMT2

ACCATTAGGGTGGGCAATAAGCTAGTATAACATTCCATTTCCTACAAAATGGCCTTGCTGAATAGAGTAGAGTACTGCACGAAAGATCTTTTCGATGCTCAGGGTCACGTTTGGAACCACATTTTTAACTTCATAAATTCCATGTCTCTAAAATGTGCACTTCAATTATGCATACCCATGAAACTTTCTCAATTAGTCAATGCCCTCCCAATCAACAAAGCAAAATCCAACATTGTCTTTTGTCTAATGCGCGTGTTAATTCATTCCAAGTTCTTCACCAAGATCAAGATCTCTGATGATGATAACCAGAACGAGGGCTATTGGCACACACCGGCTTCACTTTTCCTGTTGAGAGACGACCCCATAAGCATCGCGCCTCTTGCCCTTGCCATGCTCGACCCGGCAATGATAGATCCATGGCATCATGTGAGTGAATGGTTTCAGAATGAGTCTTCCTCATCATTCGTCACCAAACATGGGATGAGTTTTCGGGAATACGGTAAGATTGAAGAAAAGATGAATCGATTATTTAATGAGGCGATGGCTGGGGATGAACGGTTTTTCACTAGTGTAGCCATTAATGAATGTAAACAAGTGTTTGAGGTGTTGAAATCAATGGTGGATGTTGGAGGTGGCACTGGAATAGTGGCCAAGGCTATTGCTGATGCCTTAATTTCCTGGCTTGAAATGTACCGTTCTCGATCTTCCACATGTTGTTGATGGCTTGGAAGGGGCTGAGAACTTGACCTATGTCGGTGGTGATATGTTCCAGTTTATTCCTCCAGCTGATGCAGTTTTCCTAAAGGTTTGTGAAATTTAATCGAGAGGTTGACTTTTTTAGCCATTTAGTTTGACTTAAAATCAAAAATAGCCACATCTTCACAAAACATAAAAAATAGCCACTTTGTCCATTGAAAAGACTAAAGTGCCCTTATGAGTCCACTGAAAAATATTTTACTTTTCATTTCAAGCTCCTGTAATCGAGAGAAAGAGGAAGAGAAATACAATCGAGAAAGAGAGGAAGAGTAATAGTTTCGAAAAATCGAGACAAGAGGAAGAGGAATAGTTCGAGAAAGAGAGAAAGAGAGAAAGAGGGCGAGGACGACACTGATGGACGACGGGTGAGGATCGACGGCGAGGACGACGGCGACGGCAGCGGCGGATGAGGTAGGTCTCGTCTTTCATTCGGTTTCTCGACTGAAAATTTCGATTGAATGCGTATATTTACAGATCTGGAAATGGTTTTGTTGAATTAGTTTAATTCGAATGATTATGAAATGGATTTTGTTTGGGGTTTGGAGTGATTAGTTTAATTCGATTGGGTTGATTATGAATTATATGAATTTTTTGTTTTTGTTTTTGAATTGGTTGAATGTATTGGTGAAGAAGATGATGATCTGGGTTGTTGTCTCGAGTGATCGAGAAACGTATGTTTCTCGATCGAGGAACGTTTCTCGACCGAGGAACGTTCCTCATTCGAGAAACGTTTCTCGATCATTTTATTTATTTATTTATTTTTTTTT

>CbuCOMT4

GAAAAATTGACTAGGCAATATAATGAATCAGGCCATGAAATTACAGTAATAAAATAATTTATTCTCAATCACTCACTTGAAAGCACTCTCCAAACCGTACTATATAAGACACTGATCTTGAATAGTCATTACCAAACAAGGTTTTTTATTTTAAAAAAAGACAATCATCAAACAAGAAAAAATAGATTACATTAGGACAAACTCACTCCATTAAGGATAGGCCTCGATGATTGATTCGATAGCTCTCATATGCTTAATGGTGTATTTGCTAAAGCCACTTGCATTGAGAAGTCTTATCCATTCTTTAGTAGTCCTTTCCTTTCCATTGATAGTTGCGATCATGATCATCATGTCCACCAGCAAACGTGCACCCATGTACTCATCTTCTTCTCCTTCCTCATCAATCACAACTTCAACAATGATCACTTTCCCTGTCTTTGTCCGTATAGCTTCTTTACATTTCTTGAGGATTTCTATACACATTTCATCGCTCCAATCGTGCAATACTGACTATCATACAAAATAAACAAATTTATATATGATATTCTTGTATTAATACTGTAATTTTAGATCGAATAGTTGTACATATACAGGTAACTAGCAATGGATAACTCACAATTACTAATAGCACTGTACACTCGTAATAATACATGTACATAAATTATTTGTACTCATATTACTAAATGTACACACGTTGAATGACGTTCAAGCAATGAATGCTTGTAACTACATCAATCTTTGAAGACTTTTATATGAAGTGGTTTTTTATGCTCTTAATTAATAAAGAACTTGGGTGATTTTCATATATACAATCCTCCTTACAAGTTAAACGGGTTAGAATCTTCTATTCCAAAACAGTGTTTTTCTTCTATGGCATTGTACGAATTCATGCCAAATGTCTTAAATATTTCCTTATAAATACACATAATTAATGTGGATGTCATACTTTTGACATGAATTCATTCGGTGGAATAAGAAATAAACATAAAATGGAATAGGCCTCAAGACTAGCTAATATAGTTGTAACTTATGCAAAATGTAACTATTATTAGATGTTTCACATGCAACCCACTATTGGTCTATCACCACTTTGCAGTCAACATTAACGATACGAGCATTTTCCGGATGGAATCCCCTATTTTAAAACGGTGTTACATCTCTTATTCTATATATATTTTAAATACATGTCACGTGTATTATATATTTTCTTATAAAAATATTTAGTAATAAATGCAATACGCATGACATGTATTTAAATTTTAAAGACATGAAACAGGAACTGAAACACCATTTTAAAATAAAGAATTTCATCCGCATTCATGTAATGTACTATAATTCTAAAAAGGGCAGACACATCGAATATGCAACGAGGGTACCATGAGCATAATTGCATCAGCTTTTGGAATGCTTTCAAACATGTCTCCACCAACATGCAGAACCCCGTCAATGGCTGGAGCCTCATGTACCACTTGAGGAAGATCAAAATTAATCCCTCGAATCCAAGGAAAAGCCTTCACAAACATACCGATGGCCGTCCCGTCGTTACCACCAACATCCACCAAAGACTCAATCCCTTCAAAAACCTCAGGACAGTTATTGATAACTTTCGACGCCCCCAACTTACCATGGCTCGCCATATGATCCCTAAACAGCTTTTCATAAGCTTCGTCTACTACTCCTGAGCTCCACATCGTCATCTCATCAGCCGCAGGCTTCAGACCAGAACCTTTTCCAGCTCTCAAATCTTCTGCAGTTAATCCAAACTGTTTTCCCGACGGACCGGCCTGTACGAGCACAAACAGGCCCATATTATCTCTTGTGAGAAGACGAGAAAGTGGAGTTTGAGAATAGTGAAACAATGGAGGATCTTGGCTAATTATTTTCTTTTTAAAAATGCCATTGTGAGTTAAGAAACGCATGATGCGGTAGAGAGAGTTTTCAGGGCAACCCACAGCCGAGGATAGCTCAGAAAGTGTCATGGGGCTACCACGACTTCCGAAGACATCGGGTATTCCTAGCTCTATGGCGCATTTCACCACTCTCATGGCGTCAAAACCGTAAGCATATTTCCATACATCTATTTGAGCTCGTGCTTCTTCGTCCATGGGGTTGTGAATTAAGAATATCAAGAAAAAAAGGCTGTGGTAAACTACCAGGGAGCAACTACAAAATGCAAGTGGGTAAGAGAAGACGTTG

>CbuCOMT5

ATGGACGTAACTAAATCACTAAAAGAAGTAGATGAAGAAGTGCAGGCACAAGTAGATATATGGCAATATATATTTGGCTTAGCTCCAATGGCAGTAGTAAAATGCGCCGTTGAACTCCAAATCCCTGATGTCTTAGAAAGTCATGGCGGAGCCATGACATTGCCGGAGCTATCCGCCGCTCTTGGTTGCACCCCTTCTGTGCTCAGCCGCATAATGAGGTACCTAATCCACCGTGGAATCTTTAAGCAGAAGACTACAAGCCAAGAATCACAAATTTGCTACATCCAAACGTCCCTTTCACGTCTCCTCATGAAAAATAGCATGGGTGCTTTTCTTCTAATGGAAAGCAACCCTGTGATGCTTGCTCCATGGCACAATCTCAGAGCATGCGCATTAGCCAAAGGGGCTTCAGCGTTTAAGGCTGCAAATGGAGCAGATTTATGGGATTATGGATCCGAAAATCCTGGCCATAGCAAGCTATTCAATGATGCAATGGCTTGCCATGCAAAGTTGGCTATTTCAAATATCGTTAATCGTTATCCTGAGGCGTTTAAGGGCATAAGATCTTTGGTGGATGTTGGTGGTGGTAATGGGACAGCTCTGCGTACATTGGTGAAGTCTTGTCCATGGATTCGTGGGATTAACTTCGACCTCCCACATGTTGTTTCCATCGCTCCACCGTGTGATGGTATTGAGCATGTTGGTGGGGACATGTTTGAGATGGTGCCCAAGGCTGATGCTGCTTTTCTCATGGTATGTGCGTGTTTTCTCCGGTTAATTTTAATTCATATGATCATCCTCATGTGTGATTTAGATTTGAAATTGACCATTTGATTTGGAGATTGGATCGAGTAGATCCCCTTTGATCACGTTGGTCTATATATTGGATATATCTAACTAGGTGATCACCTAGAACTTGAAATAAAAAAAAAAATAATTAGAATCACTGTGTTTGCTTAGCCGGAAAACCCTTTGAGCTCATGTAAGTGATAATACATCCTGGGATAGCTAAGAGAAATAGTAGATATTCTACAATTGTGTACCTAATTATGTTTATTTTATGTATTTAATTTAGCCTTATCATGATTAATATATAGCATCTATCATCAAATACTTCTACAAGAAAAAATTTACTATGTTTGTAATGTATCCAAGAAACTAATGAAATATGAGATGAAGTACATTTTGAAATAGAACATCCAAATACATTATAATGAGAGACGGGTCAAATTGATTTGGCTCGATTTTTCAGTCCAATTTAACGAATCTTGTCTCGTAATTAGATTTTAGACCCAACTTTAAACGGACACTTTGGGTATACTCTCACTAATATATGCTCAATTTATAATACAGTGGGTACTGCATGATTGGAGTGATGATGAGTGCATCCAAATATTGAGAAATTGCCGAGAAGCCATTCCCAAGGACACAGGAAAAGTGATCATTGCAGAGGCAATGATCGAAGAAAGAGAAGAGGATAAGGTCACCGATGCTCGTTTGGCATTGGATATGGTGATTTTGGTTCACACAGAGAAAGGAAAAGAGAGGACTATTAAAGAATGGGAATATGTGGTTTATGCAGCTGGCTTCACTAAGTGTACTATAAAACATATTGAAGGTGAAATAATATCTGTTATCGAGGCCTATTGATAAGTATGAAACTTTATTTAAATGCCAATTGTGTGGCTTTTAGAATTCTCATAAAAAATGTCTCCTACAATAAGCAATCATATTTCCTTGTGTTGTATGTTGAATGTTATTTATTTATCCTTGTAAAAGTTGTGAGTACTATGATATTGCTCGTTTTTCTTTTCATCTTTCATTATGACGTGGACTTATATAGGCGGTGTATGACAATATATTAAAAGTATTATCAAAAAGTACCCAAAAAAATAAAAAAATTCACCATTGCATATAAAAAATATATAATTATCTTTTCCAAAATATCTTTTATAAATATAATTAAAATATAAAAATAATTTAAAATTACAATTTCACCCCACTTGTTTTTTTTGTTGCTAAAAACTAGTTCGAAGAGCCCCAAAGTATAAATTAAAATCTCTCTTGCTCCCACTTGCAGTCTATGGGATTCAGATCCTTTCCAAACCTTCCCTTATCATCCCCTTATTAATGCAGTGTGCGTTTGCTTTGATTTTATGCAGCTCTACTATAAATTAGGCAGCTCTACTGTCGTTTTAGGCAGCTCTAAAGTAGTTTATATATATATATAATGCACACAATTCTACTAGCTGTATGGTTAAAGTCAATAAAAAACACTTCCTTAAGTAGCGAAAACGATGTGCATCACCTTTTTCAATGGTCATTTCCACATTATTTAATTTAGATTAGACTATGAAATTTGGCGAAGGCAGGTGTGCACTTTTATGGATATTTCCTTTTGGTCAAATTCGAAAGCCCTACTCTCATTTTATTTTGAAACCAGGGGAGAGCAACTATACAAAAGACATTGGGAGAGCAACTATACAAAAGACATTCTTCGTGTTCCAAGCTTTAGAAGATAGCTAATTTTGCCTGAGATTTGAATGTGCTTACACCATGTTGGATCGTCTTAATTCTTTGTTTTCCTTAAGTATTTTTTATGTTCAAATATGAGTGGTGGAAAATTAACTTCATACACAGACAGAGTGAGGACATCATAACAATTGATTAGATTGCTCATGGTTCTGTGAACTTTGTATCTTCGTGCTAGGTTTTAAATGTATACATTTTTGTGGTACTTGTTGTGGGCTTGTCCAGATTTATGATTTCTTAATTTCAATCGTAAATCTCGAATTGCTTAGATATTTCCTTCAAATTTAAAAGGGTACTTGTTTGAGCAGCTATATATATAGTTGGGGTGGAGTTAATTTCTTTTACAGCAAAAACAACAAAGAAAGATGGACATAAAAACACTCAAAGAAGTAGATGAAGTACAGGCACAGGTAGATATATGGCAGTATATATTTGCTTTCGTTCCACTGGCAGTAGTAAAATGCGCCATTAAACTCCAAATCCCCGATGTCTTAGAAAGCCATGGCGGAGCCATGACACTGCCGAAGCTATCTGCTGCCACTCTTGGCCGCTCCTCTTCTGTGCTCAGCCGCATAATGAGGTACCTAATCCACTGCGGCATCTTTAAGCGGAAGCCCACAAGCCAACAATCACAAATTTGCTACATCCAAACGCCCCTTTCTCGTCTCCTCATGAAAAATAGCAAGGCTGCTTTTCTTCTAATGGAAAGCAACCCTGTGATGCTTGGTCCATGGCACAATCTGAAAACACGCGCATTAACCAATGGGGCTTCAGCGTTTAAGGCTGAAAATGGGGCGGATTTATGGGATTATGGATCCTAAAATGCTGGCCATAGCAAGCCATTCAATGATGCAATGGCTTGCCATGCTAAACTGGCTATTTCAAGGATCTTTAATTGTTATCCTGAGATGCATGTTTAAGGGGATAGGATCTTTGGTAGATGTTGGTGGTGGTAATGGGACGGTTCTTCATACGTTGGTGAAGTCTTGTCCTTGGATTCGTGGGATTAACTTTGACCTCCAACATGTTGTTTCTGCCGCCCCATCGTGTGATGGTATTGAGGACATGTTTGAGATGGTGCCTACGGCTGATGCTGTTTTTCTCATGGTATGTGTGCGTTTTCTTGGGTTAATTTTAATTCATATGTTGATCTTGATCATGATTTGGATTCGGGTTGACCCTTTGAGTTGGAAATTGGATCGAGTGGATCCCCTTTCTAGTTGATCCATATATTGGATATATCTAATAATTAGGGGATTGGGTAG

>CbuCOMT6

ATGGAAGTGATCAAACCAATCAAAGAAGTAGAAGAAGAAGGGCAGGCACAAGTAGATATATGGGAGTATATATTTGCTTTCATTCCAATGGCAGTAGTAAAATGCGCCATTGAACTCCAAATCCCCGACGTCTTAGAAAGCCACGGCGGAGCCATGACACTACCGGAGCTATCCGCCGCTCTTGGCTGCTCCCCTTCTATCCTCAGTCGCATAATGAGGTACTTAACCCACCGCGGTATCTTCAAGCAGAAGCTCACAAGCCAAATTTGCTACACCCAAACACCCCTTTCTCGTCTCCTCATGAAAAATGGAGCCAATACCATGGCTGCTCTTGTTTTGCTCGAAAGTAGCCCTGTGATGCTTGCTCCATGGCACAATCTGAGGACACGAGCACTAACCAATGGGGATTCAGCATTTGAGGCTGCACATGGGGGAGATGTATGGGATTTTGCTACTGAAAATCCTGCCCACAGTAAGCTAATTAATGATGCAATGGCTTGCCATGCAAAGCGGGCTATTCCAACTATCGTTAATCGTTATCCTGAGGTGTTTAAGGAGATTAGCTCATTGGTTGATGTTGGTGGTGGTAATGGGACAGCTCTTCGTACGTTGGTGAAGTATTGTCCATGGATTCATGGGATTAACTTCGATCTCCCACATGTGGTTGCCGTCGCCCCGTCGTGTGATGGTGTTGAGCATGTTGGTGGAAATATGTTTGAAATGGTTCCTAAGGCTGATGCTGCTTTTCTTATGGTATGAATGCATGTGTTGTCTTCAGTTAACTTTTAATTATGAACCGTAAATCAATTGAAGTGGAACATGTAATTTAAGTTTATTTAAGTTTTTATCCAGCTTTTAATTAAATTGGCTTTTTATAATGTTTTAATTAGTAATCAACTTGGAATAACTAACTTAATCTTTTTATACATAGATGTTGATAGTTTTTAATTAACCTAGTCGATCTAGAGACGAGTTCAAAATCTTTTTGAGTAAAAAAAAATGTGTTTGAACTTGGTTTGTTATATAAATGAGTTGAGCTTAAGCGAACTTTTAACAATTGATTTCGAGTCTTAGTATTAATGGTTTTATTTAATTTTAAACCATATTTTTAATTCATTTGTTCATTTTATGTGATTTGAGTTTGAAATTGTTGGGTCTTTAATTGGGTAGGCCTCTGGGTTGGATTGATCTATCTACTTGATATAAGTGAACTAAGACTTCAAAAAAAAAAAGAAGAAGAAGAAGAAGAAGAAGAGGAAGAAGAAGAAGAAGAAAAATTACTAGGATCATTGGATATAATTCCGATGTAAAAACACTTTGATACTCAAGCGAACTGAAGAAAAAATATTGAATTTTTTAACATTACTCGTGTGATTATGTCTAGTAAAAGGGGTATTTATAGAGATTATAATATGTCTGAACCAATATGATTGATTATATAATTTGAGTTGTTTGACTTTATCCAGATAGAGAAACAACTGGGATATAAAATAGTCGGAGAATCTAATTTCCTAGATTTTGTCTTGTTGACATCTGACTTAGTTTTAAATGGTCATTTTGGGTATTAATACTAGTCCCGCGTCATTATCACTATGTAACACTATTAATGCCTACTAATTGTGCAGTGGGTGTTACATGATTGGAGTGACAATGAATGTATTCAGATATTGACAAAATGTCGTGAAGCTATTCCCAAGGACACCGGAAAAGTGATCATCGCAGAGGCAATTATTGAAGAGGGAGAAGAGGACAAGTTTATTGATGTTCGTCTGGCTTTGGACATGGTTATGCTGGCTCACACGGAGAAAGGAAAAGAAAGGACTATTAAAGAATGAGAATATGTGGTTTATGCATCTGGTTTCACCAAGTTTACTGTGAAACGTATTGAAGGTGAAATTATATCTGTTATTGAGGCCTATCCATGAGTATTGTGAAACTTGATTTATGGTTTTAATTAGAATCTTGTAATGTATCTTCTACAATAAGCAGTGATGTATGTTGAAT

>CbuCOMT7

ATGATCTTCTTCTTTGGAAGGTATCATCTAAAGGATGCAATTCTTGAGGGAGGAACCCCATTCAATAGAGCACATGGTATGAGTGCATTCGAATACCTGGCTAAAGATCCGAGATTGAATTGGGTTTTTAACCAATCCATGAACGATCCACCATATTTATGAAGACTTGAAGAATACAAAGGATTTGAGGGTCTGAAATCCCTAGTGGATGTTGGTGGTGGAATTGGAACATCACTTAACATGATCATTTCCAAGTATCCATCAATCAAGGGCATTAATTTTGACTTGCCCCATGTTATTCAAGATGCTCCATCTTATTCAGGTTTGTATTTTAGAAAATATTATAACATTATTTCGTAATAGTTCGATCAATATATCAAATTTTTTGGAGTCGTTACACAAAGACCGTTAAACTTCACGTTAACTAAACTAACGAGACGTAATTTATCTTGTAATTAAGTCAAACTGCAATGTGTACATTGAGTTGATTAACAATACAGCTTTCTATCCTTTACATGAAGTCAGAACTCTCGATCTGATTATAATTTAAAGTATTCCAAATTATGTATAAATATAATTTGAAGTTTAAAAGTTGGCACCTTGTGCAGGAGTGGAGCACACTAGTGGCGATATGTTTGTTAGCGTGCCCAAGGCCGATGCCATTTTTATGAAGGTAAATAAATAAACTTGAATTTCATATTTTTTCGAGAACTAATGTTAATATTCAGTATAAAAGGTGGGGGGACATTGAACTTATAATAACCTGACGATTATTATAAGACTTCCTTCTGATTAATTAGGCTAAAGTTGATCAATGGGTTAGTAACCCGTTTGTGTATTGCAGTGGATATGCCACGATTGGGGCGATTCACATTGCGAAAAGCTGTTGAAGAATCGCTATGAATCGCTGCCTGAAAATGGAAAAGTGATTATTGGTGAAGCTATTCGATCTGAGGACCCAAACAGTTCTTTACAGTCAGCTCTGTCTGATGTGATTATGTTAGCTTTTAATCCATATGGAAAACAGCGATCAGAAAGGGAATTTGAGGCCTTAGCTAAAAAAGCTGGATTCAAACACCTTATCAAAGTCTGCAGGGCTTCTCATATTTGGATTATAGAATTTCATAAATGA

>CbuCOMT8

CCCCAACCCACCACCACTTATATATCCCCCTCCTTTTTCACTTGCAACTTAAAGCTTATAATAAGCTCCAACAGCCATCAGATTACAGCTTATAAGTTCATAATCTTCTACTTCAATGGATAATCAGTCCGATGAAGAAGCCTGCTTATTCGCCTTGCAGCTAGCAACCGGTTCAGTGCTTCCAATGGTTCTAAAAACCGCCATAGAACTCGATCTCCTGGAACTTATCAAAAAAGCTGGGCCAGAAGCTTCAGCTTCTGCTTCTGAACTTGTGGCTCAGCTTCCAACAAACAACCCTGATGCAGCCAATATGATAGATAGAATTCTCAGGCTGCTGGCGGCACATTCCGTTCTTGTTTGCAGCCTGAAACCGCTGCCGGACGGCGGCGTTGAGCGGCGCTACTCCCTTGCGCCGGTGTGTAAGTTCTTGACTAGAAATGAGGATGGAGTTTCTGTGGGCCCTACTTGTCTCATGATCCAAGATAAGGTGTTGATGGAACCTTGGTAAGTGAACTATGTAGTAATTAATTTCTTGATTGGTTTTCTCACAAAAAGTTGTGCTTAAATGAGTTCGGAATTGATCCTTCGCCAGAATTTTGAATGGGTTGATTAATATAAGATGACTTGATGAATTCATACAATTGGAAGATTTTACGATCATTGAGAACTCTTTAACACTTAAGTCAGTGATCTAACTACGGAAATTAAATGGTACAATTGCCTTGAAGTTGATTAGTCAAATATTGTGTGGTTTTCCTTATTTAAGCTAACACACTCGATTCCTTTCTATTAACGGATTGTATTTCGAATTATTTGACTTGATTGGTTGGTTTTTAACTAATTTGGCTAAGTTTACGTTCATTTAGGGGTGTTAACGGGCCGAGCTCGAGAAAATTTGAAGGATTGAGGTTGAGATCGAGCTCGAGACCAATTAACCTATTTTTCGCTGCTCAAGCTCGAGCTTGGCTCATAGGCTCGTTTTGTTTATTGAGCTGAAACCCTTGTTGGAGCTCGAATTACTAATAATGAAAATTGTATCCATAGATTGTCGAATTTAAATGTATATACTATATATATAGTATTAACAGTTGGTCAATCAATTCAAATTCTCTGCTCGAACTCACTAACCTCAAATCTAATTCTATTTGAGCCGGTTTGCAACTTGATCCTCGACTCATTAGGCTTGACTCCATTGACACTCCTAAGTTCGTCTGTCTAGCTAGTTTTCTTAACAACTAAGCCCGAGATATTCTTGACCTTATACACATCTCGCACTTAATTTGTGTACTTTATTTCATTGTATTTATGTAAATATGCTTCAAATGATCTCCTTTGTTTGATAGGTATCATCTAAAGGATGCAATTATTGAGGGAGGAATTCCATTCAATAGAGCATATGGTATGAATGCATTCGAATACCCGGCTAAGGATCCGAGATTTAACCGGGTTTTTAACCAAGCCATGTATGAACAATCCACCATATTTATGAAGAAAATTCTTGAAGAATACAAAGGATTTGAGGGTCTGAAATCCCTAGTGGATGTTGGTGGTGGAATTGGAGCATCACTTAAGATGATCATCTCCAAGTATCCATCAATTAAGGGCATTAATTTTGATTTGCCCCATGTTATTCAAAATGCTCCATCTTATCCAGGTACATATATGGGATAATTATTTGAGTTTTACTTAATTATAATAATATTCTCATAGTTTAATAAATTACAATAATACCCCTTATTGTTAATTAGTTTTGTTGATGGAAAACTTTTAATATTTAAGTCTATGTTAGATTTTCGAGGGGTCTAATTGTAATACTATACAAGCTCCGTTAAATTTTAAGTTAACTGAGCGGGTAAAATGCAATTTATCCTCTTGTGATAAGCGAAAGTAGTAATTTCCCCCTTATAAAAAATAAATAGGCAAAGCGCCCCCTCATATTTTTAGAAAAGAAGCTTAGTACCCCTACATAGGGGGCGCTATGCTATTTCCTAAAAGTGAGAGGTAATTACTACTTTCGTTTATCATGTAGGGGTGGTTTAAATTGCATTTTACCCTTAACTAAACTAATGAGAAGGAATTGTTCCATAATTTTTCAAAATATATGTGTATTTTTTGAATTAGATCAAACTTCAAGGTACCGTTTAGGTAATTTTTACTACTTCCTATTCTTTACATGAAGTTAATAGTCTTGATCTAATTATAATTTAAATTTTATTCCAAATTATATATAAATGTTAATTAAATAATTACTATATTTGAAGCTTAAAAGTTGGAACCTTGTCCAGGAGTGGAGCACATTAGTGGTAACATGTTTGTTAGCGTGCCTCAAGCCGATGCCATTTTTATGAAGGTAAATAAAATTGAATTTTCTTATTTTTCCAAGAACTAA

>CbuCOMT9

ATGGTGGGCTCAGCAGTTGAGAGTTGGCTGCAAGTTGACGTCGTTCCATTTTTGGGAATACACCCTCCGACTACTGTTGTAAGCGAATTTATACTCCTATCACAATTTTTAATCTCTTTATACTTAAGATAAGATGAGGCAATATCATATTATTCTACTCTAGAGACCAGCAGTAATTCTTTGCAAAAGCTTACCCATCACTACAAAAAAAATTGTTTTTTTACAATAAAAGTTTTTTTTTTTTTTGCAAAAAATTAAAATTTCATCATAATAAGTTAATGTGATAAAAAGTTCGTCACAATGTCCTCACAATATCCCTCTCTTATTAAATTATTATAAGGAAAATTGAGTTCCTCACATTAATTTGCATTATATGTGACGAATTTCTCGTCACATCAAATTATTGTGAGGAAAAATATTTTTTTTTTGATGAAATTTTTATTTCCTCACAATATTTAGTAAGTATTTGTGAGAAAATTAAAAATCATCAGACAAAAGTTATAATTCATCACATTACATTACTTTTATATGACAAAATTAAATTTGCTCATAAATAGTTAGTCGCAAAAAATATTTTTTTTTGTAGTGCATGCTTAACTAGGATGTGTGTTTACAATAACATTGTGCGAATATAATGTCTTAGAGTTGGCTTCAGTAGATAATTTTACTTTTGAAAGTTATGCAGAGGCTCAGTATTTGTTCATGAATAAAGCATGTCTCAACAAGTTGCTGCAAAACAATCTGACGTACAATATTTCAAACTCAAGCCCCACAGCACAGAACATCTACACAAGCATATTTTATGTCATTTGGTACAAATTTCTTCTACTTCCTCACCCACTGTGCAAGATGGTTTTCCACCATTTTCTTCCAAGTTCTGAGTGGTGCTTAATTCACTACACATTTTTAGAGCAGCCCTGGAGAATTCTGCCATAGACTCGAACACTCTACAAAACCCAGTCTGAAAACCATTTAAGGTGATTCTTTCTGTTTCTTCCATGCATTTACAGTGTTTCTCCTTCTCTAAATCAACCCTTCTTATGAAACTGTCCAGCATATCCTTCCTTTCTGTCAAAAAGACTCGTCCCGATGTTCAATCTCTAGATTTGATAACTCATTAAAAATATTAAAAAAGAATGATATAAATATCTAGAGTGATTATGTTAATTGATGGTTGTACATAAGAGGGTTTTACGGGGGTTTATAGGCTGTTTGTAAGAATGCAGAAGTGGTGCATGGAAGAAGAGTGAGGCATTTCGAGAGAGAGGGAGGGGGGGGGGGGGGNCTGGGAGAGAGAAGAAGAGGGAAAGCAGAACAGATTGTGCATTATGCATTTTTTCTAACTTCAAGGGTCGAAACGGGCCTAANGCTTATTTGGTCTAGGGCCCTTACTTGAAGGGCTGATTTGTACCTTCTGCCCTCAAACCGCTGAGTAGGTAAGTTAGGCCAGAACAGGTAGACATCATCATCATCATCATTATCTATACGTTTTAATAAAGTGTGGGTCTTGAGTGTTTTATCAAAAATGTCATCTTTTAATTTATGTTTTAATTTATTTTAATAATAATGTAAGTTCCACTATATACGAGTGAGCAAAATTATCCATTAATAAAAAAAACTTGCACACGTGGCACGCCAGAAACATTTTGGAGCATGGTCACAAGCTTCCAGCGTTTCACGTGTGCAAAAACATCGCCATCGGCAAAAAAAAAGATCAAAAATGCGAAATTACAACATACGAGACTAAATTTACAAACTTAAAAAATATAGGACCAAAAGTAAAAATCGCAAAACATATAGGATTAAAATTGCAATTCTCCCTTACCTTTTATGATAAGCAAAAGTACGAAGCAATTTTTTTTTTTTTCACATGGGATAAACCGTTATTTTCGCTTATCACAGGAGGTAAATTGCATTTTTCTCATTTGTATAATCATTTGAATAAAAAAAAAATAAAAATTAATAAATAAAATATAAAAATAGTTCATAATTCCAATGGTAAAGGGTAATTATCCTATCCCCCTATGGTGAACCTGCAACCCCATACCCACCCCTACTTATATATACTCCACCTTTTTCACTTGCAAATCAACTCAATCTTCTGGAACTTATCAAAAAAGCTGGGCCAGAAGCTTCAGATTCTGCTTCTGCTTCTGAACTTGCTGCCCAGCTTCCAACAACTAACCCGGATGCAGCCGATATGATAGATAGAATTCTCCGGCTGCTGGCGTCGCATTCCGTTCTCATTTGCAGCCTGAAACAGCTGCCGGACGGCGGCGTTGAGCGGCGCTACTCTCTTGCTCCGGCATGTGTAAGTTCTTGACTAGAAATGAGGATGGAGTGTCTGTGGGCCCTCTTTGTCACTTGATTCAAGATAGGGTCTTGATGGAACCTGGGTACGTAAATCACTTTGGAATAGATGATCTAATTCAATTTTTCATTATTTATGCTTGAGCTTGGCCCATTAGCTCGTTTTGTTTACTGAACCAATTTAAAAAATCACGTTGGAGCTCGAATTATTGACAACTAAAATTGTATCTATAGATTGTCGAATAGATTTCTAAAATTTATTTAACATTTAGAATTTAGAACTTAGATTTTGCATATATACATATAAAATATGTAGTATTAAAAGTTGTTTAACTGATCCGTAAGTTTATGAACAACATACTCTTTGCTCGAACATGATTATAAAGCTCGGACTCATTGAGTTCATCAAACTTAACTCGATTGACACCCCTAACTTTGTTGCCTATGTATTCCAGACCTTATACACATCTTGCACTTAATTCATGTACTTTATTTCATTGTATTCACGTAACTATGCATCAAATAATCTCCTTTCTTTGATAGGTATCATCTAAATGATGCAATTCTTGAGGGAGGAATCTCATTCGATAGAGTATATGGTATGAATGCATTCGAATACTTGGCTAAAGATCCGAGATTCAACCGGGTTTTTAACCGAGCTATGCACGAACCATCCACCATAATTATGACAAAAATTCTTGAAAAATACAAAGGATTCGAGGGCCTGAAATCCCTAGTGGATGTTGGTGGTGGAATTGGATCATCACTTAACATGATCATTTCCGAGTATCCATCAATCAAGGGCATTAATTTTGATTTGCCCCATGTTATTCAAGTTGCTCCTCCTCATCCAGGTACATGTACAGGATAATTACCTCAACAAGCCCTAAGTGACCTTGGCCTAATAACAGAAACACTCATGTATCTTAAAAAATTATAATATATATGTCATATTGTTAATTTAGCTCGTGATACGCTTGTAATGTTTCAATCAATATATTAAAATTTAGGACTAAAATGCAGACACGTCCCTAACTTATAAGGTCAATTGCACTTTGCCTCCTAATTTTTTCAAAAGTGCGATTACCACCCACCATTGCAAAATGTTTTGTTCCAATCCTTATAATTGGAATCTGGCAGAATATTATGGAAATATTTCGTTTAAATCCCCCCATATACCTTTAGCATAAGTACAATGAGGGTGTTTTAGTAATGATATCATATCTTGTAAAATTAAGGATAAATTAATCCTAGTTTTGCTTAATTTAGACTCGATTTAGCACTAATTTTTATCGTGATGAGATATCCATATAGAAACGCTTTCACTGTACTTGCGCCGAAGATGTAGGTAGCTTTGAATGGATTATTTCGCCGGGTTCGGGTTTTAGGAGGTGAAATCGAACGTTTTGCAATGCTTAATTAGGGGGCTTATCGCACGTTTGAAAAAATTAGGGGGCAAATTGCAATTGACCCTGTAGGTTAAAGGGTTGTCTGCAATTTACTCTAAAATTTAAGGGGTGTAATTATAATGTTATACAAGCTTTAGAAGTTGTTTCGTTGTTAATTTTCAAAGCTATTTTTGTAATTATATTAAAATTTCATGGGGGTATTGAGGTAATTATGATCACTACTTTGTAGAAGTTGTTTCGTAGTTAATTTTCAAAGATATTTTTGGTGTGAGAATAAGTGAGTTTTGTGTGAAGGCCTTGGACCCCTTGTATTGAAATTGAGGAATAAATATAAGGTTGGGACTTGGTCCCGTAATTCGTCGTGTACTTTACTTTTATGCTGCGCAAGTTCTCTCAAACTCGCTAGAGCTCAAGTTCTGCTGGGTTGGTTGGTTAGGCTTACTAGTCGTTGGGGCGACTAGGCCGCACGGGAGGCGAGAGATTTGGCTAAAAATTGGCTAAGTTAAAAACCTCCGTTAATTATATTAAAATTTCATGGGGGTATTGAGGTAATTATGATCACTACTTTCTAGAAGTTGTTACGTTGTTAATTTTCAAAGATAGTTTTGTAATTATATTAAAATTTCATGGGGGTATTGAGGTAATTATGATCACTACTTTCTAGAAGTTGTATCGTAGTTAATTTTCAAAGATAGTTTTGTAATTATATTAAAACTTCATGGGGGTATTGAGGTAATTATGATCACCACTTTCTAGAAGTTGTTACGTTGTTAATTTTCAAAGCTATTTTTGTAATTATATTAAAATTTTATGGGGGTATTGAGGTAATTATGATCACTACTTTCTATCCTTCACATGAAGCTAAAACTCTCGAGTTGATTACAATTTAATAAATATTAATTAAATTAGTGTATTGGAAGCTTGTGCAGGAGTGGAGCACAATAGTGGTGACATGTTTATTAGCGTGCCTAAAGCCGATGCCATTTTTATGAAGGTAAATAAATAAAATTGAATTTTCTTATTTTTCATTTTATCTAAGTTTTTTGTACATATAGTTAACTACCCTTAATACTTCATACATCATTCGACAATTATATTAATGGTTTGTTGTAATAAAATATTCTCAACAAATATATATAAAGACAATCGTAGTCATGGTCATTCATATCACATATAATAATATAATCTATGTATTATCACAAATCAACTCATATTTTTTTATATAATATGATAAAAACATCAACTCCTTTTTTTATTCATATTGATTTTCCTGAAATATATTATTTGGGATATTTTGATGTATTTTTAAATTATATTTAATAAAATTCTCTTATTATTATTCTTCAAACTATCAACAATTCACAATTCACTACCATCACTTTATAATTTCATAATTATATATGTGAATATTTATATACCGTCAAATCCGACTCGAGATCTTGTAGGATCTGGGTCCTTTACTAGTCACAATATCACATATATTCCACTTAATTTATTTAAGAAATTTAATATATTATATATAAAAATGAATTTTTTAATCAAAAAATTATTAATTCAAATTCAAATTCAAATCTGTAAAAAATCATATTACTTCATCCATTTCCAAACACCAACATATATAATTGAATTTATTTAAAGCTTTTCAAATTCAGATCCGGATCATTACCTCCAAAACGGGTCAAGAAATCTAACGGGTAACCCGTTTGTCTTTTTTTTGCAGTGGGTTTGCCACGGTTGGAGAGATTCACATTGTGAAAAGCTGTTGAAGAATTGCTACGAAGCGCTGCCCGAAAATGGAAAAGTGATTATTGCTGAAGTTATTGTGCCCGACAACCCAAATACCGGGCAGAGTTCTTCATGGGCAGCCCAAGGTGACATGATTATGTTAGCTTATACTTCAGGTGGAAAGGAGAGGTCAGAAAGGGAATTTGAGGCCTTAGCTGAAAAAGCTGGATTCAAACACCTTATCAAAGTTTGCAGTGCTTATAGTAATTGGTTAATGGAATTTCATAAATGA

>CbuCOMT10

ATGGATAATTATAAGCCCGACGAAGAAGCCTGCTTATTCGCCTTCCAGCTAGTATCTGGTTCGGCGCTTCCAATGGTACTGCAAACCGCCATAGAACTCGATCTTCTGGAACTTATCAAAAAATCAGGGCCAGAAGCTTCAGCTTCTGCTTCTGAACTTGCGGCTCAGCTTCCAACAAGTAACCCTGATGCAGCCCATATGATAGATAGAATTCTCCGGCTGCTGGCGGTGCATTCCGTTCTCATTTGCAGCCTGAAAAAGCTGCCGGACGGCGGCGTTGAGCGGCGCTACTCCCTTGCACCGGTGTGTAAGTTCTTGACTAGAAACGAGGATGGAGTTTCTGTGAGCCCTCTTTGTCTCTTGATTCAAGATAGGGTGTTGGTGGAACCTCGGTAGGTGATATACAAAGGGTGTCGGAAGCCATCGACATCAATTAACCTATTTTTCATTGTTTAAGCTTGAATTTGGGTCATTGGCTCGTTTCGTTTATTGGGCCAATTTAAAAAACCATGTCGGAGCTCAAATTAGGGATGTAAATAAATCGAGTCTGCTTACGAACTTGATGAATCGGCTCAAAGTGTTTGAGCTTGAGCTAAGTGTGTACAGAGTTTCGAGTCGAACTCGAGCCTGATAACTCTTGTTCATAGTTTTACGAGCCGGCTCGTGAACTTTTTTTATAAAAAATATTTTTATATATAAATAATTAAATAAAATTATTTATATAAAATAATTAAATTTTCTAGTTCGAGCTCGAATATATTAGTTGAATATATTAAAACAATCGAGCTTGAATTGAAGCAATGATTCTATTAAAAATTCAAGTCAAACGAGCTGAGCTCAAATATAGATTCAGGCTAAGTCCGAACCTAGTATTTCTAAGCTCACAAATATGATCCTTCAAGAAATGAAAACCTTCCCACAAATTTTCTTTGGTGTTCTCGCATTTTTCTCATCTATTTGCGAGGAAAATCATTTTTTCTCATTCTAAGTATATTTTTTCTTTTATTTTCCCTCATTTTATTTTCCTAGTAACCAAATTTTCCCTCCTATTTTATTTTCTTTTCCATCCTAAAATCCTGGAACCAACCACAATATAAAACTTGCTCAGTTGATTCATAAGTTTATGAACAACATACTATTTGCTCGAACTTGATTATAAAGCTCGAACTCGTGGAGCTCGATCAAGTTCTATTCGAGTCTCGACTCGCCGGGCTTAACTCAATTGACACCCTTAAGTTTGTTGCCTTGATATTCTTGAACTTATACATATCTCCCACTTAATTCATGTACTTTATTTCATTATATTCATGTAAATATGCATACTCAAATGATCTCCTCCTTTGATAGGTATCTTCTAAAGGATGCAATTCTTGAGGGAGGAATCTCTTTCGATAGAGCATATGGTATGAATGCATTCGAATACTTGGCTAAGGATCCAAGATTCAACCGGGTTTTTAACCGAGCCATGCATGAACCATCCACCATAATTATGGCGAAAATTCTTGAAAAATACAAAGGATTTGAGGGTCTGAAATCCCTAGTTGATGTTGGTGGTGGAATTGGAGCATCACTTAACATGATCATTTCCAAGTATCCATCAATCAAGGGCATTAATTTTGACTTGCCCCATGTTATTCAAGATGCTCCACCTTTTCCAGGTACATATATGGGATAATTACCTCAACATGCCTCCATTGAGTTCTAATGTATTTCTTTTTCACTTTCATGTTTTATTTTTCGGTTTCACTAATAATACAGCATTACGTGTATAGTGAAATAATTTTCTTTTAAATTTTTCTAAACAATGTTTTTAATTTTTATTGTTCCACTATACACGTGAAAATAAAAGTGGAACATGAGATCCCATCTGAATTGAGTTTGCTTAACAATAGCATATGGAATCTTCTATTTCACTTTCCTGTTTCACTAATAATACGTTACCATGTGTATGGTAGAACAATAGAAATTAATGGAAATGCTGTTTGTAAAAATTCAAAAAGAAACTCTTGCACCATATATGTGAGGATGCACTTTGGATCCACTATTCTATTTTATGTTTCATCTCCTATTCCATTGAACGAATTCATGCCAAGCATATGACATCCATAACCCTGTGTATTTATAATGAAATATTTAGTACACTTGACAATGAAATAAGAGATGAATCACTATTTTGGAATATGAGATCTTGACCGGTGGTGAGTCACTGACGATGTATTATTGGTGGGATTAGGAAAGTGAAATAGTAGATCCCATTCGTAATAACAGAAACACTCCTGTGTTCTAAAAAATTATAACACCCCATATCGTTAGTTTAGTTTATGAAAAGTTCGTAATGTTTCTATCAATGTAGTAAAATTTAAGGGGTCTAACTATAATGTCATACAAATTCATTTATATGTTATGGTAACTGAACTAACGGAAATACGTCCTTTCTTATTTAATTTTCAAACCATATAGATATTTTTGTAATTATGTTCAAATTCAAGAGCTGTTGATCATAATTTAAGTTATACTTCATATTATAAATAAATATTAATTAAAATAGTGTATTTGAAGTTTAAAAGCTGGGAACTCGTGCAGGAGTGGAGCACATTAGAGGTGACATGTTTGTTAGCGTGCCTAAAGCCGATGCCATCCTTTTGAAGGTAAATAAATTTTAATTTTCATATTTTTCCAAGAATTAATTTTACCACCTAATTTTTACCTATTTTTTTGTTCTAGAATTACCCTTAATACTACATACATCATTCGACAATTATATTGATTTGTTTTAATAAAATATTCTCAATATATATATATATATAAATATTTAGTGAAAACCTCGAGTAATATATATCTTGTGATAAAGCATCAATTCCCTTTTTTTTAATCATTTGATTTTCCTAAAATGTATTTATTTGGGATATTTGATGTGTTATTAAATGGCATTTAATAAAGTGTTGTTATTATTATCCGTCGAATCATCTACCAATCTCAATTCACTATTATCACTTTATAATTTCATAATTATATATGTGGATATATTAATTATATTTTAGTAACACAAGAAGATATGTGCCAAGTATGAATAATACAAGGGTAATGTATGCAATTTACCCTTAATTTAAAATTTGGATTCTAACATGTAAAAGATCGGATTACTTAATTACATACTCGATTTGTCTACTTTTAAATACAAATATGTATAACTAAATTTAATTAATCAATTAAAGATCTTAAAATTGAGATCCGAACATTACCTCGGCAACGGGTCAAGTAATATAACGGGTAATCCTATTCTCTATTGCAGTGGGTTTGCCACGATTGGAGCGATTCAAGTTGCGAAAAGCTGTTGAAGAATTGCTACGAAGCGCTGCCCGAAAATGGAAAAGTCATTGTTGCCGATGCTATTCTGCCCGAGGACCCAAATAGCGGGCAGAGTTTTTTTTGGGCAACCCAAATTGACGTGATTATGTTAGCTTATAATCCAGGTGGAAAAGAGCGGTCAGAGAGGGAATTTGAGGCCTTGGCCAAAAAAGCCGGATTCAAACACCTTATCAAAGTTTGCAGTGCTTATGCTGATTGGGTTATGGAATTTCATAAATGA

>CbuCOMT11

CCCCAACCCACCACCACTTATATATCCCCCTCCTTTTTCACTTGCAACTTAAAGCTTATAATAAGCTCCAACAGCCATCAGATTACAGCTTATAAGTTCATAATCTTCTACTTCAATGGATAATCAGTCCGATGAAGAAGCCTGCTTATTCGCCTTGCAGCTAGCAACCGGTTCAGTGCTTCCAATGGTTCTAAAAACCGCCATAGAACTCGATCTCCTGGAACTTATCAAAAAAGCTGGGCCAGAAGCTTCAGCTTCTGCTTCTGAACTTGTGGCTCAGCTTCCAACAAACAACCCTGATGCAGCCAATATGATAGATAGAATTCTCAGGCTGCTGGCGGCACATTCCGTTCTTGTTTGCAGCCTGAAACCGCTGCCGGACGGCGGCGTTGAGCGGCGCTACTCCCTTGCGCCGGTGTGTAAGTTCTTGACTAGAAATGAGGATGGAGTTTCTGTGGGCCCTACTTGTCTCATGATCCAAGATAAGGTGTTGATGGAACCTTGGTAAGTGAACTATGTAGTAATTAATTTCTTGATTGGTTTTCTCACAAAAAGTTGTGCTTAAATGAGTTCGGAATTGATCCTTCGCCAGAATTTTGAATGGGTTGATTAATATAAGATGACTTGATGAATTCATACAATTGGAAGATTTTACGATCATTGAGAACTCTTTAACACTTAAGTCAGTGATCTAACTACGGAAATTAAATGGTACAATTGCCTTGAAGTTGATTAGTCAAATATTGTGTGGTTTTCCTTATTTAAGCTAACACACTCGATTCCTTTCTATTAACGGATTGTATTTCGAATTATTTGACTTGATTGGTTGGTTTTTAACTAATTTGGCTAAGTTTACGTTCATTTAGGGGTGTTAACGGGCCGAGCTCGAGAAAATTTGAAGGATTGAGGTTGAGATCGAGCTCGAGACCAATTAACCTATTTTTCGCTGCTCAAGCTCGAGCTTGGCTCATAGGCTCGTTTTGTTTATTGAGCTGAAACCCTTGTTGGAGCTCGAATTACTAATAATGAAAATTGTATCCATAGATTGTCGAATTTAAATGTATATACTATATATATAGTATTAACAGTTGGTCAATCAATTCAAATTCTCTGCTCGAACTCACTAACCTCAAATCTAATTCTATTTGAGCCGGTTTGCAACTTGATCCTCGACTCATTAGGCTTGACTCCATTGACACTCCTAAGTTCGTCTGTCTAGCTAGTTTTCTTAACAACTAAGCCCGAGATATTCTTGACCTTATACACATCTCGCACTTAATTTGTGTACTTTATTTCATTGTATTTATGTAAATATGCTTCAAATGATCTCCTTTGTTTGATAGGTATCATCTAAAGGATGCAATTCTTGAGGGAGGAATTCCATTCAATAGAGCATATGGCATGAATGCATTCGAATACCCGGCTAAGGATCCGAGATTTAACCGGGTTTTTAACCAAGCCATGTATGAACAATCCACCATATTTATGAAGAAAATTCTTGAAGAATACAAAGGATTTGAGGGTCTGAAATCCCTAGTGGATGTTGGTGGTGGAATTGGAGCATCACTTAAGATGATCATCTCCAAGTATCCATCAATTAAGGGCATTAATTTTGATTTGCCCCATGTTATTCAAAATGCTCCATCTTATCCAGGTACATATATGGGATAATTATTTGAGTTTTACTTAATTATAATAATATTCTCATAGTTTAATAAATTACAATAATACCCCTTATTGTTAATTAGTTTTGTTGATGGAAAACTTTTAATATTTAAGTCTATGTTAGATTTTCGAGGGGTCTAATTGTAATACTATACAAGCTCCGTTAAATTTTAAGTTAACTGAGCGGGTAAAATGCAATTTATCCTCTTGTGATAAGCGAAAGTAGTAATTTCCCCCTTATAAAAAATAAATAGGCAAAGCGCCCCCTCATATTTTTAGAAAAGAAGCTTAGTACCCCTACATAGGGGGCGCTATGCTATTTCCTAAAAGTGAGAGGTAATTACTACTTTCGTTTATCATGTAGGGGTGGTTTAAATTGCATTTTACCCTTAACTAAACTAATGAGAAGGAATTGTTCCATAATTTTTCAAAATATATGTGTATTTTTTGAATTAGATCAAACTTCAAGGTACCGTTTAGGTAATTTTTACTACTTCCTATTCTTTACATGAAGTTAATAGTCTTGATCTAATTATAATTTAAATTTTATTCCAAATTATATATAAATGTTAATTAAATAATTACTATATTTGAAGCTTAAAAGTTGGAACCTTGTCCAGGAGTGGAGCACATTAGTGGTAACATGTTTGTTAGCGTGCCTCAAGCCGATGCCATTTTTATGAAGGTAAATAAAATTGAATTTTCTTATTTTTCCAAGAACTAATTTTACCACCTAATTTTTACCTAATTTTTTTTTACTATAATTACCCTTAATACTAAATACATCATTCGATAATTATATTATTATTTTTAAATAAAATATTCTCAACAAATATCTATATAAGACAATCATAATCATTGTCTTAATCCACGTAATATCCCACATAATTTAATGTGAGCCCCTTTGTAATTATAGTGTAGGTACTTATAATGACATTCTTGATGAATTTCATCACGTCGAAGTCACGCGTAATATATACTTTTTGATAATAAATCAACTCCTTTAATTTCCTATATTTTTATTCGATCTTTTCCTTAATGATTTCATTTTGGACCTTTGATATTTTAGAAAAGCACTCGTTTAAAGTGTTTTGATGCATTATTATCTATCAAATTACCGATGACTCATAACCCACTACGTACCACTGTATATACACTATTTATACTCTAATTATTAAATTCACGAACATAAAGCATGCATTTGGGTTGTCAATATATCGGATTTAAATCAAATTAGAATACTTCGAACTTGATTTTTTACTTTTATGCTGAATTCAACTTGAGATCCTATAGGAATCGAGTCCATAAATACAAATTCTGAGTTCGAAAAAAATTCAAAATTCAATAAGAAAATCTGACCTAAAAATATTAAACAATAATTATATTGAGTATTTAATAAATAAAGTACTTAAAAATATAATTTTTTACATTATTCATTTAAGAAATTTCATTAATATTACATATATAAAGTCATTATTTCATTAAAAAAATATATATATTACAAGATCCAGATTTCGATACATATCAATTATTTAATTCTATATCTAATTTAATTATTTATAATCGAATTTAATTCATTGAAAACTCTAAAATTCAGATCTGATACAAATTGATTTCGAATTACCCAAAAACGGGTCAAGTATTCTACCGGGTAACCCGTTTGTCTATTGCAGTGGATTTGCCATGATTGGAGCGATTCACATTGCGAAAAGCTCTTGAAAAACTGCTTCGAAGCGCTGCCCGAAAATGGAAAAGTGATTATTGCTGAGACTATTCTGCCCGACGACCCAAATAGCGGGCCGAGTTCTTTACGGGCAGCCCAAGCTGATGTGATTATGTTGGCTTATAATCCAGGTGGAAAGGAGAGGTCAGAAAGGGAATTTGAGGCCTTAGCTGAAAAAGCTGGATTCAAACACCTTATCAAAGTTTGCAGTGCTTTTAATATTTGGATTATGGAATTTCATAAATAATGGGAAAGTTTGGGGGCTTATTTGAAAGTTTTTTTGGAAACTGGAAATGAGTCGAGTCGAATATATTGTTGTTTAGATTTGTTTGAATATTTATTGAGTTCTGAAAATTGGTTTTCGGTTTGTTCGGTAGTTGATGGGATCAGCATGAACATAGTATAATTGAGTTGAATATGAGTTGTACTCGAGTAGCTTGATTCATTTACACATATTTGATTATATTTTTAATCAATTGAGATGAGATTTTGATCTTGTCAAATTAGAAAATGGGTGCAGACGAGGGTCATTATGTTTAATGAACTGACTTTTGATCTCAAGTCGAGTATTAAATAACTTAATTTATTT

>CbuCOMT12

CTATGGATAGGCTTCAATGACAGATTCAACAGCTTTCATGTGTTTGATAGTGTGTTTGCTGAAGCCTGCGGCATTTAGGAGATGTGCCCATTCCTTATAAGTTCTCTCCTTCCCTTTTGTTGTTACTGTCATCATTGTTATATCCATTGCCAAACGAGCACTCGTATACTCATCGCCTCCTTCTTCATCTATTACAGCTTCAACAATGATCACTTTCCCTGTGTCTGCTGGAACAGCTTCTTTGCATTTCTTGAGGATGTCTATGCACAGGTTGTCGCTCCAGTCATGTAATATCCACTGCCATGAAAGGAATTTGAGAAATGATATATAAAATTATAAACTATTTTAAAATTCTTGATTCCTGTACATGGGACAAAATTTTGATATCATTATAAGTTGCTCAAATGAAAGTTCAAAAAGAATCACATGATCGTCCAATAATAATGACTTAGTTATCCATTGTGAACTTTCATATTTAATGAACATATTAAAAAACACATGAACATGCACACAAATAGGGGTGTTAACGGTTCGAATTGGATCAATTTTGAAGCAAAAATCTATCCGATTCAAACTATATAATTTTTATTGGATTGGTTTGGTTCAATTTAGTCTTTTAAATAAATACAAACCAATTAAAGTCAATTTGAAATGGATCAGTTTAATCGATTAGATAATATAACAAATAAAAATACATAATTCTATTAAAAAACTAATTATTAATTTAAAAGTTTAATTTGGATTGAATCAATTTTCAAAATTCAATCTGCAAACCAATCAAACTTTTCCAAAATTATATCCAAACTAATTCGAAATGAATCAAGTTTAATCAATTTTTAATTTATTTTAAATTAAATGATCAATTTTGTTCAATTTGCCGTGGATATGAACACCCCTACACACAAACACGTACCATGAGCATAACTGCATCAGCTTTTGGAACACTTTGAAACATGTTTCCTCCAACATGCTCCACCCCATCAATGGCGGAAGCCCCGGCGATCACATCCGGGAGATCAAAATTAATCCCTCGAATCCAAGGAAAAGCCTTAACCAGCAGCCCGATAGCCATTCCTTCATGACCACCAACATCCACCAAACACCCAATTCCTTCAAACACCTCCTGACAATAATCGATAAGCGCCGTCGTCGCAATCTTAGCATGGTAAGTTAAAAAGTCATGAAACAGCTTATCATAAGCTGGATCAAGTTCATAATTCCACATCATATCCTCAGAACAGGCAAGATTATCCAAACCAGATCCCTTTCCAGCTTTCAGATCATTTGCAGTTAAGCCAATATATTGCACCTTTGGAGGATCCGCTTGGAGAAGTACAAAAGGGGCCATTTTATCTCTTGTGAGGAGGCGGGAAAGGGCAGTTTGGGAATAGTAAGACGATTCTGGATCTTGGCTTTTGCTTAAGTTAAGCTCCTTTTTGAAGATCCCGTTGTGAGTTAAGAAACGGAGGAGGCGGCGGAGAGCGGGAATGGGGCAGCCGACGGTGGCGGAGAGTTGAGAGAGTGTCATGGGGCCGCCGTGATTTTCGAGGACGTCGGGTAATCCTAGTTCGATGGCGCACTTCACCGCTCTCATTGAATTAAAACCCAAAGCGTAGTTCCATATCTGCACTCGAGCTTGAGCTTCTTCGTCCATCAATGGCTCTGAATCGATCTTCCCTAATTGTGAGGTAAACCTATAAGAAAAATGGACTCAGGCAACTTATTTATTTCCACAAAGCTTCGAGAGAAGAATCTGATTATATTTTTCTTTTCCTTCACATGACACCTCTGATCACAAAAGAACAACTTCGTTTTGCGGAGTCTTTGCTGCCAGTCATGATGAAAGTAATTAAGAGACAGACGAGGTTTGTTCTAATTTTAAATGTCTTGAGTTTGATTTCTAATATGTGAGTTCTATAACTTTCACTAAAAAAAATAAGTATCTACTATCGAAAAATTGGTACGAAATGAATTTCGTAGCAAAGACTATAAAAATGACAATAATTTTT

>CbuCOMT13

CTATGGATAGGCTTCAATGACAGATTCAACAGCTTTCATGTGTTTGATAGTGTGTCTGCTGAAGCCTGCGGCATTTAGGAGCTGTGCCCATTCCTTACAAGTTCTTTCCTTCCCATTTATTGTTACGGTCATCATTGTTATATCCATTGCCAAACGAGCACTCGTATACTCATCGCCTCCTTCTTCATCTATTACAGCTTCAACAATGATCACTTTCCCTGTGTCTGCTGGAATAGCTTCTTTGCATTTCTTGAGGATGTCTATGCATAGGTTGTCGCTCCAGTCATGTAATATACGCTGCCATGATAAGAATTTAAGAAATGATATATGATAAAAATATGCTACTTTTATTCTTTTTACGCTTGTTCATATTCGAAAAGAAAAAAAATATAATAATAATAATAATGAAGAATGTAGAAGATTTTTGTGTTCGTTGTTTAGTTTAATTGTGTTTAAATATATCGTGAGACATTGAAATCTAGTCAATTTGATATCATTATAAATTTCTCAAATGAAGTTAAAAAAGAATCACAAGATCGTCATTTCCATACACGACGTCCAATATATTTAAAAAAAAAAAAAACACATGAACACCAACACAAAAGGGGTATTCACGATCCGAATTGGATTGATTTGAGAGAAAATTCATACGATCCAAATTAAATAATTTTTAGTGGATTGATTTTGTTCGATTTGATTTTTTAATAAAGTCAAACTAGACTTGAATTGAATTAGCTTAATTGTTTAGAGAATATAACAAATAAAAATATGTAATTCTATTAAAAAATTCATTATTTATTAATGTAAAAGTCCACTATATACCTTTATTTTGAACATTAACACATGATCAATGATATATATATTTTTATATAAAACATAAAGTTAAATTAGTAACAAAAGTTCAAATTAGCACTAAATGACTTTTATAGTCTGGTTTTCATATTATCATATTCATAATGTAATATTATTATGTTATAATATATACTTCGATTCGATTTAGTTTGAATCAATTTTCAAAATTCAATCCACAAACCAAATCAAACCGACCAATTTTTTTAAAATCAAATCCAAACTAATTCGAAAAAAATAAGTTTTGATCTGTTTTCGATTTGATTTGGATCCGATGAAATTGTTAATTTTGTTCGATTTAATTTTGTTGCATGCAAACACTTACCATGAGCATAACTGCATCAGCTTTTGGGACACATTCAAACATGTTTCCTCCAACATGCTCTACTCCATCAATGGTGGAAGCCCCGGCGATCACATCTGGGAGATCAAAATTAATCCCTCGAATCCAAGGAAAAGCCTTCACCAACATCCCCATGGCCGTCCCCTCATGACCACCAACATCCACCAAACACCCAATTCCTTCGAAGACCGCCGGACAGTTATCGATAAGCGCCGCCGTTGCAATCTTAGCATAGCAAGCAAGGAACTCGTGAAGCAGCTTATCAAAAGCTGCATCAACTTCCACATTCCAGATTATATCCTCAGAAGGAAATCTATTATCCAAACCAGATCCCTTTCCAGCTTTCAGATCTTTTGCAGTTAAACCAATACACTGCACTGCTGGAGGATCCGCTTGCAGAATTACAAAAGTGGCCATTTTATCTCTGATGAGGAGGCGGGAAAGGGCAGTTTGGGAATAGTAAGATGATTCTGGATCTTGGCTTTTGCTTAATTTAAGCTCCTTTTTGAAGATCCCGTTGTGAGTTAAGAAACGGAGGAGGCGGCGGAGAGCGGGAATGGGGCAGCCGACGGTGGCGGAGAGTTGAGAGAGTGTCATGGGGCCGCCGTGATTTTCGAGGACGTCAGGTAGTCCTAGTTCAATGGCGCATTTCACCGCTCTCATTGAATTGAAGCCCAATGCGTATTTCCATATCTCCACTCGAGCTTGAGCTTCTTCGTCCAT

>CbuCOMT14

ATGGATAATAAGTCAGATGAAGAAGCCTGCGTATTCGCCTTCCAGCTAGCAGCCGGTTCAGTGCTTCCAATGGCACTATACACCGCCATAGAACTCGATCTTCTGGAACTGATCAAAAAAGCTGGGCCAGAAGCTTCAGCTTCTGCTTCTGAACTTGCTGCCCAGCATCCAACAACTAACCCTGATGCAGCCGATATGATAGATAGTATTCTCCGGCTGCTGGCGTCGCATTCCGTTCTCATTTGCAGCCTGAAACAGCTGGCGGACGGCGGCGTTGAGCGGCGCTACTCTCTTGCTCCGGTGTGTAAGTTCTTGACTAGAAATGAGGATGGAGTGTCCGTGGGCCCTCTTTGTCTCTTGCTTCAGGATAATATAACCTGGGTACGTAAGTGATCTACAAGGGTGTAGAGGTTAAATTAATATAAGTTGACCTCAACGGTGGATATGATTGGAAGATATCTGATTTCAATTCCTTTGATACTTAAGTCAATAACTTGATCAAAATCGTATCCATGACCTTATTTAAGCTAAGCCACTTAATTTCTTTTTATTAATAAATAGTATTTTGAATTATTTGGCTTGGTTGGGTATATCTAAGAACAAAGTTGTACTTCACTTGGAGGTATTAATGAGTCAAGCTCGGGGATAGTTGAAGGGTCGAGGTTAGAGCAAGCTTGAGATCGATCGGTAAACTTATTTGTCATCGGTTTAGATCTTCTATTTTAAAATAATATTTCATTTCTTATTTTATGTTTTAAATACATGTCACGTGAATTAATTATTTTCTTATAAAAATATTTAGTAATAAATGTAATATATATGACATGTATTTAAAGGCACATAGGAGATGAAAAACCATTTTGAAATAGATGATCCAATTTAAATTTTCATTACTTAAGCTTGAGCTTGGCTCATTAGCTCGTTTTGTTAATTGAGCCAATTTAAAAAATCATGTTGGAGCTTGAATTATTGACAACTAAAATTGTATCTATAGATTGTTGAATGTTTTCTAAAATTTATTTAACTTTTAGAATTTAAAACTCAAATTTTGCATATATACATATCAAATATGTAGTATTAAAAGATGCTCAACTGATCCGTAAGTTTATGAACAACATACTCTGTACTTTACTTCATCGTATTCATGTAAATATACATCAAATGATCTCCTTTCTTTGATAGGTATCATCTAAAGGATGCAATTCTTGAGGGAGGATCTCATTCGATAGAGCATATGGTATGAATTCCTTTGAATACTTGGCTAAGGATCCGAGATTCAACCGAGTTTTTAACCGAGCTATGCATGAACCATCCACCATAGTTATGAAGAAAATTCTTGAAAAATACAAAGGATTTGAGGGTCTGAAATCCCTAGTGGATGTTGGTGGTGGAATTGGAGCATCACTTAACATGATCATTTCCAAGTATCCATCAATCAAGGGCATTAATTTTGATTTGCCCCATGTTATTCAAGATGCTCCATCTTATCCAGGTACATGTACAGGATAATTACCTCAACATGCCCTTATTGAGTTTGGTTTAATAACAGAAACACTCTTGTTTCTTAAAACATTATAATACGTCATACCGTTAATTTAGCTCATGATACGCTTGTAATGTTTCAATCAACGTATTAAAATTTAGGACTAAAATTTAGGCATCTTCCTGACCTATTGGGTCAATTGCAATTTGCTCTCTAATTTTTTTAAACGTGTGATTACCACCCAACTATTGCAAAATGTTTTGTTCCAATCCCTAAAACTGGAATCTGACAGAATATTATGGAAATATTTCATTTAAAGCCCTCCATATGTCTTTATTATAAGTAAAATGAGGGTATTTTAGTAATGATATCATATCTTGAAAAAATTAAAGATAAATTAATCCTAGTTTTGCGTAATTGTCTTAATTTAACCCCAATTTAACACTAATTTTGTTGTGATGAGATATCCATATAGAAACGCTCTTACTGTACTTGCGCCGAAGATGTAGGAGGCTTTGAATGGACTATTCCGCCGGGTTCGAATTTTAGGATGTGAAATCAAACGTTTTGTAATGCTTAATTAGGGGACTTATCGCACATTTGAAAAAATTAGGGGGCAAATTGTAATTGACCCTATAGGTTAGAGGGTTGTTTGCAATTTACTCTAAAATTTAAGGGGTGTAACTATAATATTATACAAGCTCCAGGGGTTGTTTCGTTGTTAATTTTCAAACTACCCGGATATTTTTGTAATTATATTAAAATTTCAAGGGGGTATTGAAGTAATTTTGATCACTACTTTCTATCCTTCACATGAAGCTAAAACTCTTGAGTTGATTATAATTTAATTTATATTCCCTATTATAAATAAATATTAATTAAATAATTACTATAGTTGAAGCTTAAAAGTTGGAACCTTGTGCAGGAGTGGAGCACGTTAGTGGTGACATGTTTGTTAGCATGACTAAAGCCGATGCCATTTTTATGAAGGTAAATAAAATTGAATTTTTCATATTTTTCATTTTATTTTATCTAAGTTTTTTATACTTATACTTCATACATCATTCGACAATTATATTAATGGTTTGTTGTAATAAAATATTCTCAACAAATATATATAAAGACAATCGTAGTCATGGTCATTCATATCACATATAATAATATAATCTATGTATTATCACAAATCAACTCATATTTTTTTATATAATATGAGATAAAACATAGGGTGGTAATTTTCGACACAACACGATAACAAGACACGAACCAACACGATATTATTGGGTTCGTGTTGGAGGTTATTGGATTGGGTCGATATTGGATCGACACGATAAGTAACACGAAAATTTTGGGTTGGTTCGGGTTACACGATAAACACAATAATTTTATTGGCTAACACGTCAACCCAAACCAAACCAACCCATCCCAATCCAAAATTCTAAAAAAAAAAAAAAAGAAATAAAAATACAAAGTTTTGTCAATTTTTTCAAGTGGTAATAGTGCAAGATTAAACAAAGTTATCTTTTCAAAATATTTCACTTTTTATATTTTTGAAATTGTATTAGGGTTTTTAGTTCAAAATTCTTTTTCAAAAGAACTGAAAACCTATTTCTGTAGATTCATTCTCTAAACAACAACGCACTTGAAACAAAATACTCACTTCTACGGTACTACCAGACACTGCAACAACTATTACTTTCTCCTTCCATATTATTATTTGATAATATTTCTTTTATTTTTCTCATTTTTTTGAGTTTTAGGGTTTTTAGAAGAAAATGTGAACATGAGAGAACTTACTTATTTCTTTAATTTTTCTCATTTACTTAGTCTTGTTTAGATGATTTGATGTTGCTTTTGAACAATTTATTTATTTTGTAATAGATTAATGACAATTATTTACTACTTAGTACTTGACAGTTTGTTAATTGGAACTAGTGATTGAACATGAGTTGACACGATAACTTTTGGTTTGAGTTAGGGTTGAGACTTATTGGGTTGAGTACTATTTGGGTTGGATTCAGATTGAGCAAAATACTATAATACTCATACCTCAACATGACATGATTGACACCCCTAATAAAACATCAACTCCTTTTTTTATTCATATTGATTTTCCTGAAATATATCTATTTGGGATATTTTGATGTATTTTTAAATGATATTTAATAAATTTCTCTTATTATTATCCTTCAAGCTATCAATTATTCACAACCCACTACCATCACTTTACAATTTCATAATTATATACATAGATATTTATATACCGTCAAATTTGACTCGAGATCCTGTAGAATCTGGGTCCTTTATTAGTCACAATATCACATATATTCCACTTAATTTATTGAAGACATTTTATATATTATAGTTATTAATTAAAATTCAATTCGAATCTGTAAAAAATCGGATTACTTCATCCATTTCCAAACACAAACATAAATAATTGAATTTATTTAAAGTTGTTAAAATTCAGATCCGGATCATTACCTCCAAAACGGGTCAAGTAATCTAACGGGTAACCCGTTTGTCATTTTTGCAGTGGGTTTGCCACGGTTGGAGAGATTCACATTGTGAAAAGCTGTTGAAGAATTGCTACGAAGCGCTGCCCGAAAATGGAAAAGTGATTATCGCTGAAGTGATTGTGCCCGACAACCCAAATGGCGGGCAGAGTTCTTCATGGGCAGCCCAAGGTGACATGACTATGTTAGCTTATACTTCAGGTGGTGGAAAGGAGAGGTCAGAGAGGGAATTTGAGGCCTTAGCTAAAATAGTTGGATTCAAACAGCTTATCAAAGTTTGCAGTGCTTATAGTAATTGGATTATGGAATTTCATAAATGA

>CbuCOMT15

ATGGTGCTAGACGAAGAAGCCCAAGCTCGTGCAGATGTCTGGAAATACGCTTTCGGATCTATCAACACCAAAGTAATGATAGTCGTCGTCCAGCTCCAAATACCCGATATAATGAAAAAACACGGTGGCGCTATCTCACTCTCCGACCTCTCCGCCGCCGTGGGTGTCCCTGCCGATAACCTCTACCGCATAATGAGATTCTCAATCCATCACGGCATGTTCAAGAAAACAGAGCCCCCACAGAGAAAAGTCTCAGACGACGTCGTATACTACGCTCATACGCCGCTTTCTCTTCTTTTAACAATTGACAACGTCGGACCCTTCATTCTGCTGCAGGGGGCCGGTCCCCATGGAAACTTTGGGGGCTTAACTGTGGCTGCTTTGAAAATCGGAAATCGCCCCGATTTTAAGACTCTCAACGGCAACAGCAATGGAAACGGCAACGGAAACGGCAATTGGGACGATCCATTCTATGCGACAAAGGTATATACGGACGCTATGGCGTGCCACGCTAGGGTGGCAACATCGGCGATTATTAAGAACTGTCCAGAAGCTTTCCGAGGAATTAGGACGTTGGTGGATGTCGGCGGCCGCCATGGGATGGCCCTTAGTATGTTGATAAAGGGATTTCCATGGATTAAGGGGATTGCTTTTGATCTTCCTGAGGTTGTGGCTAAGGCTCCTCCTGTTGATGGGATTCAGTTCGTTGGAGGGAGTATGTTTGAAGCTATCCCAAAAGCTGAAGCAATTATGCTCATGGTATGCAATTTATTTTTGAGTTACTTTAACAAAATATAACTAAGTGATAAAAAATTATCATTAGTAACATCTATTAAGTGATAATAATATGTTTTAACCGACAATAATTTTTATTTCGTCACAAAATATACTTTAGTGAAAAAAATTAATATTTTAGCCTTCAAAAAAAAAAAAAAGAAAGAAAAGAAAAATTATTATTTTAAATTGTCATAATTCACAACAAGGTATCATGCCCATATCTAAATTTGCTAGAGTTCTATTTCATCACAAGTATCATCTATTTACGAGGAAACCCTTTTCATCACAAAATTTTCATAAATAAAAGTGTTTTCTCATACTATTTTATGACAGTGGATATTGCACGATTGGAGCGATAAAGCTTGCATAGACATCCTCAAGAAATGCAAAGAAGCCATTCCGGCAGACACCGGAAGAGTGATCATCGCAGAAGCAGTCATAAAAGAAGACGAAGAAGAAGATGAGTACACAGGTGCCCAATTGTCGCTGGATATGATAATGATGGACCTACATATCGAAGGCAAAGAGAGGACATACAAAGAATGGGCGCATCTTCTCAAGGCAGCTGGCTTTAGCAGACACAACGTTAAAAATATGAAAACTCTTGTCTCTGTAATTGAGGCCTATCCCTAA
