# Supplementary material for: Genome-wide analysis of the Catalpa bungei caffeic acid O-methyltransferase (COMT) gene family: identification and expression profiles in normal, tension, and opposite wood
Source: PeerJ. 2019 Mar 14;7:e6520. doi: 10.7717/peerj.6520 (PMC6421059; doi:10.7717/peerj.6520)
Supplement: File S6 [file peerj-07-6520-s008.docx]

>CbuCOMT23

CATTCTGGAATTTGATGGATGTTTGTTTATATTTTGAGTGTTTAGTAGAAATTATTTTGAAAAGTTAAATAAATGTTGAT

AATGTTTTTAGAGGAGATAATTTTTTTGGAGTAACGTCTTAGGTCCATCTATATACAAATTTATGAAATACGATACGCCT

GCTTTGGAATCATCTTTGTATTTGAAGGTGTTTTTCTTTTGCCAGTCGTGTTATTACATTAAATACAAATTTTGCATGCA

GATGAAGAATTTCTCTTTGTGTTATTTTAATATTTATTCATGTTTATGGATACTGGATTCTTGAAATAACACTTGTCATA

TTCTGTTTACCAGTGAAATATTTGCTTACATTTCTCGTAAATCATTGTTGTGATATTGCAGTTTATGGTTAAAGATCAAA

TTCCTTAAGCCATTCATTTTTCTTTATTCCATTCTTCATATTTTTCTAACATGTTTCAGTTTGAAGTATCTTAATTATTA

AACAAGGAACAATAAGTCATTTCTATGAATGGTTAAATTTGGACATCGATAACAAATGTAGCTAGGTTCGGTATATCTTG

AAATTGTTATTCTATTGTTTCAATTGATAATGACATAACTGAAGTCTTAAATTTATGATCTTAGACTTGTCCCTATTCTA

CAACTTTGATATATCATAATTTTTTTCCTTGTTACGATTTATTTTTGGCATAATTACCTCAACGTGGCTTAGGGTGTGAC

ATAATTACAATAATACGTATTATCGTTTGAACAATTGCAATGACACTTCCTGCTATTAATTCATATGATGACAACTTAAC

GGAGTATAACTATAATTTCCCAATCATGATGAAGTGTTTTTATAATTTTTCTAACAACTAGTAACCCACTCACGCAGTAC

AATATATAAAATATATTTTTTTGTGTAAAAGTACAATTGATTTGCATGTGAATAGTCTTGCTATGTGCTTATTAGATCAA

ATTTGATTGAGGACAATCCAAAACTGAATTTGATTACGATGTTGATCATTGAATCTATCTTGCGATTCAATAGTTTAATA

TTAAAAATGTTTATAATAAAGTTACTTTTAATTAGATGAGTGTAACTTTTTTAGTTTGGTTAACAAACAAAATATTTTTG

TTTGGTTTAACAAGGTCCTGATTAGAATATGTCAAGTTTTTATTTTATTTTATTGGGTTGTTGTTGTTAAATATTTTAAG

TAAAGGTTAAAATACTTCATAACAAGGTTCATCTTTACAAGCAAAGAAGAACATTTTTTGTGCCAATATCATATTATTAA

TGAACATTTCTCTTTGTTATTAGAATTTTTTTTTTAATCTCAAATTCATATCTAATTGTATAGAAAAATTTCAAAATTTA

TCACAATTAAATAATAGTTAGATAATTGTATAATTAAACTGTATAATTGTTATTACGAGTCTAAAATTTCAAAATTTATT

ATATAATAGTTAATCCCTTTATAACTCTATTGAATTATAAAGAAAATAGTTTTTCAAGTAATCGATATATATTTTTGTAA

GATAATTTGGATACAAATAATTTTTCGTATCTTGAAGCCTATCAGGCTTCTTTTTTCTTTTTTTTTTTAGTTAGTAACTT

GTACATTATTATTTATTATTTAATATTAATATACCCCCCCCCCCCCCTCCCTTTTTGATGTGCATCCCCATTCCCATTTT

CTAATGGTGTTTTTTATTTTTTATTTTTTTATTTTTTTATGTGCATCCCCTTTTTCAATGGTCATTTCCACATTATTTAA

TTTAGATTAGACCTAATTTGGCATAGGCCAGTGTGCATTTTTATGAATCCTGATTGTATCTTTCAACCGAACCTATTCAT

TTATTAAGATATTTCCTTTGGTGAGGGGGCAGTTTGGTCAAATTCAAAAGCCCTACTCCATTTTATTAACTATATAATTG

TCTGGAATTGCTTAATTTCTTGTACATCAGAAGCGAGAAAGAAAG

>CbuCOMT22

AAATTGAATATAAAAACAAGCAATTTGATCCGTTAAAAACTCAAAATTATGATTTTGTTCTTTAGCCAACAATGGAAGGG

GGGATTTTTTTTTTTTTTTTTTTTTTTTTTTTTTTTAAGAGTTTTAGTTAGTAAAAGACAAACATTTCCTTATACTTAAT

GAAAAAATAGACGGTCAACTAACGGAAGGGATGTATCTGCCAAAATCTAAAAGGTTAGGACGGTGACTGCCAAAAACAAA

AAGTTCAGGGGGTGTATCCGGAAATAACCTAAAAAGAAAGGTACAAAAGTTAGTTTTCATACCAAATTTCATAATTCCAA

CTTTATCCTTCAACTACCATCACCATCTATTTTTTTAACAATCCTCATTCCTACATTATTAATTTCAAATAATATTTAAA

AAACTAAATAATAGTTTTATGAAAAAAGTATTATTGTAACATTGGACAATGTTTAGTATAAAAGTGAAAATGTCACTAAT

TCTATATAAAAGATAATTTTTTTTTTAATATTTTTATCACAAAATACCATTTTTTTAAATTTATTTTAGATATAAGCAAG

GACTGCGTGCAGAAATTACTAGTATATAAAAAGCGAAGCCTACATTAAATGTGTACCGATGGCTAGTATAAAATCTAAAT

TTTCCCTTAAAGCGTAGACGAGATCTCCTATTTCATTTTCGTATTCCAGTTTCCTACTCCACCAATAATACATCGCCGAG

TGTATGGTGAAATAATAGAAATTAAAAATGCCAATTGTAAAAAATAAAAAGATATTGCTTCACCATACACACAGCGATAT

ATTATTAGTGGAACAAAAAAATAAAACACGAAAGTGAAATATGAGATCCCAACCCCTTAAAGTGAGGGCATGATGGGCAT

CTGCCATGACAATAATATTTTTAGAGAAAAATATTTTTACCGCAAATCAAGTTGGCATGAAATTTTAACCAAGATGATTA

AAGATTGATATTATCTCTCGGCATTATATTAAATTGATAATATAATATTAAAGATGTCTAAGTGGACTAAAATAATGGAT

AAAATGCAATTTATCCTTGTGATAAGTGAAAAGTAATAATTTACCCCCTTTAAAAAAAAAAAATAAGCAGAACGACCCTC

ATGCTTTTAAAAAAAAAACTCATTATCCCACTGCATGAGGAGACGTTCTGCTTATTTTTTAAAAGCACGAGGAGGTGCTT

GCCTATTTTTTTTTATAGGGATAAACTGCTAATTTCGCTTATCACGTGGGGGTAAATTGCATTGTACCCCTAAAATAACC

ATTGAATAACTATCATTCTTTATTATAATGAGAATTATTTCTCATAAATCTTATTTTTTAGTCATAAATTTTATTTGTCC

ATATTATTATATTTATCATCTTGCAATTTATTTTGCAGCCCAAATTTGTGTGAACACTTTCAACCATACTTCCATGTTTA

ATTTTTATCTAGCTTTGACTTTTAGATCCATTTATTTTGGTAGATTATATAGTATCGTTCTAGCTCTTGGTTTCCTACAA

AATCCAATTCTCCATTCCTACAAAATTCTAGCCCTAACTTAGGAGTTGACTTCAATAACTTGCATAAAATTGCATAAATA

AGCTCTTGAAATGTTTGGATTAAAAAAAAATAGAACTTAATTACAAGATGTTAATAATAACAATTTTATAATTAAAATGA

TTAAAATTTAAGGAACTTGTTCATAAAATCTTAAAAATAAGTTACAGGCACCTTGCTTTTTTAAAAATTAGTTATTTTGA

GAGATTATAATCTCTTTCTTTAAAAATTACCAAACACATTTTAACATTTTATTTAACATTTTGAAAAACTTATAAACTCA

CCCAAACACCCTCTCAGTGACTTCTGTATCATCTGTTCCAATTCATGTTTTTTTTTTCTATTTTATTGTACGAATGCATG

TCAAGTATAATTATTCATAAACTTGCCGTGTATTAATAATATACGGTACGAGACGAAATATTATATTGGAAAAGAGGATA

ATGACTCTAAGTCCACCAAAGTTAGGTACAGCAAAATTATGATAATCAAGAAGCTAAAACTCTCCATAAATTAAAGCTAG

AAGCTAAAAATCTCTACTATTAATTAGCTA

>CbuCOMT21

AAACAAACATAAATTATATATTAAGATAGAAAATTAATTAAATAGACCATAAACTATATAGTTTTATATATGGGTTATAT

AGGAATTTTATAACCATAATACATAGCTCATTTATAAATTATTAATTATGATCACTAATCATTACGATTAGTAAGTTTTC

ATCCTATACATAGATAATTAATTATGAAATCGAATCACACATTATTACTATTTAAATAAATGACGTTGTGGCCGCGGTCC

TTTGGGTGTAGCGGCACCGTCTCCTCTTTCCACAAGAGAGGTACAAGTTCGATTTTCACCAAGTGCGTGTAGGCTTTTTT

CTACCATATGGGTAGCTTTGAATTCTACTGTTGAGTAATTTGTTTATACTTATTAATGTATTTGATATTTGAATGTATTT

AAATATTTATGTTAATGTATTGATAATATATCGATTGAATGTAATAATTTTAAAAATAAATGAATAAATGACGCCGTGTA

AAAGTGAACAGGTTGGTAGTAAGGTCTTGTGGCTACAAACAAATAAGTCGTCAATTCGAAATAATGTCATCACATTATAG

TTACTCTTTTGAAAAACATAGAAACCAATCATATTTTCCAAATATTCTGTTGAGAAATCAAATCCAAATTTATTTCCAAT

AACATACGTTCAACACTAATTTTATAGAGTCAAAACAAATAAATAAATATATATATATATATATATATATCATTTACACA

CCAAAAGTTGATAAATATTTGAACTCTAAAATTAAATATAAACAAAATGCTCTTAATTAGTATTTAATTAATTATTAAAG

TTCTCTTCTGCCAAATATATTATTAATTTGAATGGTAACTAAAATTAGTTCTAGGTTACGTATGTATAAAATAATTAATA

TATATTTATTTCTTAGATATTAAAGTAATAATTAGAATATAATTAATATATTTAACGTGTGTATATATATAAACGCACAC

AATTTAATATCAAGAGATAAATTAGTGACCAAATTATGCTCTATTATTGGGTTATTGTTGGTTATTTCAATGGGTCAATG

TTAAGAGGTATTTGAAGTATAATACATGAAAACGCATTATTAAAATAATTATTTGAGGTCTTAAGGAGTAAAGGTCTCTT

ACTTGTAGATAATTTGGGAATATAAAGCTGAAAAAAAAAATTATTATTGAAAGTAAAAAAAAAAAAATTCATCACTAGAA

GGACGACGGGACAAGAATTATGTATGAATTATTAATTACTGCCATTAATCCTTAATAAAAAGTCAAAAAATAGTACTCCA

TAAGTCTAAAGAAAATAAATATTTCATGTTGTCTCTTTAATAGTAAGCTCTTATATATGCTGCGAACAGATAAATTGCGA

GTCCAAATCTTTTATTATTAATATTTGAATAAATGATGCATAAAAAAGGTGGGCAGCTTGAGAGTACCGTAGCATATATA

TTATTAATATTTGAATAAATGATGCATAAAAAAGATTGGCAGCTTGATGTCTGCGAACAGATAAACTACAAATTTAAATC

ACATATTATCAATATTTGAATACTCCTACAATAAAATGTTTTTATCACATAAGAAGTACTCTTTTGGAAAACATTAAACC

AATCATATTTTCAAAATTTTCTTTTGAGAGATCAAATAAAATATATTTCCATGCAATATCATAAAGTTCAACACTAATTC

TATAGTATCAATCCAAATATATATTTCATTTTCACACCAAAAGTTGATAAAAAGTACTTGAACTAAAAGTATTGGGAATA

TTATAAAATAATCTTGGAATATAGACAAACAAGCTCATTATATTATTTGACATAATTATTGAAGACAACAAACCAACTAA

TCAATTAAGTATCTATTAATAAATAGTAACATTAATATTTTTAGTTGATGAACACGACATTTTATCATATCATTTAGATT

CGAAAAAGACGATTATAATTTAAAGTTATTTGATAGATTAAAATACCCTTTAAGTTATAGACAATCGACGTTCGAATCTG

GACGAAATATACGCAAATATTTCATCCAAAATAAAATGATATATAAGCATTTTTCTGAAAAATTATATAATTAATTGAGT

CGAGAATAAGTATAATAAATATAAGAATAAGTCATAATTCCAATGGCACAGCGTAATTATCCCTATCGCTACGGGGAATC

TCCAACC

> CbuCOMT 20

TTAGGGTTAGAATATGAAAAGCTGTTGAAGAATTGCTACGAAGCGCTGCCCGAAAATTTTCCATCAATTTATTTTCCTAG

TAACCAAATTTTCCCTCCTATTTTTTTTTTTTTTTCCATCCTAAAATCCTGGAACCAACCACAATATAAAACTTGCTCAG

TTGATTCATAAGTTTATGAACAACATACTATTTGCTCGAACTTGATTATAAAGCTCGAACTCGTGGAGCTCGATCGAGTT

CTATTCGAGTCTCGACTCGCCTTTATGTTAACATTTTTTAGTTTTCAAATGTTTTTTTAAATAATATAGAATAATTTGTC

AATTTTTTAATGTTATTTAATATTATTTTATTTATTTATTTATTTATTTATTCTATTTTTAGTATAATTTTGCTCATTAT

TATTATTATTATTATTATTATTATTATTATTAAAAGCATATTAAATTATATACCCTAATGTAATCTTTATTGATGTATTA

AACATGGCCGAAACTTTTTTTTTGTTACAAGTGTATTATAAAAATTTGCACAAAATATTATTTGAATTTCTTATTTCATT

ATATCATAATATCATGTTTGATAAAAGATATTAAGGTCAATTATAGAATGTAAGGCACAAGGGCATAAAAGTCATTTAAC

CACGCAACAACACTTAATATTCCCTAATACACCATATCAAACAAGCAATTGAAAGGAGTAAAATAATTCATTTATTCTCT

ATATTAGCATATCAAATATGCAACTATGGAATGTAACATTCTTTATCAAACAAGGCCTAAATCTAGAGTGTCAAACCAAA

TAGAAATTTCATTTTCACATCAAACGTTGATAAAAATAGAAGTTTCATTTTCACAATTCAGTTGTATATTTTATCACATG

ATCCCCTCAATTTCGGTGGAATATCTATGAATTTTCATCCAGTTCGAACGTCGATTACAACTTAAGAAATTCTGTTTATT

TGAAAGTTTAAAATATCATTTTCTACTTAACGACCCTTATTTTTCAGATTCAAAATTCATATCTTGTATCTTTTCACCGA

TTTATAAAAAGTGCCAGTTTTGCTAGAAAATTAACGTGATACCAAAAAATCGACGAAAAATTTAATTTGACACGACCGAC

GTGTCTCATTATTGGTGCCATATCAGATTTTTTGACGAAACAGACACCTTTTTTTTTTTTTTTGTAAATTGGTGAAAAAA

TACATGACAAGAATTCAAAATTCGAAATATACGGGTTATTAAGTGAAAAATAACATAATTATAAAACAAAAACTGCTTTT

TTTATTTATTTATTATTATTTCCATAAAATATCCCTTCTGAGATGAAGCGATTTGATAAAGTATTACTTTTCCAGAAGTT

CCCCAGACTTACATATACTCCACCTTTTTCACTTGCAAATTCAAAGCTTATAATAAGCTCCAAAAGCCATCCAATTCCAG

CTTATAAGCTCATAATCTCCAAACCCTACTTCA

> CbuCOMT 19

CTAAACAGGTAATTTTTGGAACTCATATAAAAAGAGAAGATTTTGGACTAAAGAAAGAGATACAAGAGAGATTAAAGAGA

TAAAACAATGTTGAACAAAAAGCTGAACAACAACAAAACAAAAAGTTATCAAAGTGTTCAAACGCAAGAGAAAATAGAAG

ATATTCAAAGTTCAAAATCTGGAAAAAGCAACTCAGTTTTTCAAGAAGATCAACCCTCCAAGTTACTAAGCTGAAAAAGT

ATTTTTAGCGAAGTAGTTATATTTCAGCGAAGTGACTTCGCTCCAGCGAAGTGGCTTCCTCCTAACTTCGCTAAGCTAAG

TAACCAGCGAAGTACTTGTTCAACAGCAAAGTGACTTCGCTAACATCGAAGTAGTCTCATTAGCAAAGTGAAGTAAGCTC

AGCGAAGTGGTATCGCCAGCGAAGTAAAGCCTTCACTTCGCTATTGCTGATCAGCTTAATCCTCTTAACTCTAATCATCA

TTTCAAATATCACGCAAATTTATAAAAAAAGAAAAAAAGTCTATTCACCCCCTCTAGACTCTTTCATCACTTGGGACCAA

CAACGATTCGAAAGTCCATCCTATTGGAGTGCTAATTTAGGCCGGAGTAGCCGTTGTATTTTGGGAGGTTTGTTGCATGG

AACCTTGTAGCACCGTATGAGGGGCAATAAAAACCTTAAAAAAACTGTTGTTATAACAGGCCTCGACTTCAGATTTTTCT

TAATTTCTATGTTCTTCGAATTATGTGTTTTGAAAAAAAAACTTGATCATTATATATATGTATTCTGCATGATTTTTCGT

TTCGTTATATATATATGTATTCTGCATGATTTTTTCGTTTATGTTTTATAAATCTTACTTAATTTTATATACTTATAGCA

CACAAGTAACACATGGTCTTGTCAATATATCTAACAAATATGAATTTGAATCCCGTTTGAAAATTGATTTGATTATTCAG

CCTTTAAAAAAAATGATTCAATGAATTAAAACCAGCAAAAAATATAGTTCAACATGAAATTAGGCGAGTAGGAGAATCTT

CTTTTTGAACTGAAGCCTTCTTTTCAGACTTGGATTTCTGGACAACAGACACTCATTGATCAGATTCAGCAGGTGGAAAA

GGGAGCTTCACCATCCTAGGTTTCTCTGGAAAGTTAGGCATCTTCCCAAACATTGCTAGTTGATTTAGTGTAGGTCTTCT

GCTGCACCCAAGTGAAAGAAGCTGACCCTTGATCGGGAAAGCGACTACTTAATGAAAGACTCAAACCGTCGGCAGAAGGT

ACAAATCAACCCCTCATTTAGGGGCTCTAGACCAAATAAGCCCCTAAAGAAAAATGAGGCCTGTTTTGCTAAACTACAAG

ATCAGCCATAGACATTAGGTTATTTTAAAAGTCTGTTATAAAATAGCCGTTAGTTAGTTAGGCACTTTCTTGATCTGTTT

ATAACTGCTACGTTGCTCCAGCTTCTATATATACATCTGCAGTATTTACATCTAAAGGAATCAGATTTCAATGAATTTAG

AATGAGAAAATATTATTTTTCATTGAAGTGTTTTAGATGCACATACTGCAGATGTATATATAGAAGCTGGAGCAACGTAG

CAGTTATAAACAGATCAAGAAAGTGCCTTACTAACTAACAACTATTTTATAACAGACTTTTAAAATAACCTAACGGCTAT

GGCTGATCTTGTAGTTTAGCGCGTTTCGAACCTTAAAGTTAGAAAAAATGCAAAAACGTGGCCCAGAATGAACGGTGTCA

AATTTCGCCGTTACTGAGTGTTGACGTGGCTTACTCTTTGACATATGTACAGTCTGCCGTGCTTTCCCTCTTCTTCTCTC

TCTCTGGAAACGCCTCACCCTTTTTCCACCACTTTTGCACTCCCACAAACAACTTATAAACTCCCGTAAAACCCTCTTAT

TTACAGCCACCAATTAACATAATCCCTCTAAATTTTTATTTTATTCTTTTTTTAATCTTTTTAATGAGTTATCAAAACGG

TAGCATTTTGAAGTAACAGAACAAGCAATAAACGATAGCAATAAGAATAATTATATAGCTTCTTTTGACAATTCTAGTTT

CACAGCTTGCTATAAGTGATTAGAGTTTTACTTCTGCAGGGAAGGCAACTGTGATAACAATATCTTGTGGCTGGTGCT

> CbuCOMT 18

AAACAAACATAAATTATATATTAAGATAGAAAATTAATTAAATAGACCATAAACTATATAGTTTTATATATGGGTTATAT

AGGAATTTTATAACCATAATACATAGCTCATTTATAAATTATTAATTATGATCACTAATCATTACGATTAGTAAGTTTTC

ATCCTATACATAGATAATTAATTATGAAATCGAATCACACATTATTACTATTTAAATAAATGACGTTGTGGCCGCGGTCC

TTTGGGTGTAGCGGCACCGTCTCCTCTTTCCACAAGAGAGGTACAAGTTCGATTTTCACCAAGTGCGTGTAGGCTTTTTT

CTACCATATGGGTAGCTTTGAATTCTACTGTTGAGTAATTTGTTTATACTTATTAATGTATTTGATATTTGAATGTATTT

AAATATTTATGTTAATGTATTGATAATATATCGATTGAATGTAATAATTTTAAAAATAAATGAATAAATGACGCCGTGTA

AAAGTGAACAGGTTGGTAGTAAGGTCTTGTGGCTACAAACAAATAAGTCGTCAATTCGAAATAATGTCATCACATTATAG

TTACTCTTTTGAAAAACATAGAAACCAATCATATTTTCCAAATATTCTGTTGAGAAATCAAATCCAAATTTATTTCCAAT

AACATACGTTCAACACTAATTTTATAGAGTCAAAACAAATAAATAAATATATATATATATATATATATATCATTTACACA

CCAAAAGTTGATAAATATTTGAACTCTAAAATTAAATATAAACAAAATGCTCTTAATTAGTATTTAATTAATTATTAAAG

TTCTCTTCTGCCAAATATATTATTAATTTGAATGGTAACTAAAATTAGTTCTAGGTTACGTATGTATAAAATAATTAATA

TATATTTATTTCTTAGATATTAAAGTAATAATTAGAATATAATTAATATATTTAACGTGTGTATATATATAAACGCACAC

AATTTAATATCAAGAGATAAATTAGTGACCAAATTATGCTCTATTATTGGGTTATTGTTGGTTATTTCAATGGGTCAATG

TTAAGAGGTATTTGAAGTATAATACATGAAAACGCATTATTAAAATAATTATTTGAGGTCTTAAGGAGTAAAGGTCTCTT

ACTTGTAGATAATTTGGGAATATAAAGCTGAAAAAAAAAATTATTATTGAAAGTAAAAAAAAAAAAATTCATCACTAGAA

GGACGACGGGACAAGAATTATGTATGAATTATTAATTACTGCCATTAATCCTTAATAAAAAGTCAAAAAATAGTACTCCA

TAAGTCTAAAGAAAATAAATATTTCATGTTGTCTCTTTAATAGTAAGCTCTTATATATGCTGCGAACAGATAAATTGCGA

GTCCAAATCTTTTATTATTAATATTTGAATAAATGATGCATAAAAAAGGTGGGCAGCTTGAGAGTACCGTAGCATATATA

TTATTAATATTTGAATAAATGATGCATAAAAAAGATTGGCAGCTTGATGTCTGCGAACAGATAAACTACAAATTTAAATC

ACATATTATCAATATTTGAATACTCCTACAATAAAATGTTTTTATCACATAAGAAGTACTCTTTTGGAAAACATTAAACC

AATCATATTTTCAAAATTTTCTTTTGAGAGATCAAATAAAATATATTTCCATGCAATATCATAAAGTTCAACACTAATTC

TATAGTATCAATCCAAATATATATTTCATTTTCACACCAAAAGTTGATAAAAAGTACTTGAACTAAAAGTATTGGGAATA

TTATAAAATAATCTTGGAATATAGACAAACAAGCTCATTATATTATTTGACATAATTATTGAAGACAACAAACCAACTAA

TCAATTAAGTATCTATTAATAAATAGTAACATTAATATTTTTAGTTGATGAACACGACATTTTATCATATCATTTAGATT

CGAAAAAGACGATTATAATTTAAAGTTATTTGATAGATTAAAATACCCTTTAAGTTATAGACAATCGACGTTCGAATCTG

GACGAAATATACGCAAATATTTCATCCAAAATAAAATGATATATAAGCATTTTTCTGAAAAATTATATAATTAATTGAGT

CGAGAATAAGTATAATAAATATAAGAATAAGTCATAATTCCAATGGCACAGCGTAATTATCCCTATCGCTACGGGGAATC

TCCAACC

> CbuCOMT 17

TACAGCACTTAAGCTATGACTTATTGGGTTGATAGGCTATTCCCTTTGTTTTTATTCAATCATATTAATATTTTAAAGTA

TAATTATTGTAATAATTCTTATTAATTATAATTCCTTTTATAATTATTTTTTATGTCCTTAGGGACGCAATGGGCAAATT

AACTATGATACTTTAGATCTGTTCCATATTTTTAACTTTTTTTTTCTTAATTTAATGATTATAAGGGTTGAAGACTCCAT

ACATCAATATTTTATTTAAAATTGATTTGATTAAATGACACATAAAAAGGTGTACAATTTAGGTAGTAAGGTCACAGGCA

TTTTGCAATTTCGAATCACATATTATCCATTTTTGAAAGTTTATATAATTAATTGAGTAAAACAAAAAACTAAAACTAAA

GTGTAAAAAGAATCATACTTCATATGACAATTCGTAATTATCCCTAACAAATATTTTATATCAGCCATTTTTCCATATAC

AGAATATTAAAGTGTAAATTGCACCAAACTATTCTGTGAAATCGCAAATTAGCACATAAATTCTCGTGTAATTAAAAATA

GTCTCAAAAACCTCGTGTTTCATGAAAAATTGCACACTTGCCCCAAGCTCCACTTTAGGTTAAAAATGCCCTAAAATGAC

GTAATTACCCTCGTACTCAAAATAGGAAAAAAATGAAAAAACCTGAAACGAAGAAAAAAAAAACCTGAAAATTGAAGGAG

GGAAAAAAGGAGGAAGGTGGGATGGACGAGGAAAGTTGCAATGGCGATGGAAGAAGGCGGCGGGGGTGGAGGAAGAAGAT

AAAAAAGAAAAGAAAAATGAAGGAGAAGGTGGCGGCACAAGGTTTTTGGGACTATTTTTAATTACACAGGGGTTTATGTG

CTAATTTACGATTTTATGGGGAGGTTTTCATGCAATTTACTCACTCTTAGATAATTATTAATAGAAATGTTTCTCCGCAT

ACTGAAAAGGATTTCTTCAGCCACTTTAAAAATACCATTCCCTTTTATTTTCATAATTAAGAAATTAGTATATGATACCA

TATAAAATATGAAGATCCCCACCATCACTTATAATATTCAAAGCTTATAATATTCATGAAAAAGAAAAAAAAAATTCAAA

TTAAGCTTATAATAAGCTCCACGTACCAATTAATTAATTCCAGCTTATAAGGTCATAATCTCCAAAGTCCAAACACCACT

TTTACTAGCTTAATTCAATGGATATATAGTTATAATCTCGATGAAGAAGCTTGCTTATTTGCCTTGCAGCTGGCAACCGG

TTCAGTCCTTCCAATGGCACTTAAAACAGCCATAGAACTCGATCTGCTGGAGCTTATTAAAAAAGCTGGTCCAGAAGCTT

CTGCTTCTGCTTCTGAACTTGCTGCTCAGCTTCCAACAACTAACCCTGATCATGCAGCCGATACGATAGATAGAATTCTC

CGGCTGCTAGCTTTGCATTCCGTTCTCGTTTGCGGCATGAAACCGCTCCTCGACGGCGGCGTTGAGCGGCGCTACTCCCT

TGCGCCGGTGTGTAAGTTCTTAACTAGAAATGAGGATGGAGCTTCTGTGGGCCCTACTTGTCCCTGATGCAAGATAGGGT

CTTGATGGAACCCAGGTAAGTGATCTGTTTAGCTATTAATTAATTGATTTTTTTATTGGTTTTCACACACAGGTGTGTGT

TCATATGAATTCGAAATTGATTCTTTTTTTAGATTTTTTTTCCTTAAAAAATAAATAAAAATAGGGTAGAGGGTAGATTA

ATAAGTTGACTTGAAGAGTTGATACAATTGGAAGATTTTCGATCATCAACAATCCTGTAATTAATATATATATATATATA

CTCAAGTCAATGATCATGATAATAATGCAGTTGTCTTGAAATTGATTAGTGATTAAATATTGTGTCAGTGACCTTAATTG

AGCTAATTAAGTCACTCAATTTCTTTCTATTAACGAGTAGTATTTCAAATTATTTAGTTCGCTTGAAGATATTTAGTTCA

AATTATTAAGAGCACCTCCACCCCGGTTCTATTTTTTTGTCACATCAACAAAATTTCTAGTAAGCCATAAAAAATACAAA

AATAATTTAATATAAAATTTACACTTAAAATTACTATAATTTTTGAACTCTTTATATTAATATTGTGTAATTCAA

> CbuCOMT 16

TTTCCTAATGAAAAAAGACCCTCCAAAAAGTAACTATGCTGCTTCAATTACGACAAGCTCACGGTATTCTTTAGTAAAAG

GTGAAAGAATAGTTCGCTATGTGCTTATTGCACAGCTGCGTGCTTCTCCTGAATCTTTACCCATCCAATACTGTAATAGC

CTCACCACACTTTCACAACACCTACTACCGATGTGGGAAACTTGTCCCCTTATAACATATATCCATTTGCACTTATATAT

GTTTGCTTGAAAAATTAGCAATGACTGAATACCATGATCTGAAATGCATGTTTCGTGTTGGAAAGGGGTTTGTCTGTCTG

ATAATTCATAATCTTAAAAGAAAGAAACATTAGAAAAATTTGTCAAAGAGATATTGGTTTTGAATATGAGACAACTTCTA

AAAAAAGAAATACGCTCTCCTCTATTTCAAATCTCAATCACCTTGGATAAGTGTATACTTTTAAGTACTGGTTTGTTGGA

ATAGTCGCGGATGCCGAAACGGAGTATACTGTTCTATTCTATTGCAACGTCAAAATTATCGGGGCCGTTTCATATATCTT

TTAGTATTGAATTCTTTCTTAAGAAATTAAAATTCTAAAAAGATATTAAAACATATAAAAATTATCATTTCAATTCACTG

CGATCGTCCAGTAAATATATAGGACTTATGTATTAGACGATTTATTGATTTGGTACATTAGTTCACACAAAGACCGTTCC

ATATTGAAACATCAGAATGAAGAAGGTCGTTCCCATTATGTTCCGGCGGACCATGTTTAAGAACCAGTTTGCAATTCACA

ACAATCTGTCTGTGTATTAAATTTGTTTTGAGATACACCAACTCATAATAGATTATTGAACTTTATGTTCTTGTAGCATT

TGCTGGAGAAGAGCCAATAATTAAGCTATGCAAAATTTGAAAGTTTACGTATGTTATATTTCAAGTTAATTATGTACTTC

CACAAACAAAGTTCAGTATATTGAGGCAAAATTAAACAAATTCTACACTTTAATATCCCCTATTTTGTGCACTTCAAATG

AAAAGATTAAGGATCTTGATAACCAGATATACTCAAAACTCTGCAACTGTAAGGTATAATAAAATTTTTTGGTCCTTTCT

ACGGTGGGACTTGGTATTAATTCTTTCGAATTATCTTTTATTTTCACTATGCGTGGTGGTGAACTAAATACATAGAAAGA

CAGATTAGGGATAATTAGAGTAGGAAATTATGTAATTAGGCAGTTCTATATATTTGTGATATCTTTGAAGCCTTTCCAAT

CTTACTTATGAATCACAATGAACAATTAATCACTTGTCTGATCTTGACAGTGGCTAAGTAATTGTCCAAATTAAGATGGA

TTTGTCTGATGCTGCATTGGTTAATTGTCTGGATTATGAAGAAAAATCATAGGTATTGGTTCCGTAAGAAAACGTACGCG

GTACGATTATGAATCAAGATTTAGCAGTTTGTAATGTGATTAACGTGTATTGGTGGTGAGTCGCCATGCACTAATAAATT

AACTTCACAGTGTAGGCCGTCTTGTCTCTTTCCTTACCATATTTATTATAATATAATGTTTGAGATAATCAGTAGAAGTT

ATAAGGAGGATTGCTGTTGCTGCTGGATGTATACATAAGAACAAATGTTGTAATTAAAAGGTTCAAAACCTATCAAATAT

CAGACGAGATCTTCTATTCCAGTTTCGTGTTCCACTTCCCTATTCCACTAATAATACATTGTCATGTGTATAGTGAAACA

ATAAAAATTAAAAATAGTAATTGCAAAAAATAAAAAGAAATTATTTCACCGACAATGTATTATTGGTGGAACAGGAAGTG

GAACACGAAACTGGAACAAGAGATCCCGTCTGATCAAATTATATAGTGTGCTGCTGACATGGTTTATCTTGTAGTATACT

GAGATCAAGCCACGTGTCTTGTCGGACAGTCTAAAATGTATATGTGTTGAGTTGGGACCTTCCCCACGTGAAATAATAGA

TATCTCCCGGGGCATTTTTGTCAAATCGTCCCTTTAGCCCCTATCGGATACGGATTAATTAATAGCGTGCTTCTGTTCCT

TTTGAAATTAACTAAGGGAGCAACTATATAAATGTGTGCAGCTGCTTGATTTTCTTGCACATCACATCAACTGAAACAGA

GAG

> CbuCOMT 15

TAGGGGTACAATCAACTAATTTTTTTCAAACACAGGAGAATAAGTGCCAATTATAAATAATATAAGAGTAAAATATGCAA

TTTACCCTTAATTTAAAACTCGGATTCGAACATGTAAAAAATCGGATTACTTAATTACACACCCGATTCGACCATTTTCA

ATTACAAATATTTATAACTAAATTTAATTAATCAATTAAAAATCTTAAAATTCAGATCCCGAACATTACCTTGAAAACGG

GTCAAGTAATCTAACGGGTAACCCGTTTGTCTATTGCAGTGGGTTTGCCACAATTGGAGCGATTCAAGTTGCGAAAAGCT

GTTGAAGAATTGCTACGAAGCGCTGCCCGAAAATGGAAAAGTGATTGTTGCCGATGCTATTCTGGCCGAGGACCCAAATA

GCGGGCAGAGTTCTTTTTGGGCAACCCGAATTGATGTGATTATGTTAGCTTATAATCCAGGCGGAAAAGAGCGGTCAGAA

AGGGAATTTGAGGCCTTGGCCAAAAAAGCCGGATTCAAACACCTTATCAAAGTTTGCAGTGCTTATGCTGATTGGGTTAT

GGAATTTCATAAATGATGGGAAAAGTTTGTTGGCTTATTGGGAAGGTTTCACAATTATATTTTGCTTTTTTTATTTGTTT

TTTGGGACGAATTTAGGTTCTAGAAATAAATTATGTCGAGTCTAATAAATTAGGATCTCGTGAATGGTGCGGATCAATGC

TAATATTAGGTCGAGTCGGTCTCTATAATCTCTTAGTATGAATAACTTCCATAGGCCCCTTGTCTAGCCTATCAAACGGG

CTCTAAACCTCAACGGATGTTATATAGGTTAAATTAATTGAATAATAATAAAGATAAATATCAATGAAAAGGAGGCTAAT

GTTTCACTGATAGAAAATAATGTAAAGATATACAAAATTTGGATGATAAACTAAACATAAAAGGGCTATATATAGCTTAG

AGTAATAATCTAAACACAGAAGAACAAGTAAATAGTAAAAGGCAAATATACCCTTAATAAATAAATAACATAAAATCTTC

ACATCCTCCCTCAAACTCATGATGTGGCAGCTACAAGCATCAAGAGTTTGCCAACTAGAAAACGAAAACGAAAAATAGAG

AGCAATTTGATGAAAAAATTGCAATTTGTAAAGAAGAAGGAACAAATGACAACATAATAGTGCCGTGCTTGAAGTGATGA

CAATCGATCTCTATATACTTAGTTTGTTCATGAAAGATTGAGTTATGAGCAATTTTACATTCAATCATCACATCAACATC

AATATATAGGAATACAAGACCGCATGCTTTTCTTTTTTAAGAAACGTTATTTAGATATTATATTCAATTATTACATCAAT

ATCAATACATCAATACATTTTTAAATATTAAATCATATACCAATAAGACAATAAACTAGAATGTGGTAGAGCAAAGACTA

TTCTAAAAACTCGCACGATTCTTATATTGCACCTCTTCTATGAAAATGAGAGGCGGTGTCACTTCATCTAAAGAACGGTG

AACAAGACTGCATGCTTCTAGTAATCTAGTTTGGTCAAATTAGGTTAAATCTATATTGTAAGGGCGCAATTTTAGTGTAG

CAATCTAGTTTAAAAATTCTTGCATCTTTGGTATTTGAAATCTTAGTGATATTCTCCTTAGTGTTTTTTTTGTGACAAGA

GATACTCAGTCTATTTGAAAGTTTAAAATATTTCATCAGAAAATGAAGCGATTTGATAAAATGCAATATTTGTAAAAGAT

TGTATAAGGGTAAAATGCAATTTACCCTTTATGATAAGCAAAAGTACGTAGTGGTTTTTTTTTTTTTTTTCCCACACGGG

ATAAACTGCTACTTTCGTTTATTACAGGAGGTAAATTGCATTTTACCCGTTTGTATAATCATTTGAATAATAAAAAAAAT

AAAAAAATATATAAAAATAGTTCATAATTCCAATAGTAAAGGGCAATTATCTTATCCCCCTAGCTACGGTGAACCAGCAA

CCCCATACCCACCACTACTTATATATTCTCCTCCTTTTTCACTTGCAAATCAAAGCTTATAATAAGCCCCAACAGCCATC

CAATTCCAGCTTATAAGCTCATCATCTCCAAAACACCACTTCTACTTCA

> CbuCOMT 14

ATTAAGTTTTTCGACAATTGAACTGACAACAAGGTATCGTTATAATTTTCCGTTTTGAAATATTATATATTCTAATGTGG

ATATCTTCTTCATTGTGTACAAGGGAAAATGTGGGTGATTATTGTAGTGAAATCCGACAACTTGGGCTAAGATTGCAAGA

AGCAATATCGGAATGTTTGGGCCTGGACAAAGATTACATAAATAGAAGTTTGGGCCCACAGGGCCAACATATGGCCATTA

ATTATTATCCACCTTGTCCGGAGCCCGAATTGACATATGGGCTTCCGGCCCATACGGATCCAAATACCCTCACTATTCTT

CTTCAAGATGCTAAAGTGACAGGGCTGCAGTTGCTCAAGGATGGGAAATGGTTGGCTGTGAAACCCTACCCAAATGCATT

AGTTGTTAATATTGGTGATCAATTGCAGGTAAGGTTTCTTATATTTAAATTAATTATAAGTTGTCGGATTATCGAATATC

ATTTTTAGGGACGACAAGGGGTTCTACTAGAAGTTGAATCTATCTCATTTGGGATGAGTTTTGAAATTACAAACGAGTTT

TAGATGAGTCATAATTAGTTTTAGTATTGCATCTGGCCACTAATTGACACGTTACAACATTGTATATGTATACTTTTATT

TATTATATATAGACTTATTCGATGCGTTTTATTTATTTATACTAGCATCTTGTGCATTATATGCGTGTATATATTTAATA

ATAAAAGTATAATTCACATATATAAATATATGCATGTATAATTCTATGAATATAAAGTGATAGTAGTGGGTTGTGAATCA

CGATAATTACTAAAAACAATTTATACAACCATATGGAAACACATTGAGCGTTTTAGGAAGATGAAATGAAAATATATGGA

AAAAGGGTTGACGTTCTATTAATAGTTATAGATACAAAAGTACATACGATATACATAAAATATAAATATAATTTTTTATA

AATTAAAGTGATAGCACTAGGGCGCTGATAAAGTGATGGTGTTACTATTTCTTTACAATTTTTTTTTTTTTTTCTAATAC

TTTTAAAACTATGAATGGGATATAACACGTACAACCCACTGTATCTTGAATTTGGGACAAGGCGGGTCCTAAATCCAAAA

ATCCTAAATTACGGGTCAAGTCTCATATTTTGACAGATTCATCAGCTACTGTCCCTCTACAATCTATCGATTTCTAATTC

ACTTAAATATATTTAGAGGAATGACATGACATGATCTGCAGTTATCTAACCTTGATTTTATTATTTTATGCAGGCATTAA

GCAATGGAAAATACAAAAGTACTTGGCATCGAGCCGTCGTGAATGCTGACAAAGCAAGAATTTCAGTAGCTTCCTTCTTG

TGCCCTTCCAAAACTGCAGTAATCAAAGCTCCAAAGGAACTCATTGAAGATGACTCAAAAGCCATGTACAAAGATTACAC

ATATGCAGAGTACTATGAAAAGTTCTGGAGCAGGAACCTTGACCAAGGGCACTGTTTGGAACTTTTCGAGAATATGTAAG

AAGAAGGCTAACCTCTAAATTAAAGTTCCATCCTTCTACTTTTATGAAATTTGAATTCAAGAATAAGTTACCTTTAGGAC

AAGAAATAATTGTGTAATGGTAATTCCTTAAGGTCCAAACTATATGTTTTTAGGGTCACTTTTGCATTTGGATTAGTTAA

TTTGCTCCAGTTATTACATTGATATGTATAGTGGAATGAGTTTGTTGTCCTAATGAAAAGTTATTTAAGAGACACATTTG

CAATTATCTCAAAAATAACTTATGAAAAAGTAAAAAAAAAATAAAAAAAAATAAAACTCGACAACTGAACTTGAAGGACA

ATCTACTTGCAAAATGGATTAGGGAAACTGGCCGTTTTGAAATAATAAACCTTTAGTAATTAAGTATAAGAATCTACCTA

CAAGATGAGTTAGAAACGATTTTTTCTCCATAAAAAACTCTTTAATAGTTAAGTGAGTCTTGAAAAACAGAAAAAGAATT

TATTAGCAAGAGTTATGTGCTTATATGGCTCTAAAAACTAATCCATATAAGCTATTAGAACATCATACTCATCCTCGCTT

GCCACTACCAATTTTAGTGGATGGTTGTTCCTAGATATTACAGGTCCTTCGAAATCTTTCCAGTCCTTATCCCATAT

> CbuCOMT 13

CGCATCTGAAACTTCCTCTCCTCCCGTGGTCTCCCAAACACCGTCTGAGATTTTGGATCCACCTCTCCGACAGTCCACAC

GTATTCGTAAGTCTACAAAGTTACCAGATTTTTCTTATTCTTGTTACTCTTCATCTTTCACTTCATTTTTGACATCTATT

CATTCTCTTTCTGAGCCCTCTTCATATCAAGAGGCAATTCTTGATCCTCTTTGGCAGCAAGCTATGGATGAGGAACTTGC

TGCTTTGAATAAGACAAATACTTGGGATCTGGTTCCTCGACCTTCTGGTAAGACTGTGATTGGTTGTCGCTGGGTATATA

AGATCAAAACTAAGTCTGATGGGTCTGTTGAGCGATACAAAGCTAGGTTGGTTGCAAAAGGATATTCTCAACAGTATGGT

ATGGACTATGAGGAAACCTTTGCTCCCGTTGCAAAGATGACTACTATTCGAACTCTGATTGCAGTGGCTTCAATTCGTAA

GTGGAATATATCTCAATTAGATGTTAAAAATGCTTTCTTAAACGGGGACCTTCAAGAAGAAGTTTATATGATGCCTCCTC

CTGGTGTTTTCTCATGATTCTGGGTATGTTTGCAAGTTTAAGAAAATTTTGTATGGCCTTAAACAAGCACCCCAAGTTTG

GTTTGAGAAGTTTTCTGAAGTGATTTCATCCCTTGGATTCACTTCTAGTAGTCATGATTCTGCTCTCTTTGTTAAGTGTA

CTAGTGCAGGACGTATCATTCTTTCTTTATATGTAGATGACATAATTATTACTGGTGATGATATTAATGGTATTACAACT

TTGAAAACAGAATTAGCTAAACAATTTGAAATGAAGGACTTGGGTTCTCTACGTTATTTCTTAGGGATTGAGGTAGCTCA

CTCACTTAGAGGTTACCTTCTATCTCAGTCGAAATATGCTACAGATATTCTTGAACGAGCTAAACTTATCGATAATAAGA

CTGTTGATACTCCTATTGAGCTTAATGCAAAATACTCTTCTTCTGATGGTATATCATTGTCAGATCCCACTCTGTATCGT

ACTAAGCATATTGAAATCGACTGTCATCTTGCTCGTCATCACCTCAAGCATGGCATTATTACGTTGCCATTTGTTCCCTC

TTCTTTACAGATTGCAGATTTCTTCACCAAGTTGCTCTCTACTTCTCGTTTTCGTTTTCTAGTTGGCAAACTCTCGATGC

TTGCAGACGCCGCATCATGAGTTTGAGGGGGGATGTGAAGATTTAATATATTATTTATTTATTTAGGGTAGATTTGTCTT

TTACTATTTCTTAGTCCTTCTGTGTTTAGGTTATTATCCTAGACTATATATAGTTCTTTTGTGTTTAGTTTATCATTCAA

GTTTTGTATCTCTTTACATTATTCGTTATCAATGAAATCCTAGCCTCCTTTTGACATAGATGTTTTATCTTTTTATCATT

CAAATAGTTTAACAATTAGAACCGTATTTTCCACCTAAAATATCCCACCCTTCCCGTTACATAATTATCCTCCCATCGAC

AACTAAAAGTATCAAATAAATGAAATAATTATCATGTACTGAATGTCAGATATGAAAGTTTGTTGGAAATTATTTAATGC

CTTAGTTGGAAGTATGGCATATATGTGCATAACAGCATGAGTTGTCCCTTCAATAATTTCAAGATCCTTCAATAGATAAT

AAAGCAGCTTTCGATCGCCCTCAAAAATGAAAATGGCATCTGAAATAAATAATTTGAGTGACCTTGTAAGCCTTTTAAAT

CTTTGTATGTAAATATTTGAGTCTCATACTTTATAAGATGATATAAAGTATTTAAAATAATAATAAGAAGAAGAAATTCT

CTACTTATTTTAAATTTAAGATGCTAACTCATTTATATTTAACAATTTTAATTACAAAATTATCATAACTAATATCTTAT

AAGTTTCAACATCTTATTTTCTCCAAACAATATCTCAATAACTAATTATTTTTTAAAATAAAATCTAATAACTTTACATC

ACAAATCAAGTCTGCATGACTTATCTGCATTAATTAATTAGGAGAATAGAAAAGCAACCTTTGCATTGATATATAATATT

> CbuCOMT 12

TTTAAAATAAAATCTAATATCTTATAAATTTTAAAAATGTGTTACAAGCTATTTTAAATAGACCAAGCCAAACACCCTCT

AATCCTCTATATCACAAATCAAGTCTGCATGACTTATCTGCATTAATTAACAAGGAGAATAGAAAAGCAACTTTTGCATT

GATATATAATATATATTATTCAACATACATCACTGCTAATCGTAGAACACACATTACAAGATTCTAATTAAAATCATAAA

TCAAGTTTCACAATACTCATAGATAGGCCTCAATAACAGATATAATTTTACCTTCAATAAGTTTTACAGTAAACTTGGTG

AAGCCAGCTGCATAAACCACATATTCTCATTTTTTAATAGTCCTTTATTTTCTTTTCTTTGTGTGAGCCAGCATAACCAT

GTCCAAATCAGACGAACATCAGTAAACTTGTCCTCTTCTCCTTCTTCAACAATTGCCCCCGCGATGATCACTTTTCCGGT

GTCCTTGGAAATAGCTTCACGACATTTTTTCAATATCTGAATACATTCATTGTCACTCCAATCATGTAACACCCACTGCA

TAACTAGTCTCCAATCATTTTGAACGAAAAAACAACTCAAAAATAGATCAGTTCAGGTGAAAATAAAGCATACATAAACC

TGTAAATGTGTGTTTTGATTTAAGCAACAACAGTTATTACAAGTTTTCAACGTGGGAATGCTATACAAACTGGTTCATTA

ATACCAAATTTCATAGTCTAATCTAAATTAAATAATGTGGAAATGACCATTGAAAAAGGTGATGCACATCGTTTTCGCTA

CTTAAGGAAGTGTTTTTTATTGACTTTAACCATACAGCTAGTAGAATTGTGTGCATTATATATATATATAAACTACTTTA

GAGCTGCCTAAATCTACAGTAGAGCTGCCTAAATTTATACTAGAACTGCATAAATTTATAGTAGAGCTGCCTAAATTTAT

ATTAGAGCTGCATAAATTCAAACCAAACACATTGCATTAACAAGGGGAAGATAAGGGAAGGTTTGGAAAGGATCTGAATC

CCATAGACTGCAAGTGGGAGCAAGAGAGATTTTAATTTATACTTTGGTGCTCTTCGAACTAGTTTTTAGCAAAAAAACAA

GTGGGGTGAAAAATGTAATTTTAAATTATTTTTATATTTTAATTATAATTATAAAAAATATTTTAGAGAAGATAATTATA

TATTTTTTATATGTAATGATGATTAATTTTTTATTTTATTTTATTTTTTAGTAATACGTACTTTTAATATATTGTCATAC

ACCCCCTATATAAGTCCACGTCAAAATGAAGATGAAAAGAAAA

> CbuCOMT 11

TCTTGATGTTTATTTTGATTGCAGTGTGTTGTATTATGGATTGTGATAACTATATTTCAAGGGTTTTGTAGAGATTATAA

TGGACATGTTTTAATCTATTAAAACTATTTTAGTTGTAAGAGGTATTTGTGATTTAAAAAATTAAGTTTGTGTGTGTGTA

TTAAGGTGCTTAATATTAGATGAGATTTTTTATTTTATTTTTGTATTTCACCAATATTTTATTTTTTTGTGTTTCACCAT

TGTAAAAATTGCAAAGACAATAGTAGATTAACAACATCAATTTAAAAAATATTTCATAAATAACAATACATAATAATAAC

AAGTAACATAACATCCTAATCACGACCACCACCACCACCACCATGTTTAGGAGGAGAATCGTCCAGATCGTCTGCCTCCA

TTTTCGCATCCGCCTCCTGCTCATTATGTTCCTCAATAGCATTATCAACATTGTCGTCCGCACCATCAGACGCAGTGTCG

CAATAATGAGATGAAGATGACCACTGATCACCTACGGGTCCAGAAGTAAATGGTCATGCTATATAGAGACTTACAGATAG

ATGATGATGTTTGTAAGTCATCTATATAGCAGGGCCCGCTACAAACATCATCATCTATCTGTAAGTCTCTATATAGCATG

ACCATTTACTTCTGGACCCGTAGGTGATCAGTGGTCATCTTCGTCTCATTATTATGAGGATATACCCATTATTACGGCAC

TGCGTTTGATGATGTGGACGACAATGTTGATGATGCTATTGAGGAACGTGATGAGCAGGAGGCGGATGCGGAAATGGAGG

CAGATGATCTGGACGATTTTCCTCCTAGACATGGTGGTGGTGGTCGTGATTAGGATGTTATGTTAGTTGTTAGTAGTTAG

TATTATGTATTGTTATTTATGAAATATTTTTTAAATTGACGTTGTTCATCTACTATTGTCTTTGCGATTTTTACAATGGT

GAAACACAAAAAAAAAAATGAAATATTGGTGAAATAAAAAAATAAAATAGAAAATCTCGTTTAATATTATGCACCTTAAT

ACACACACACACACAAACTCAATTTTTTAAATAACAAATACCCCTTACAACTAAAATAGTTTTAATAGATAAAACATGTC

CATTATAATTTCTATAAAACCCTTGAAATATAGCTATCACAATACATAATACAACACATTGCAATCAAAATAAACATCAA

GAAATGTCACAAAAATCCAAAATTCCCACGTTTACTATGGTTGAATATTGATAATTATTAACATAAAGGTTCAAAATCCC

AATAACAATTCAACATAATTTCTTCCTTCATAAAAACAACTTAACCATCAACATGAAATTATCGGATAAAAAGCTAAAGT

TTGCTTGTATTTTGCAACTTCGACAAAACTATATACATGCCAAAAAGATTCAACACGTACATCTCACGAAAGGCTTCTCT

TACGCTTGCAATTCCAATTCTAAAATGGGTGCTAGACTACATCAAGCGCCCAAATTGGTCGCCCGAGGGAGAGGGGGGTT

TGGGCGCCCAAGGGGTGTGCCAGACGCCCATTTTGGACGCCTAGGGGGTGTTTTGGGCGCCCAAACCCATTTTTTTCGAT

TTTTTTCGATTTTTTTCAATCGATTTTACGATGAAAATATAATCAAAACGATGCTAGAACTTATATTTCGATAGTTGATG

AATTATTTGAGCAAAATAATTAAAACGATGCTAGAACTTATATTTTGATAGTTGATGAATTATTTGAGTGAATTTGGAAG

ATATTTGATGAGAATTTGATGAAATTAGTAGAGAGAAATTAGATTGGGGGGTGGGGTGGGGGTTCGAATTAGAATTTTTT

TTGTGAATATTTGATACGAAGTAAAAACGGTCTGGCATTTAGAACCCCTCATTTTCAAATGGATAAAATAAGGGAAGACC

AAAATGGCAATTCGATTGACATGACATGTGCTCCACGTGTCCCAATTCTATTGGATGTTAAGATCATGCTGTTTTAACTT

TCCCACATGGATAACTAGTAGAGCGTCAGTGCATGATTAATTATATATATCCTCTATGTGAAGTGTCACATGCATGCTCT

TCTTTCTCAAAACTTATAGAGTAACCCAATTTACTGATAAGCTCAATAAAGTTGATAAAAAAAAAAAAA

> CbuCOMT 10

ATATCCTAATTTAATCTTCAGTCCCGTATCGTACGGCACCTATAATTTAAGGAAATTTATTTACTTTGTCAAATATGATT

CTTTCGAATAGTGACAAGCGGCTGACAGAATAGTGGAAGTAGTTAACTCATGATAGGACACTTCATTGAATAAAACAAGT

TCTACATTTAACACCCTCATAAAAATTGAAGAAAGTGTAGTCTATTTCCAAGATTTATAAATTCAAGAAAATGGAGCCCT

ATTTTCAAGTTTTTATAAGTTCAAGAGAGTGGTCCCAAGGTTTATACATAAAAAAGAAAAATACATATTTTTTTTAAAGA

AAAAGAACAATATATTTAAGGTAGAGTAAGATAAAAGTACAATATTTTTTAAGAAAGTTATTATTTATCAAATCATTTTT

ATTAAGTGTACATTTTATAACTAAATATTAAAATTAACTCTCACACTCTCATCCCATACTTCCCCCAACATCTCTCTCTC

TCTCTCTCTAATCCCAACCCCTTGATCTTACAAAAAACTCTTCAAATCTACTCTATTTCTCATAAAAAAAAAAAATATAT

GAATTCATCATAATCAGAGTTCAATTCGACTGTTAGTGAAGTAAATTCAACTTTTTTTTTTTTTTAAAAAAAAAAAAGGG

CCCAACACCCATTTTGGGAGTTGGAGTGGCTTGCTAGCTCCTGTTTTGGGCACTAGAGTGGCTGGTTAGTGCCGAGGATA

ATTATAGATGGGAATTGGACAATCTTACGTCTCTTTTCAATAGAGACGTGCTACCTGGAGTGATTGTGATGGATAGGGAG

TTGGGTCTGATAAAGGCATTGGAGCGTGTGTTCCTAGGGACGTACCACCTCATCTGTCTATTCCATATAAATAGAAATGT

CCAGTTAAATGCGATGAAGTTCTTTGGTGGCAATAACTTGCACGGCATTGCATTCTCCTGTGGGAGGTAGAAAAAGTTGG

TGGAGTCTTGATCGGAGGATGAATTTAAGGACAATTACAATCAAATATTATAGCAGAATAAGGGTAATCCTCATTTAATA

ACCTACACTTAGGAGACCTGGCTCATGTACTCGGAGAGATTTGTACGTGTCTTGACCAATAAAGTTTTGCACTTTGGCAA

ATGCAACACGATGACGAATAGATATTTAATTTTTGCAAATTTTCTTTATAGATACTCTTTATTTATTTAATTCTTTAATG

TCGGGCTTCCTTACATTTTATTTAGTTATCAGGATGGAATATGTGGGTGGGTTGTGGATGGTCGTGTTATAGTGGAGCAT

GTAGTAGGCATATAGTAAAAACTATACTATTTATTAAAATAAATCAAACAAAAAACAAAAAAAAAACAGTATTTTAAAAA

AACACATGAGATCATGTAAAATAGGATCTCGAAAGGTTAGATATATAGTACACTCATGTATATGCATAACATTAGCAAAA

TATAATTTATGTATACTAATATATAATTCACACTATATGTATTTGGTAATTAAAATATATAATTAATATATTCAATATAT

ATATATATATTTTTGTTGTTTGTATGGTGAACGGTAACGTTCAAAAATGACAAGATTTGACATGTTGCAAAAGCCATGCA

ATTCAAAATAATCAATGACTTGTGCCAACTACTATAAGAAAAAATGATATTATTAACAAAATATTATGTGATAAAGTCGA

GGTAAGAGTCTTGAAGAAGTGCTAACGCCATTTCTGCAGCTCCGTGATTGCTATTTTTACTTTATTGTTTTTCTTTGTAT

GATTTTAATTCAAATCAACACCTAGATATTTGGATCGTTTAGTCATAATGCATGAAAACACAAACATCAAAATAACTTCT

CAAAATCTCAAGCTTAATATACTATAACTAGGGAGACGTCGACTTGTAATTAATGTTCAGTTAATTTGTTATACTTTCAA

GGAGTCTAATTGTAATTTACAAGAACAGTTATTATAATTTATCAAATTATAAGGGATATTTTTATAATCAAGCCATTATG

AGATAATTATCCCAAGGTAAATAGCTCCTATGATTTGTTAAAGGAACCCTCTTAAGAATGACCATCATAAAACAACAATT

CATTTATTTGTTCTTATGA

> CbuCOMT 9

CTATTATAAACCGTTTTGAATTCCGAATTGGCTTGTTCATTCTTGGTTTCTAAATTCTGAATCGTATGATTTATATTGTT

CTATGCATGCCTTGTTTCATATTTGCAAAACTGTTGTGTTTTTAAAACAAAATTTCCACTTGTGATTATTTGTTCATTTT

AGTTGATAATCTGAATAATCTATTTTAATAGATTACACTCACAACTGTATATACATGTATACCCTTGAAATTTATTGACC

ACTGAAAATTCGAAATTAGTAAAACTATGTCCATAATGAAACTTTAAAATTTTTATTAATATTTTAATTTATCAAAATAT

GTTAATTAATGAAGTTTACTGGCCTTTTACATTAATTCAAAGCATGCACAAATAAATTCATAATATTAATTTTATAATTT

TATTGCTATTAATGGTAAATAGGTAATAATATTGATATATGTAAATATTTAATTGAATAAGTAATATTTATAAATATATT

AATTTTAATACATAAAATTCTTAAGAAGACTAAAACAATGTTGCACATCTATCAAACAATCAATTTAATCTCACACATAA

CACAATATTAATATATTGGGAAAAAGCTTATAATAAAAAGCTAATATAATTGTGCATATATACCTTTCTTGTATAAATTA

ATTACACATTCAACTGACAAAATTTGATAGCTTGCAAAACATGCAATACAAAATGCAAAATAATCAATTATTCGTGCCAA

CTATTATAAGAAAAAATTGATATTATTAAAAAACAATTATGCAATGAAGTCGAGGAAAAAGTCTTGAAAAAAAATCCTAA

TGCCATTTTTGCGGCTCTATGATTTCTATTTTTACTTTATTGTTTTTCTTTGTGCAACACCTAAATGTTGTAAATTTTTT

AATTATGATGACCAGCAATAATTTTTAATCAGATGTCCACAACGAAAGTCACTAAAAAAAAATTAAAAAAAAGTATAATT

TTCATAAGCGATATTTCTGTAATTTAACCATACCTCAAGATATGTAAGGATTATTATCCTAAGGAAAATATCTACTATGG

TTTGTTAAAGGACCCTCTTGAGAATTACCATCATAGAACAACAATTTATTTATTTGTTCTTATGAAAGTGGAAGTCCATC

ACTTTCACTACTAGAGAACATGATAATTGCAATAGAATTTTGTCAATGGACAAATTCTATTACTTATTACAACATTGTTC

TATGGATAGGCTTCAATGACAGATTCAACAGCTTTCATGTGTTTGATAGTGTGTTTGCTGAAGCCTGCGGCATTTAGGAG

ATGTGCCCATTCCTTATAAGTTCTCTCCTTCCCTTTTGTTGTTACTGTCATCATTGTTATATCCATTGCCAAACGAGCAC

TCGTATACTCATCGCCTCCTTCTTCATCTATTACAGCTTCAACAATGATCACTTTCCCTGTGTCTGCTGGAACAGCTTCT

TTGCATTTCTTGAGGATGTCTATGCACAGGTTGTCGCTCCAGTCATGTAATATCCACTGCCATGAAAGGAATTTGAGAAA

TGATATATAAAATTATAAACTATTTTAAAATTCTTGATTCCTGTACATGGGACAAAATTTTGATATCATTATAAGTTGCT

CAAATGAAAGTTCAAAAAGAATCACATGATCGTCCAATAATAATGACTTAGTTATCCATTGTGAACTTTCATATTTAATG

AACATATTAAAAAACACATGAACATGCACACAAATAGGGGTGTTAACGGTTCGAATTGGATCAATTTTGAAGCAAAAATC

TATCCGATTCAAACTATATAATTTTTATTGGATTGGTTTGGTTCAATTTAGTCTTTTAAATAAATACAAACCAATTAAAG

TCAATTTGAAATGGATCAGTTTAATCGATTAGATAATATAACAAATAAAAATACATAATTCTATTAAAAAACTAATTATT

AATTTAAAAGTTTAATTTGGATTGAATCAATTTTCAAAATTCAATCTGCAAACCAATCAAACTTTTCCAAAATTATATCC

AAACTAATTCGAAATGAATCAAGTTTAATCAATTTTTAATTTATTTTAAATTAAATGATCAATTTTGTTCAATTTGCCGT

GGATATGAACACCCCTACACACAAACACGTAC

> CbuCOMT 8

GTGATACTGTATTATTAATGAAATAAGTAAGAAGAATGCGAGATCACATTTGCACTAACTGAGGCTGCATGTGAGTGGTA

AAATTTTCATCAAAAAACTAACTGAGGGACTAACATCGTGTCAAGAATAAATAAAAAGGCCATGCTACTAATTAATTCAC

ATTCAATTTAATTTAAATATAAATATTCAAATTATTTTTCACATCGTGTAAATTATTAAATTTTTAACGGACAACTTATA

ATCTTCCTCAAAAACCTACAAAAATTATTAAATTATTTAAAAATTTATCTGGTCGACCAAGCGATTGAAAAGATATATAT

TAATAGTACATCACATCATGTCAAAATAACGAATTGAGCAATAATTTATGGTTTATTTTGTTTCATATCTTTGGACTTAT

TTTTTATCGATCATGAATAGGTCATGATTACGTATTAACTTTGATTCTTTTTTTTATTTAATATTTAACTAATTTGTGAA

AGTTGTAGGCAAATGTAAAGCTACCCCAATATGTTTTGCATTCATTATAACAATCATAATAAATGCCAAAAACAATTAAT

TTGCACTCCTCTATTCAAAGCATGTCACATTGACATGAAAACACCGTCACATTCACACCAATAAGTTTGTAGTGAAAAAT

CATTTGCATCTTCAGCACGATAATGATACGTAATTTTAATAAAAATTGTGAAAAATTGACTAGGCAATATAATGAATCAG

GCCATGAAATTACAGTAATAAAATAATTTATTCTCAATCACTCACTTGAAAGCACTCTCCAAACCGTACTATATAAGACA

CTGATCTTGAATAGTCATTACCAAACAAGGTTTTTTATTTTAAAAAAAGACAATCATCAAACAAGAAAAAATAGATTACA

TTAGGACAAACTCACTCCATTAAGGATAGGCCTCGATGATTGATTCGATAGCTCTCATATGCTTAATGGTGTATTTGCTA

AAGCCACTTGCATTGAGAAGTCTTATCCATTCTTTAGTAGTCCTTTCCTTTCCATTGATAGTTGCGATCATGATCATCAT

GTCCACCAGCAAACGTGCACCCATGTACTCATCTTCTTCTCCTTCCTCATCAATCACAACTTCAACAATGATCACTTTCC

CTGTCTTTGTCCGTATAGCTTCTTTACATTTCTTGAGGATTTCTATACACATTTCATCGCTCCAATCGTGCAATACTGAC

TATCATACAAAATAAACAAATTTATATATGATATTCTTGTATTAATACTGTAATTTTAGATCGAATAGTTGTACATATAC

AGGTAACTAGCAATGGATAACTCACAATTACTAATAGCACTGTACACTCGTAATAATACATGTACATAAATTATTTGTAC

TCATATTACTAAATGTACACACGTTGAATGACGTTCAAGCAATGAATGCTTGTAACTACATCAATCTTTGAAGACTTTTA

TATGAAGTGGTTTTTTATGCTCTTAATTAATAAAGAACTTGGGTGATTTTCATATATACAATCCTCCTTACAAGTTAAAC

GGGTTAGAATCTTCTATTCCAAAACAGTGTTTTTCTTCTATGGCATTGTACGAATTCATGCCAAATGTCTTAAATATTTC

CTTATAAATACACATAATTAATGTGGATGTCATACTTTTGACATGAATTCATTCGGTGGAATAAGAAATAAACATAAAAT

GGAATAGGCCTCAAGACTAGCTAATATAGTTGTAACTTATGCAAAATGTAACTATTATTAGATGTTTCACATGCAACCCA

CTATTGGTCTATCACCACTTTGCAGTCAACATTAACGATACGAGCATTTTCCGGATGGAATCCCCTATTTTAAAACGGTG

TTACATCTCTTATTCTATATATATTTTAAATACATGTCACGTGTATTATATATTTTCTTATAAAAATATTTAGTAATAAA

TGCAATACGCATGACATGTATTTAAATTTTAAAGACATGAAACAGGAACTGAAACACCATTTTAAAATAAAGAATTTCAT

CCGCATTCATGTAATGTACTATAATTCTAAAAAGGGCAGACACATCGAATATGCAACGAGGGTACCATGAGCATAATTGC

ATCAGCTTTTGGAATGCTTTCAAACATGTCTCCACCAACATGCAGAACCCCGTCAATGGCTGGAGCCTCATGTACCACTT

GAGGAA

> CbuCOMT 7

TCATTTGAGGTTTTTTTTTTTTTTTTTTTTTTCAATTTCTACGATTGAAAGAGGATGCAATGGGACAACTTTCACTATTT

TAATCCTCATAGCAAGGTTTTGAAAACCCGTTCCTAGTGACAATTTACAAATCATTCATCGCATAAGTTAGATTTGAGGG

TAAAATGCAGATATCCCCTAACTATAGGGTCAATTACAATTTTTCCTATATGTTTTTTGAATCAGTCATTACAAAATATT

TGGTTTCACTCCCTAAAACCTAAATCCGGCAAATATAGTGAGAATATTTAATTCAAAGCCCCTCACGTGTCTTCAATATA

AATGCAATGAGAGTGTTTTAATAAGGATGTCTCATCCTGAAAAAACTAGGGTAAATTAGCCCTAATTTTGTTTGATGAGA

CATCATTTATTAAAACACCCTGACTTTACTTATGCTGATGATACATGAAAAACTTTAAGTGGATTCGCCAAATTTCGATT

TTAGGGTGTGAAACTGAGCATTTTGCAATGGTTGTGGATAATCGCACGCTCAAAAAATTAGGAGGTAAAGTGAAAATGAC

CTTATAAGTTACGTTAGGGGCATTTGCAATTTACTCTAAATTTAATTTCTTTCTAGTTGTCGGGTTTTATTTCTAGTATT

AAGCTTTTAAATTATTTTCATTGTTACAATTTTGGGATTTACAATATTAGCAATTGGATTTTAGTATTCTCGTTTTCGAT

TTAAGTGGTTTAATATCCTAAAGCTCTTGGGCTAGCCGCATTGCTGTGTTATATCCTAATTTTGGAACACAAAGGAAAAC

AATAAAATAGGCGCTGATTAAAAAAAAAAAAAAAATGAAGTATTTTACCTCCTTTAATTATTTCTCCAAATTCCTAAGTC

TAAAAGTTGTTTTGTCCGATCCTGTTTCAATTGCAAAAATTGAAAAAATCAAACGAAATTAATGAAAATGGCTAACATAT

GGATTGATGTTGTGGAGAGATCAATGTGATTCGCTCTTCAAGGGCAAAAGAAACCTTTGAAGGTAACTCCATGCTTATGC

GTACATGGTGCATAATTGTTCTTTTATTCTCTGGTTTACTTGAATTAAGGATTCCGTGTTTGAGTCTGTGTTTGTTAAGA

GACAAAAAATTGGGTGTTCTGAAGCAGTTCGTGTTGGGTATATGCTTTGTGCTTTGCGATTCCATTTTCCCATCAGGAAA

AGTTAGAGAACTTCAATTGCTACAAAAAACTATCTTGTCCTAAAAAGATAATGTCCATCTTTGCTAGTAGAGTCAAGTTA

AGGACCAAGTTTGCTATTTTCTCTATTTTTTAATCATTGTATCTTTAAATTCATGTAGAAATGCCACGTGTGTAAGAGTG

AAAAAAAAAATACAAATACCACATAAGAAAAGAAAAATACAAGTTAGAAGTGGGCTGAGAAATATGTCAACATTGGACCT

AAAGACTAAATTTGCAAAAAATTAAAATGCTAAAGGATGAATTTTTCATACCAATAAATTAATGTCCATTTCTTTTTCAG

TGAGATCGATTTACAAGATGGATTTTACAATTTTTCCTAGTTTTTAATATGAGTACTTCATTTAAATAACTTTATTCATT

GGCATATCTAAACATCCTTTAGGTAAATTTATTTATTTAGATAGAGAAAAAGGTGTAAAGTTACAATTAATTAATTAGAG

TTCTCTTCTCTTTAATTCTTCCTTTTTTTACACCAGCCAAAAAATTATCAATACTCATGAGTATTCGTGGGTAATTTTAC

CCCGTGACTTTTGTACACAACCCCTGAGTAGCCATAGGTTATATATAATTTTTATTTTTATTTTTTTGTTTTAAATATTA

ATTTATTAACCTAATTCTGCTCATTTTCTATCATTTATTAATGTAACACTAGTCAATATAATATGTAAAATTTATAATCT

CTTGTATTAGATTTGTTTAACTAATATTTGAATGATATTATTTAAATTATACATTACATTTCAAGTTAGTTTCAAATAAT

AATAATGATATTATTATTATTATTATTATTATTATTATTTCTTTTTTTGGGTTGTCGTGAACCTAAAACTGTACGCATTC

ACCGGCTACAGCTGGTTGTCCCTAGACTTATTTCTATAAATACCAAAGAAAGCTATTACATTTCA

> CbuCOMT 6

AGAAATTTGATTTGAAGACCCAATTTTCTATGAGTCATTTAATATACTTTGCATGCTGATGAAACATCATTTTAGAATTG

AGGATCCCAACTATTATAAATATGTTCCTATGGTTCTCCTATGGTCGTGCTTCTAATAATTTTGGTCTCTTTACATGCAA

ACTTAACGGTCTTTTGATATGTTTTAAAACATCTTTATATAGCGTCTTTTATGGTGCCTTTGAGCTTTGATGCACATTTA

TTCTTGAGAGAGTTTGAGAAAAAATTATTGAGAGAATTTGTTTACGCCTTTTGTAATTCTTTTAGAAAAGTGGTGTTTCA

ATTTCTTCTGTACTCCCTTCTCTATTTGATGTTAGTAAATCTCCTATTTGCTAGTGGAAATAGCCCTGTTTTTGGTGAAC

CAAGTAAATTAATTTTATCCACTTTGTTTGTCCCGTTTCTCGTTATGTTTTTTGTATGCGCTTGTCACTTGTTTCACAAA

AAGAATAATATTTTTTAGGATTTGTTTTTTAATGAAAAATAATTGTTCTGATTTTTTAGTTAAATAAATAATTATCCCTT

AAATAAATCTCAAGTTAAAATAGATAATTTTTCAATTTAAAAATAAAAATTAGTAAATTTTGTTTTGTAAATTCAAATAA

ATTACATGACAAATGAATTTATAGTAATTTATATTTTTGAGACATGACACAAACTTCTACGGGAGGCAATAATGAGGAAT

TTTTCACAGTGGGCAAAAGCCTAACGGAGCAATGCCGCGTGGAGGTAGAAGACCCAGGGTCGTGAACTTCTTTTCCAGAG

AAGAAGCATTTACGGTATCTAGGGAGTAAGTATCGGCTAACAGACTCCTATTTCTCAATGATATTCCACAATCAAGACTA

CTGGTTGAATCCCATGAAACCATTTCATAGAAGTTTATTGATATCTTCTTTTTATAAAGCAAATCAACTTCGATTCTTGA

ATAATCCACATCACTTCTACTTCTATTATAATACAAGATTCCACTTTTCTGTGGAAAAGACCCGCATCAATAATTATGAT

TTTATTTTAATTAAATATAAGAATATAAAAAAAAAAAGATAAAAAAAATTATAAGTTTTGTAGCTTAATAAAATAACAAA

GACGAGATATAAAAATCATGATAAATAATTAATTAAAATAGTAAAAATATGGAGTTAAAAGAAAAGCTGAAGACAATAAA

TTGTTGTATCATTTTCAACAACATTTTAATTTAAATGAAAGGCATAAATATTTTTATTTTATGTTTTGTAACATAATTAA

TAAATAAAATACAAAATAATATCATTATTTAAAATAAAATATGATTTTTTTCAAAATATGTTGCATATAGTCTTCCTGGA

GGCATTTTCCACAATAAAAAATGGTAAGAAAAAATCTTATATATAGATCTCATGGGGGTATTTTGTCTACAAAAAATGGG

TCAATAGTAGGTCAAAAATTAAAAGAAAAATAGGAACAACAATCAAATTTCAATTGACATTGTCAATTTCCGTTATACTA

ACTGATGGTAGAGAATGTAATGATAATTATTCAAATCTCATTATGTGTTTTGATAATTATGTCAAACTTTAATGAGCATT

TAGGTTGTCATCCCTCATTGCTACCAACTCTTTCTAGAAATTAAAAAGAATTAAAGACCACTTGCTCTAACTTCCATCTC

ATTGTAAGGATAGAAAATATAAGGCAAGCAATCAACGTATATATAGAGAGAGGGGGGGAGGGAGAATTACCTCAACCCCT

GAGTTTGATATAATTATTAAAATATTTTATAAACTTTGGATAAATATCATTACACTCCCTGAAATTTGGATAATCACCAT

ACACCTCCTAAAAATTTTATACATTACAAGTAGACCCCCTAAATAAGGTGTTATAACTGTAATTATGTATACGTTCAGGG

GGTATAACTGTAATATATGCAATAGTTGTATAACGTTCAGGGGGTATAACTGTAATATAACTGTAATTGTAATTATGCAT

TAGTTGAATTGTACAATAGAGGTGTGTAATGATAATTATTCATACCTCACGGATATTTTGATAATTATGATAAACTTCAA

TGGTTGTAGAGGTAATTAATGATATATATATAGGACTAATGACAATGCAAATACATCTGCTACAATTAAACT

> CbuCOMT 5

GAAGAAGGGTTGGAGGAAGAAGAAGAGAGTGGGGAAGAAAGAAAGAAATATATAAATATATATTTAGGGGCAAAATAGAT

ATTTTCAAGATTAAAAATTAACACCACTCTGACAAAGTAGGATGTTACATACACGCGCCAAAAATGAGTGTCGGGTGTCA

TTTTTCATGAAACGTGAGATTTTTAGGATTATATTTAACGACACAAGAATTTATGTGCTAATTTGCGATTTCACGTGAGG

CTTCATGCAATTCACTCCTTTCTTATTCTTATAATTAGCCCAATTTTTTATGGGATTGGGTGTATTACGTCTGGTAATTG

TTCCAACAGTATAGGCTGAACTTTGTCCGTTACCACTTATCAATAGCGACAATTCAGATAAGTTAAAGGTGGAGAAGTTG

AAAATGTCAAACCACTTTTGAGGAATCGGATCGAACCAGTTGATTCAATCGATGATCAAACAAGGATTCAATCCGATTTA

ATATAAAAAATTGGTATGAATTGATAAAAACTGGAAAAAATCGATTAAACCGATCAGCCGGTCAAACCCATTTGCAACTT

TTTTATTTTTTTAATTTTAAAACTCTAAATTTAATTTTTAAGTTTATAATTTTTAGTGTTTTAGAATTTAATGTTATGAT

TTCTTTATTTCTTCTTTCTTTAATTCTGTCTTTATCTTTATTTCGCTTCTTTTTTTATTTCATTTTCTTTGTTCTTTTAC

GTTTCTTTTTGTTTAAAAAAAAAAAAACTTAATTCATGATTTGACATTTTAAATTGATGATTTTAATTGATGTATGAGTA

TTATTATGAGGTAAAACTTGAGCACAATATTCATCGACATGAGGCAATTTTTGTTCTTTTTCTTATATATATGTGTGTGT

GTGTGTGCATTTTTTTATATTATTTAATATATATAATATTAAATATAATTATAACGTCACATTAGTTAGGCAAGTGATAA

AATCGACTGAACCATTAACCAATAAGACTACAAATTCTATTACCAATCCGATTTTTCAAACATTTTTTTTAGAGTGACTA

TGATAGTTAATTAAATTCACCAAAATTGAGCTATTTTGACAAATAATAACAAAAAGAAACTTCATTTAGAAATTCAGCGG

CTCAAAAAACAAAACCAACAGGGAATCATTACAAACCTTTATTACATAACCAAACATACATTGCAAAGATTACTTGCGCA

TAATAAGTTAAATAAGAGCATTATATATCTTTAATAATTAGGGATAGGCCTCAATTACAGAGACAAGAGTTTTAATATTT

ATAAGGTTGTGTTTGCTAAAGCCCGCTGCCTTCAGAAGATGTGCCCATTCTTTGTATGTCCTCTCTTTGCCTTCGATCAA

GTAGGCCCGTCATTACCATATCCAGCGACAAACGGGCACCTGTGTACTCATCTTCTTCGCCGTCTTCGTTTATGACAACT

TCTGCGATGATCACTTTTCCGGTGTCTGCCGGAACAGCTTCTTTGCATTTCTTGAGAATGTCTATGCACGCTTCGTCGCC

CCAATTATGCAATGTCCACTGTCATAAAATAGTATGAGAAAACGCTTTTATTAATAATTAATGGAGAATTTGTGATGGAA

AGGGTTTCTTCGTAAAAAGATTATATTTGTGATGGAATTTTGAGCAAATTCAGATATATAACTTATTAAAATATATTATG

TACAATATCATAATATATATAAAAAAAATTAAAGTATTATTTAATATATGTAAATATATGAATTTGTTCATAGAAGATGT

TGTATGTATAGTAATATTTTAAATATTATTTATTAGATATGAACAATGGATATAATTGAATATGTAAGCGGATATATCCT

TTACAAAATATATTCAGAGCGATTTAAATATGCGGTTACAATATTCGTACCCGAATACTCCTAATTGAATACAGATAATT

TTCACTTTAAATAAATATGAACAATACTTCGTATATACAAGTACGAACATGAATAGTAATAGAATTTTCAAGTATCCATT

TACAGTAACAATTGTCCCCAACACATATCAATAGACATTCATAAACACACAACACAAGTTAGGTCAATCAAACGACTCTA

AATCATTAAAGTAAACATACCAATAAATCTAAAGTAACCACTCTAAACCATCATCATTAACGCGTAC

> CbuCOMT 4

CACACACACATATTCTTTTCTAAAACTGTTCTTGGGTTGAACTGGTCATTTTACATTCCAATTTATTTGGTACTTTTGTG

ATAGGCCACAAGTGCTGGAGGGAAAGAGCAAGAAGCCATTAACTTTCTAGAAAAGAAAATGAAAAATGATCCTGCATTCT

CCTATGAGGAAACTGTACAGGTAAAGAAACTGCCTAATCTAGGCAAGCCCTTTATAAGTGTTTGCAATTAGGATTCTGAC

CTGATTATTTGAGTATATGTGCCAGGTGGTTAACTTGATAACTTCCCTATAGCTATTGATGAGTATATCACTTCACCTGC

CTGCATATTCAGAATACACAAAATTTAAATATGCGGCTGGATTTAATGACTATATTTATTAGTACATATGGTGTACCTAT

GAAACCTGGATAATCTGGACTTACTATTGTTGATTGATGTTAGCCTTTGCTATTAAGCTTTACTTTCTATATACAATGCT

CTTGACTGAATATATTGTGTGGTTCGCTTTCTTTTGAAGTTGGGGATGAGTTTGATTTTGATTTCTAACTGTCATTAAAG

CGAAGGTTGAGTGTTCTAATATTTAAACTGTTTCTACTTGTAGACTGCAATTTCAGCTCTGCAATCTGTCCTACAGGAGG

ATTTCAAGGCCAGTGAGATTGAGGTAAATCCTTTTTGTGTTTTAGCCTGAAAATGGGCCCATGCTCTGTCTCTGTCTCTG

TCTCTCCGTCTCTCTCTCTCTCAACCCCCAAGCCTTCTTCCCCTGATATAGAAGAACTTGCCCATTTAAGAGATAAAAGT

AAAGTTCATCAACTTGCTTTAGGGATAGCTTTCTTGTTTGTTACTATGTTTTCTTTGGCAATGGGCATCTAAATTTAATG

TCATACTCCTTATAAAGTATGACCTTCCGTTATCTTTTCATAAGTCTGACTGTATTGACAAAATTTAGTTAAACATCAAT

CCATTATCTTTTCTTCTCTCAAGTTTGAGAACAATACATTTGCTGCTGCATTAAATCCTTTTGGTCATTGATAGGTTGGT

GTTGTGAGGAAAGAAGATCCAGTTTTCAAAGTTTTATCAACTGAAGAAATTGATGAGCACCTGACTGCAATCAGTGAGCG

CGACTGATCTTGTTGGGTTTGCGAACTGTGTGCTTATAATATTTCAATGTGAACCATGTCTCATGATGGATGGATAGAGA

GTTGACGGTGTTCTACATTTATTCGGATAATCAAACAGTTCCATTAGTTTTTGTTGCTTGAAACCCATCCATGTTCCGTT

ATGAAATTGTAGCTTAACTTCATGGTTATGATATTTGCCTTTGAACATGGGTTGGTAGAACTTATTATCCTGCCATAATT

TTACAGCAGTCATGAAGTACCTGAGTTTGAGTCCCTAAATTATTTGAATGCTCTCTATATGTGCTCAAAATTCAAGTTTG

TAGACTTACAGGGTTGAGTGAACTAATATGTTCTTGAAAACATCTTATAAGCCGATGCAAATGTTTGGTTATTATTATTT

TATTTTTTGGAAGAAAAGATCGTATAAGCTTTTGAATAAGATGAATAAGATGTTAGGATGTTATAAGTTCTTATAATAAA

AGAGTTCTTAATTTATTTTTTGAATCTTTATATGCTCCCTGTTTGTAATATCAATTACAAAATTATTATTATTAATATTT

ATAAGCTTCAAGATTTTATTTTATATGTTTTAATAATTTATTTTTAAAAAAAATCTAAGATATTATAAGTTTCAAAAATA

TTTTATAATATGTAGGAATTTGAAAGATATTTTAAATGAGTTTAACCACAAGCCCTCTTATTTAGAAGAGTTCACATTAC

TTGCAGGTCATGTATGTTGGTGCAAATCAAAGGTAATATTGTTCACTTTGAATTTTTTATTTCAATTCGTGTTTCATTTA

GTATTCCATTTTATGGATGCTTGTCAAGTATATGACATTTACACCCTGTTCGTTTATAAGGAAAGATTTAATACACTGAG

TATGTATTTAATACATGAAATATGAGATGAAACACTATTTTAAAATAAAGGATCTTATCCGAACCTCTCTTCTAATATTG

ATGATAATTCTAAGCTTGGTAGGAATTAAATGCTGAAAAGTTGACAAAAGCAATACACAAT

> CbuCOMT 3

TCCAAGAAGCCACTTAATGACAATGTCAACAATTTCCGTTAGTAATTTAACGGTGGGACTTCAATTAAGACATTTTTTGG

AGTTATGAGACTAAATGTGAGAATTGTTTTATAACAGGACCAAGAAGAGGAATTGATCATTATAATAGGACAAAAAATGC

AATTTACTCAAAATTAAGGATTTTATTAAAATATTGGATAATCCATTATTATGTTTTAGAGTAATATAGTAATTCAAAAT

ATTAAGAGAAAAAAAAATAGTTTTTGTCCTATAACTATATTATTTTCTACTTAATAACCTGTATCTTTTGAATTTCAAAT

TCCGGTCCTCCTATATCTTTTTGTCGATTTATAAAAAATGCTTATTTCGCCAGAAAATCCTATGTGGTACCAAAAAATAG

CTGGAAAATTTGACGTCCAATAATGAGAGACACACACCAGCCGTGCCAAGTTAGATTTTCCGAAGAAATGAGCACTTTTT

GTATATTGACAAAAAAATATAGGACTGAAATTTTAAATTTGAAAGATGTAGGTCATTAAGTAGAAAATGACATAGTATGA

GACAAAAACTGCATTTTTTCCAAATATTAAAACTGTTGAAAATCATTTTTTTTTTACGAATACCTATCATCAACATTTAA

ATAATTTTTTACTTTTAATTACAAACATTCTCAATTACTTTAATTCAAAATAAGTTCCTTAAATACATACTCAAAGACAC

TCTCTCCTAACCTCCCAACCAGTTTCTGTGGGCAATCAACTACAACGTTTTAATAAATGAAAGGGTTAGATACATTTGCA

CTCCTGAAGTATGTATGGTATCGCATGTACATCTTCAAACTTTAAAACGTACCAAAAAGACCCTTGCCTATACATTCATG

TGAATTCGTGCATCGTATATTCAACCCTAAATGAAATTAAGAGAGAGTTTCTTGTTTTCCCTGCCATATAAATAATTACT

ATAGATTTAATTAGATCTCAGTTATCTAAGTCTTTTATTTCTTTTAAATAAATATTTAAAATCATTATCTCACTGACCAG

TCACTACCATTTCTTAAAGCTATTTTCTAATCTTTCCAGTGACTAAGGGCCATTGTGAGAGTGTCACATTGATTAAATGT

ACGAGTAACGATCTTTAATTCTCACCACTTAAACTATCTTTTGTAGTGAGTTAAAACTTAAATTCAGCGTACATTTTTAG

TATATGTTACTTTTATTTTTGCACAACTCCAAACAAAGATTTTTACTATTTTCAATCAAAATTTTCTTTTAAAAACACCT

TCTAACTTAAAATTAGGAATAAATATCAATTCAACACCCTTAAAATTTAACCTAATTAGAATAACACATCATGTGATTTG

ACAAATTACAATAACACCCTTTATCGTTAGTTTAGCTAATAAAAAATGTAATGGAATATATTTGTGGTTTGAGAAATTAC

AATAATATTTCCCATTAATTGCAGGGTATGTTATTATAATTTTTTATATCAAAAGGATGTGTTCTTAATGTAATTTTTCA

AACCATAATTATATATCACTCCATTAAGTTTTCATCATCTAAACTAATGAGGGGGCATGTTATTATAATTTTTCGAATCA

CAAATGTTTTTTATGTAAATTATCGGTGTTTTCAAACTTTAATTCCATCCCTTTGGAAATAGCCATTTTGAGTCGCATAA

TTTTTCGGCATTTGAAAACTAAATCCCGTCGTTAAAATTTGGACGGAAATTGCAGTAGGCACGTGCTTGCTCCGTTAGGC

ATCTTTTTTTTTTGGAGAAAATCACAATTCAATTTTTTTACCAAGAAAAATTGCAATTTTGGTTCCACAATTGTGGATGT

TTTCGAATCTTAATCTCATCTCTTTTGAAATAGCTATTTTTAGTCTCATAATTTTTCTGACCCTTGCAAACTATGTCCCG

CTGTTAATGGAACAAGCATGTGTTTGCTGCAATTTCCGTTTAAATTTTAACGATGTGACTTAGTTCACAAAAGTTAGAAA

AATTATAGAATTAAAAATGATTATTTCAAAAGGGATGAGAATGAGGGGAACCATTACTCTCCTATTTTTATGAAAATTAA

TAATATAAAGTCAAATTTTTATTTTTATTTTCAAATGATGAACTATTATTAAA

> CbuCOMT 2

TATATATATATATCTAATCTTATTTAATTATAGATCATATTTTTAAACTCCTAAACATAAGGCATAAATATTTTCACCAC

ATTAGTTTTGGATTATTTATTACCGCTTCCACTATAACTATATTAAATTTCAGTTATTCACTACTACCACTGTAACTATT

AATATTTTAATTGTCAAAATTCAATTATTATATGATTACTCATTTTGATTAATATTTGCCAAATTTTCCATAACAAATAG

ATAACATGTATTGCATGTTCTTTTTTTTTAAAAAAAAAAATCATTGCAATTAATAGTTTCTTAACTTGTTTTCTATATAT

TTCAATTTTTTTTTTTTCAAAAGATAACGTGTTTTAACAATATTTCTTTATTTCAAACCATTTAAAAGCACTATTCTGCT

TCATCTTTTCCAACAAAATTAAGGATTTGATTAAAATATTGGATAATCCTTTATTATGTTTTAGAGTAATATAGTAATTC

AAAATATGAAGGGAAAAAAAATTGTTTATGTCGTATAACTATGTCATTTTTACTTAATGACCTGTATCTTTGAATTTCGA

ATTTCAGCTCTATATCATTTTGTCGATTTATAAAAAGTGTTTATTTCGCCGGAAAATCCTATATGGTACTAAACAATCGC

CAAAAATCTTACGTGGCACCAATAATGAGAGACACACACCAACTGTGCCAAGTTATTTTCCAGTAGCTCTCGAATGTATA

AAATTTTATAGGGCACCAATAATGAGAGACGCACACCAGCCGTGCTAAGTTATTTTTATAAAATAATTGCCCTACATCTT

TCAAATTTAAAATTTCAGTCTTATATCTTTTTGAGGATGGATATACAACACCTTGCACACTACAGGGGTGCAACATATAT

TTAACCCTAAATGAAATTAATTAAGAGAGAGTTTCTTGTTTTCATTGCCACATAAATAATTACTATAGATTTAATTTGAT

TTCAGTTATCTAGTTCTTTTATTTCTTTTAAATAAATATTTAAAATCATTAGCTCACTGACCAGTCACTACCATTTCTTA

AAGCTATTTTCGAATCTTCCAATGACTCTCAAGGCTATTGCGAGAGTGTCACCTCTTAAAACTTAAATTCAGTGTACATT

TTCAATATATGTTACTTTTATTTTTGTACAATTCCAAACAAAGATTTTTACTATTTTCAATCAAAATTTTCTTTTCAAAA

CACCTTCTCACTTAAAATTAGGAATAAATATCAATTCAACGCCCTTAAAATTTGACCCAATTATAATAACACCTTCTGTG

GTTTGACAAATTACAATAACATCCCTTATCGTTAGTTTAGTTGATAAAAAATTTAATGAAGTATATTTGTGGTTTGAAAA

CTTACAACAACATTTCCCATTAATTGCAGGGTATGTTATTATAATTTTTTATATCAAAAGGATGTGTTCTTAATGTAATT

TTTCAAACCATAATTATATATCACTCCATTAAGTTTTCATCGTCTAAACTAATGAGAGGACATGTTATTATAATTTTTCG

AATCAAAAAAATTATTTATGTAAATTATCGGCGTTTTCAAACCTTAATTCCATCCTTTTGGAAATAACCATTTTGAGTCG

CATAATTTTTTCAACCTTTGAAAACTAAATTCTGTCGTTGAAATTTAGACAGGAATTGCAGTAGGCACGTACTTACTCCG

TTAGGCACCTTCTTTTGGAGAGAAAATCACAAATCAATTTTTTTACCAGGAAAAATTGCAATTTTGGTCCCACTATGGTG

GGCGTTTTCAGACCTTAATCCCATCTCTTTTGAAATAACTAATTTTAATCTCATAATTTTTCTAACCCTTACAAACTAGC

TATGTCCCGCTGTTAATGGAACAAGCACGTATTTGCTGCAATTTCCATCTAAATTTTAACGATGAGACTTAGTTTACAAA

GATTGGAAAAACTATAAAACTAAAAATGATTATTTTGAAATGGATGAGCATAAGGTCTAAAAACACTCAGTGGGGGCAAA

TTTACAATTTTCCCACCAATCCTTACGTGTTGATTTAATTATCCCCTGAAATCATGAAATCTCAACCATTACTTGAGAAT

TAATAATATAAAATCAAATTTTTATTTTTATTTTCAAATGAAGAACTATTATTAAA

> CbuCOMT 1

TCTTTAAGGTCAAGGTCGAGCTCGATGAGATCAGAAATATTGTTCTCGAGCTCTGTTTGGCTTGCTCATTTGGCTCAAAT

TTGTGACAAAATCATACGAGCTCAAATAATTTATTATGTTCAAGTAATAAGTTTAAGCTCGAGCTCGAAAAAAATTAATT

ATTTTATAAATAATTTAATTTAATTATATATAAGTTTATATTTATACATGTAAATATTTTTTTTAAAACATGGACTCGCA

AGCTAGCTCGTAAACCTACAAACAAAAATTATTAGGCTCGATCTTAAATTTTTCGGACTCGAGCTCAAACATTTTTCGAA

TCGTCTAATCAAGCCCACGATCAGGCTCGATTCGTTTGCATCCCTAATTATGATCCGTACTAATTTATTGATAATCTCCA

TTAAAAAGAAAAATGAAGATGCATTATGCATAGATGGGTTCGTTGGCATTTATGATTGAAAAAAAAAAAAATGCATTATG
